# Therapeutic delivery of microRNAs discovered to target deregulated glioblastoma pathways inhibits tumor growth in mice

**DOI:** 10.1172/JCI195639

**Published:** 2026-06-30

**Authors:** Shekhar Saha, Ying Zhang, Myron K. Gibert, Collin Dube, Farina Hanif, Elizabeth Qian Xu Mulcahy, Sylwia Bednarek, Yunan Sun, Pawel Marcinkiewicz, Xiantao Wang, Gijung Kwak, Ahsan Polash, Haolin Li, Kadie Hudson, Manikarna Dinda, Tapas Saha, Matthew McCord, Fadila Guessous, Nichola Cruickshanks, Rossymar Rivera Colon, Lily Dell’Olio, Rajitha Anbu, Wenjie Liu, Songy Choi, Benjamin Kefas, Pankaj Kumar, Alexander L. Klibanov, David Schiff, Jung Soo Suk, Justin Hanes, Jamie Mata, Markus Hafner, Roger Abounader

**Affiliations:** 1 Department of Microbiology, Immunology, and Cancer Biology, University of Virginia School of Medicine, Charlottesville, Virginia, USA.; 2 Department of Biochemistry, Dow International Medical College, Dow University of Health Sciences, OJHA Campus, Karachi, Pakistan.; 3 National Institute of Arthritis and Musculoskeletal and Skin Diseases, NIH, Bethesda, Maryland, USA.; 4 Department of Neurosurgery and; 5 Medicine Institute for Neuroscience Discovery (UM-MIND), School of Medicine, University of Maryland, Baltimore, Maryland, USA.; 6 Department of Chemical and Biomolecular Engineering, School of Engineering, Johns Hopkins University, Baltimore, Maryland, USA.; 7 Department of Biochemistry and Molecular Genetics, University of Virginia School of Medicine, Charlottesville, Virginia, USA.; 8 Swiss Re, Healthcare, Bengaluru, India.; 9 Department of Pathology, University of Virginia School of Medicine, Charlottesville, Virginia, USA.; 10 Laboratory of Onco-Pathology, Biology and Cancer Environment, Faculty of Medicine, Mohammed VI University of Sciences and Health, Casablanca, Morocco.; 11 Pharmacy, University of Virginia, Charlottesville, Virginia, USA.; 12 Bioinformatics Core, University of Virginia School of Medicine, Charlottesville, Virginia, USA.; 13 Division of Cardiovascular Medicine and; 14 Department of Neurology, University of Virginia, Charlottesville, Virginia, USA.; 15 Department of Neurosurgery, School of Medicine, Johns Hopkins University, Baltimore, Maryland, USA.; 16 Center for Nanomedicine at the Wilmer Eye Institute, Johns Hopkins University School of Medicine, Baltimore, Maryland, USA.; 17 Department of Radiology and Medical Imaging, University of Virginia School of Medicine, Charlottesville, Virginia, USA.; 18 Comprehensive Cancer Center and; 19 Center for RNA Science and Medicine, University of Virginia, Charlottesville, Virginia, USA.

**Keywords:** Cell biology, Oncology, Brain cancer, Noncoding RNAs

## Abstract

Glioblastoma is a fatal primary malignant brain tumor, with an average survival of 15 months despite surgical resection, chemotherapy, and radiation therapy. Due to the concurrent deregulation of numerous genes in glioblastoma, molecular monotherapies have not improved clinical outcomes. Evidence suggests that targeting multiple deregulated molecules is essential for better therapies; however, this is limited by the lack of suitable drugs and increased toxicity of combination therapies. To address this, we hypothesized that miRNAs, small gene-regulatory RNAs that suppress mRNA, could simultaneously inhibit multiple deregulated genes in glioblastoma and be used for more effective therapies. We identified regulatory miRNAs — those that target several deregulated genes in glioblastoma — using a combination of PAR-CLIP screening, TCGA data analyses, and an algorithm to rank target importance and miRNA therapeutic potential. We selected 2 tumor-suppressive miRNAs, miR-340 and miR-382, and 1 oncogenic miRNA, miR-17, and showed that they targeted critical glioblastoma pathways and altered cell growth, survival, invasion, and in vivo tumor growth. We developed and successfully applied a miRNA therapeutic delivery approach using brain-penetrating nanoparticles combined with MRI-guided focused ultrasound and microbubbles, to inhibit established tumor growth and extend animal survival. This strategy offers a promising approach for translating miRNA-based therapies into clinical trials for glioblastoma and other cancers.

## Abstract 2

We developed and used new computational, experimental, and therapeutic approaches to identify and therapeutically deliver master regulatory miRNAs to inhibit the growth of glioblastoma, the most common and deadly primary brain tumor.

## Graphical abstract

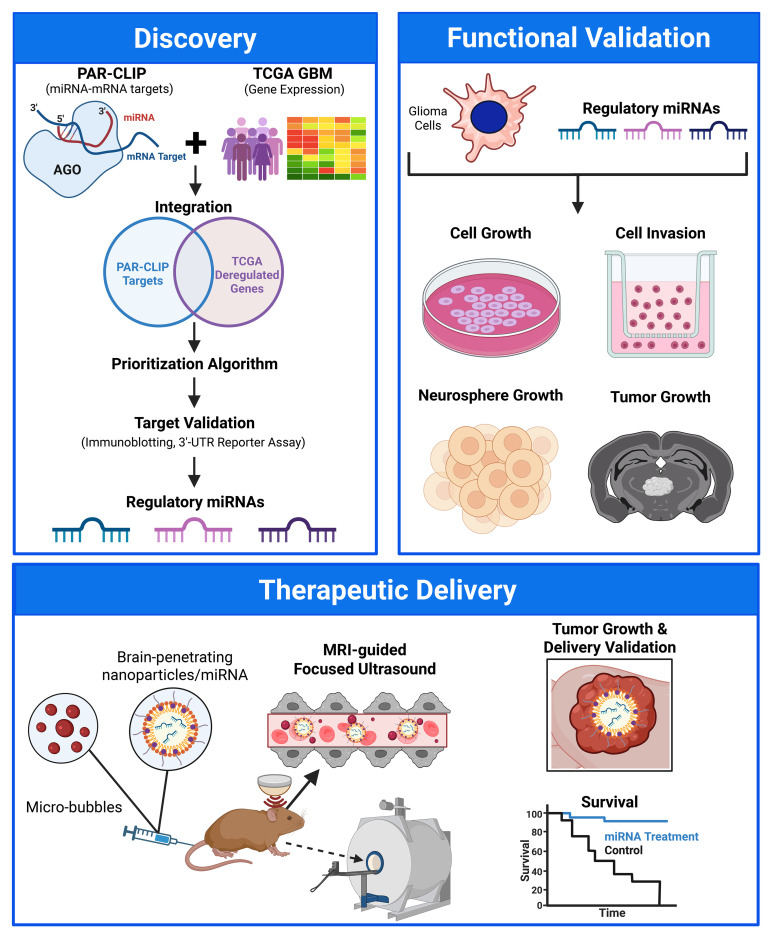

## Introduction

Glioblastoma is an aggressive and fatal primary brain cancer that remains a formidable challenge because of its heterogeneity, therapeutic resistance, and invasive nature (1, 2). Despite advances in therapies and clinical trials, the standard treatment of maximum surgical resection followed by radiation and temozolomide chemotherapy extends patient survival to only a modest 14.6 months (3–5). The Cancer Genome Atlas (TCGA) and other studies comprehensively analyzed deregulated gene expression in several hundred glioblastoma tumors and described the concurrent deregulation of numerous genes in any single tumor (6, 7). Because of this multigene deregulation, molecular monotherapies have failed to achieve significant improvements in clinical outcomes. Several lines of evidence suggest that simultaneous targeting of several deregulated molecules is required to achieve better therapies (8, 9). However, the simultaneous targeting of several deregulated oncogenic drivers using conventional drugs is severely limited by the fact that the drugs needed to simultaneously target many deregulated molecules do not currently exist, and because combining several drugs in a clinical setting leads to an exponential increase in toxicity. The goal of this study was to identify regulatory miRNAs, defined as miRNAs that target several deregulated genes in glioblastoma, and deliver them or their inhibitors using what to our best knowledge is a new approach to simultaneously target multiple deregulated molecules for glioblastoma therapy.

miRNAs are small noncoding RNA molecules that span 19–24 nucleotides. miRNAs exert their effects by incorporating into argonaute (AGO) proteins (4 AGOs in humans) and guiding them to target mRNA via seed-pairing predominantly to the 3′-untranslated regions (3′-UTRs), and less commonly to the coding sequence (CDS) and 5′-untranslated regions (5′-UTRs) of target genes. This facilitates the recruitment of effector proteins and the assembly of miRNA-induced silencing complexes (miRISCs). The miRISC induces either mRNA degradation or translational inhibition (10). miRNAs play pivotal roles in regulating various cellular processes, including proliferation, invasion, apoptosis, and differentiation (11, 12). Dysregulation of miRNA expression is a hallmark of various cancer types, where miRNAs can function as either oncogenes or tumor suppressors by suppressing mRNAs of tumor suppressors or oncogenes, respectively (13–17). Importantly, because miRNAs do not require full complementarity to inhibit targeted mRNAs, single miRNAs can target and simultaneously inhibit numerous genes (18, 19).

We defined “regulatory miRNAs” as those that target several deregulated genes in glioblastoma. We reasoned that we could identify regulatory miRNAs and then use them for glioblastoma therapy. Using them as therapeutic agents would theoretically be equivalent to using a combination of several drugs that target deregulated glioblastoma driver genes. To find regulatory miRNAs, we first used photoactivatable ribonucleoside-enhanced crosslinking and immunoprecipitation (PAR-CLIP) to identify all targets of all miRNAs in glioblastoma cells (20). We then analyzed TCGA tumor data to determine which of these targets are deregulated in human tumors. We developed and implemented a computational algorithm to prioritize miRNA targets based on their relevance to glioblastoma malignancy. We selected the top candidate regulatory miRNAs, defined by their capacity to regulate numerous target genes and, therefore, exhibit strong antitumor effects when delivered as therapy.

A major challenge to successful miRNA therapy is delivery. The central nervous system’s protective blood-brain barrier poses a formidable challenge to drug delivery. Focused ultrasound (FUS) presents a noninvasive and reversible approach to transiently open the blood-brain barrier in animal models. This temporary blood-brain barrier opening facilitates drug delivery in brain tumors and other brain diseases (21). Recent clinical studies have successfully used MRI-guided FUS (MRIgFUS) with microbubbles (MB) to deliver chemotherapeutic drugs to human brains and treat neurodegenerative conditions such as Alzheimer’s disease, Parkinson’s disease, and amyotrophic lateral sclerosis. A second challenge to achieving effective gene therapy distribution in the brain is the extracellular matrix (ECM), a dense and nanoporous network composed of electrostatically charged molecules such as proteoglycans, hyaluronan, and tenascins that restrict the diffusion of gene vectors through steric hindrance and adhesive interactions (22). To address this problem, brain-penetrating nanoparticles (BPNs) consisting of poly(β-amino esters) (PBAE) that are coated with a high density of polyethylene glycol (PEG) were developed (23).

In this study, we integrated miRNA target identification from PAR-CLIP with gene expression and survival data from TCGA to uncover regulatory miRNAs in glioblastoma. Through this integrative approach, we identified 3 key miRNAs that collectively regulate a substantial number of glioblastoma driver genes: miR-340 and miR-382, which act as tumor-suppressive regulators targeting 87 and 79 genes, respectively, and miR-17, which functions as an oncogenic regulator targeting 22 genes. We performed extensive functional assays with these regulatory miRNAs in glioblastoma cell lines and patient-derived stem cells and demonstrated their ability to inhibit cell proliferation, invasion, neurosphere formation, and xenografted tumor growth. Importantly, we also demonstrated that these miRNAs simultaneously targeted multiple glioblastoma-regulating genes in different pathways, decreasing their protein levels. We then used MRIgFUS in conjunction with MBs to transiently open the blood-brain barrier, enabling the local delivery of systemic PEG-PBAE BPNs carrying lentiviral plasmids encoding miR-340 and miR-382 to achieve upregulation of miR-340 and miR-382 in preestablished glioblastoma cell and stem cell xenografts. This approach significantly reduced glioblastoma in vivo growth and improved mouse survival. This work describes what we believe is a conceptually new approach to glioblastoma therapy to identify regulatory miRNAs and use MRIgFUS with MBs and BPNs to deliver these microRNAs in vivo to inhibit glioblastoma growth. Given the availability of clinical-grade FUS and BPNs for brain applications, these findings pave the way for new miRNA therapeutic clinical trials.

## Results

### Identification of genome-wide miRNA targets in glioblastoma via PAR-CLIP.

**Figure 1:**
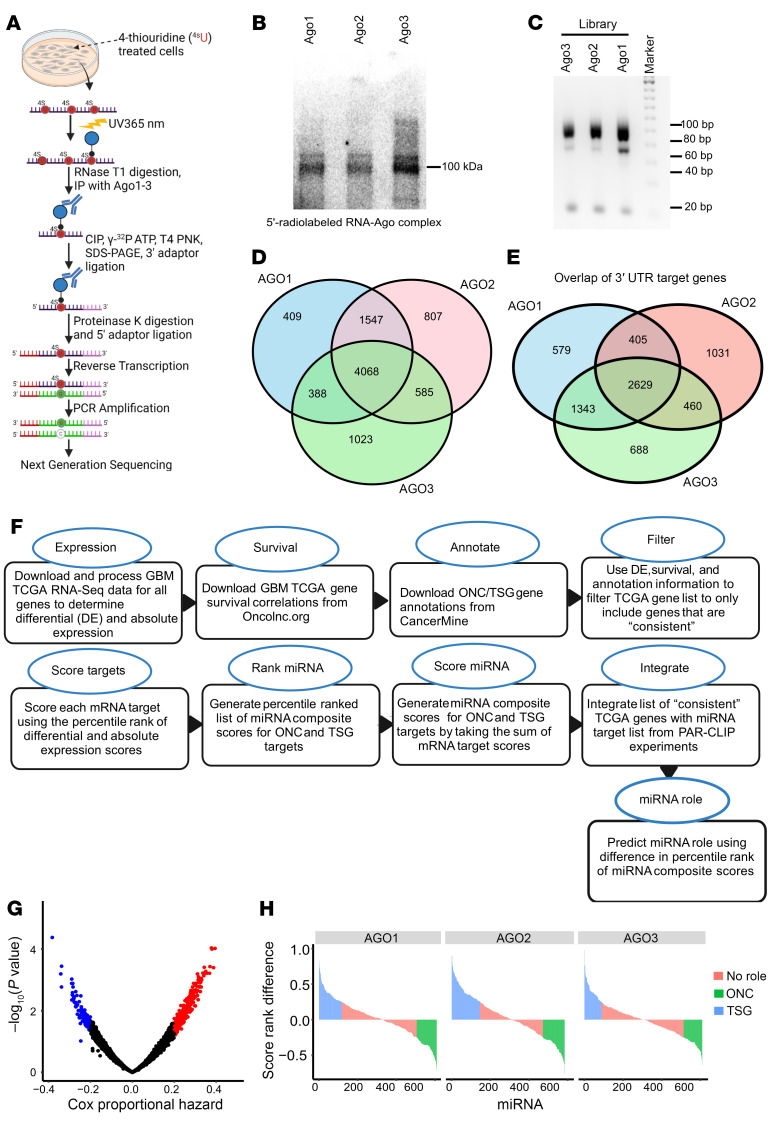
Identification and prioritization of regulatory miRNAs in glioblastoma. (**A**) Schematic overview of PAR-CLIP. 4SU, 4-thiouridine; CIP, calf intestinal phosphatase; PNK, polynucleotide kinase. (**B**) The phosphorimage of SDS-PAGE gel of RNA-argonaute (Ago) complexes labeled with 5′-32P that were immunoprecipitated with a FLAG-tag antibody. The complex is expected to appear near 100 kDa. (**C**) Agarose gel separation of PCR products from AGO1, AGO2, and AGO3 PAR-CLIP cDNA libraries. (**D**) Venn diagram illustrating the overlap in miRNA clusters identified with AGO1, AGO2, and AGO3. (**E**) Venn diagram showing the distribution of identified targets in 3′-UTRs from AGO1, AGO2, and AGO3 PAR-CLIP. (**F**) Diagram depicting the algorithm used for determining regulatory miRNAs in glioblastoma. ONC, oncogene; TSG, tumor suppressor gene. (**G**) Volcano plot denoting the statistically significant miRNAs based on Cox proportional hazard. Blue denotes downregulated miRNAs and red upregulated miRNAs. (**H**) Classification of miRNAs as oncogenic and tumor suppressive based on their targets derived from AGO1, AGO2, and AGO3 PAR-CLIP.

Several online miRNA target prediction tools are available, but they can yield false positives and do not always capture all relevant targets. To identify regulatory miRNAs in glioblastoma, we therefore first used PAR-CLIP to experimentally identify genome-wide targets for all miRNAs in glioblastoma cells (Figure 1A). PAR-CLIP was conducted in 2 biological replicates and independent experiments in U87 cells overexpressing FLAG-tagged AGO1, AGO2, or AGO3. The cells were cultured in the presence of ^4S^U, and the cross-linked RNA fragments bound by AGO1, AGO2, AGO3 were immunoprecipitated with anti-FLAG antibodies. Cross-linked RNAs were end-labeled using ^32^P, and immunoprecipitates were fractionated by SDS-PAGE. Ribonucleoproteins (RNPs) were visualized by phosphorimaging; the RNP corresponding to AGO was isolated; and cross-linked RNA fragments were recovered, reverse-transcribed, and deep-sequenced. Sequence reads were mapped to the human genome and grouped into clusters using PARalyzer to identify those enriched in T-to-C mutations, which are induced by cross-linking of ^4S^U-containing RNA (Figure 1, B and C, and Supplemental Figure 1, A–E; supplemental material available online with this article; https://doi.org/10.1172/JCI195639DS1). Resulting clusters of sequence reads were then analyzed to identify sites compatible with canonical seed pairing of miRNAs. We observed similar binding patterns for all 3 human AGO proteins (Figure 1, D and E). A total of 19,483 clusters were obtained from the 3 AGO PAR-CLIP libraries, with 6,412 for AGO1, 7,007 for AGO2, and 6,064 for AGO3. A total of 4,068 clusters were common across all 3 AGO samples (Figure 1D and Supplemental Table 1). Among them, 279 mapped to 5′-UTRs, 4,635 to CDSs, 4,750 to 3′-UTRs, and 513 to intronic regions (Figure 1E and Supplemental Figure 1, G–I). These clusters correspond to 4,583 transcripts, representing approximately 23% of all protein-coding genes. A comprehensive list of mRNA targets for AGO1, AGO2, AGO3, along with their associated 611 expressed miRNAs, is provided in Supplemental Table 1. These findings describe all mRNA targets of all miRNAs expressed in glioblastoma cells. They offer valuable insights into the genome-wide interactions between miRNAs and their targets in glioblastoma cells, illuminating the complex regulatory networks through which miRNAs modulate multiple targets.

### Ranking of regulatory miRNAs based on the importance of their targets in human TCGA tumors.

Having identified all targets of all miRNAs in glioblastoma cells, we next sought to determine the relevance of these targets, and consequently of their targeting miRNAs, to glioblastoma malignancy. This process is described in the Methods section (Figure 1F). Briefly, we analyzed TCGA data for the PAR-CLIP–identified miRNA targets in human tumors. We identified significantly deregulated targets (FDR ≤ 0.05) with a greater than 2-fold change in human tumors using TCGA database. The targets were then scored based on the magnitude and frequency of deregulation, as well as their correlation with patient survival, using a Cox coefficient threshold of 0.2 (Figure 1G and Supplemental Tables 2 and 3). Each target gene was assigned a composite score calculated from its absolute expression level in glioma samples and its fold-change relative to healthy brain tissue. These 2 metrics were independently converted into percentile ranks (ranging from 0 to 1), and their sum represented the gene’s composite score, with a theoretical range from 0 (lowest expression and change) to 2 (highest in both categories). For each miRNA, 2 aggregate scores were then computed: a TSG score, representing the average composite score of its oncogenic targets, and an ONC score, reflecting the average composite score of its tumor-suppressive targets. To enable comparison across the entire miRNA dataset, these scores were further normalized by converting them into percentile ranks across all miRNAs. To determine the functional role of each miRNA, we calculated the difference between its TSG and ONC percentile ranks. We added the scores for each target and then added all scores for all targets of each miRNA. We subsequently averaged the target scores for both tumor suppressor and oncogenic miRNAs across the AGO1, AGO2, and AGO3 PAR-CLIP datasets, then consolidated the comprehensive set of targets for each miRNA (Supplemental Tables 4 and 5). The final score for each miRNA represents its regulatory and therapeutic potential because it is a reflection of both the number of its targets and the importance of these targets in glioblastoma biology (Supplemental Tables 6 and 7).

Using this computational workflow, we identified several miRNAs that targeted many highly relevant genes that are deregulated in glioblastoma (Figure 1H, Supplemental Figure 1F, and Supplemental Tables 1–5). We designated these miRNAs as regulators because they can simultaneously target multiple significantly deregulated and biologically relevant genes and inhibit their expressions. This approach identifies miRNAs that are likely to exert strong therapeutic effects when delivered (tumor-suppressive miRNAs targeting numerous oncogenes) or inhibited (oncogenic miRNAs that target numerous tumor suppressors). The comprehensive list of miRNAs, their targets, and their scores can be found in Supplemental Tables 1–7.

### Regulatory miRNAs miR-340 and miR-382 bind multiple targets and decrease their expression.

**Figure 2:**
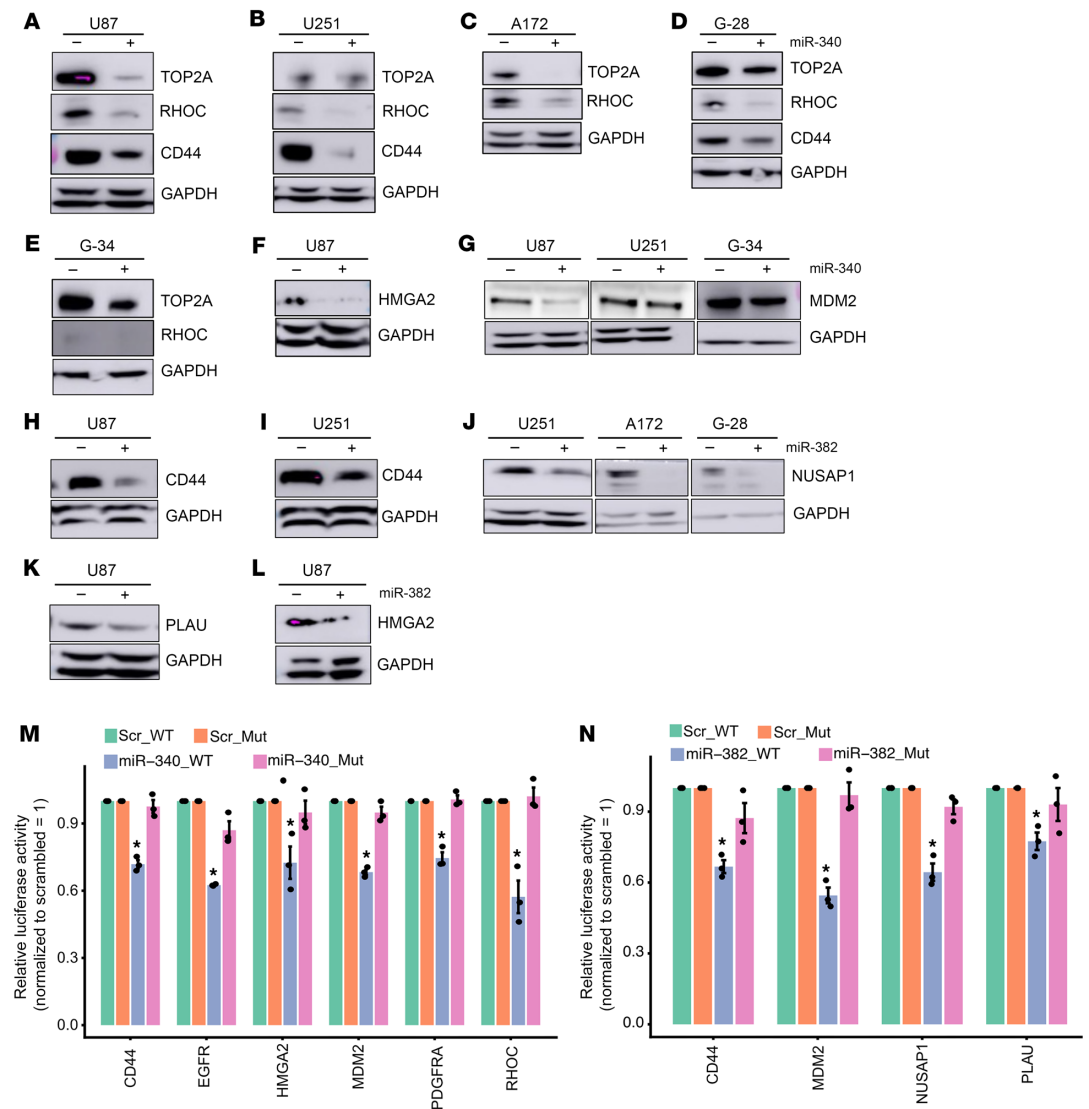
Validation of miR-340 and miR-382 targets in glioblastoma cells and patient-derived stem cells. (**A**–**L**) Glioblastoma cell lines A172, U87, U251, and patient-derived stem cell lines GSC-28 and GSC-34 were transfected with a scrambled negative control, miR-340, or miR-382. Immunoblots were probed with antibodies against TOP2A, RHOC, CD44, HMGA2, MDM2, CD44, NUSAP1, PLAU, and HMGA2. GAPDH served as the internal loading control. The data show that miR-340 and miR-382 downregulated protein expression compared with negative controls. (**M** and **N**) 3′-UTR luciferase activity assays in U87 cells were performed by cotransfecting cells with miR-340 (**M**) or miR-382 (**N**) and a psiCheck2 luciferase reporter plasmid containing the wild-type 3′-UTRs of target genes CD44, EGFR, HMGA2, MDM2, PDGFRA, and RHOC (**M**) or CD44, MDM2, NUSAP1, and PLAU (**N**) along with corresponding mutant 3′-UTR constructs in which the predicted miRNA binding sites were disrupted. The data show that miR-340 and miR-382 decreased luciferase activity for all respective wild-type targets compared with controls, while this repression was abolished or significantly reduced in the mutant constructs. The pink color on the immunoblots represents a higher intensity signal. Data represent mean ± SEM from 3 independent experiments. Statistical significance was determined using a 2-tailed Student’s *t* test, with *P < 0.05.

To prioritize regulatory miRNAs for functional validation, we incorporated evolutionary conservation as an additional criterion and selected the top 5 regulatory miRNAs from both the tumor suppressor (Supplemental Table 4) and oncogenic (Supplemental Table 5) lists for further analysis. Among these, we focused on 2 tumor-suppressive miRNAs, miR-340 and miR-382, because these miRNAs had high scores based on the algorithm described above. Our analysis determined that the tumor-suppressive regulatory miRNAs, miR-340 and miR-382, have 114 and 89 targets (Supplemental Table 4), respectively. From these, we selected a subset for validation based on their known deregulation in glioblastoma and other cancers and their widespread expression in glioblastoma samples and patient-derived glioblastoma stem cells. We checked the expression of these selective targets, CD44, TOP2A, MDM2, RHOC, HMGA2, PLAU, and NUSAP1, in TCGA glioma patient samples and found that they are highly overexpressed in tumor samples compared with Genotype-Tissue Expression (GTEx) normal brain samples (Supplemental Figure 2, A–G). We performed extensive target validation using immunoblot and 3′-UTR reporter analyses. We transfected precursor miR-340 and miR-382 into glioblastoma cell lines A172, U87, and U251, as well as patient-derived glioblastoma stem cell lines GSC-34 and GSC-28. Cell lysates were then subjected to immunoblot. Overexpression of these miRNAs in various glioblastoma cells, and stem cells, led to a marked reduction in protein levels across all tested cell lines (Figure 2, A–L, and Supplemental Figure 3, A–I). To determine if miR-340 and miR-382 directly target CD44, TOP2A, RHOC, MDM2, HMGA2, EGFR, PDGFRA, NUSAP1, and PLAU by binding to their 3′-UTRs, we amplified the 3′-UTR sequences containing the miR-340 and miR-382 binding sites from genomic DNA and subcloned them into the psiCheck2 luciferase reporter plasmid under the control of the T7 promoter. These 3′-UTR luciferase reporter constructs were then transiently transfected into U87 cells along with either a scrambled control miRNA mimic or mimics of miR-340 or miR-382. Overexpression of miR-340 resulted in a significant decrease in luciferase signals for CD44, RHOC, HMGA2, MDM2, EGFR, and PDGFRA (*P* < 0.05). Similarly, miR-382 overexpression led to a significant reduction in 3′-UTR luciferase signals for PLAU, CD44, NUSAP1, and MDM2 (*P* < 0.05), compared with scrambled control transfections (Figure 2, M and N). The predicted miRNA response elements in these targets were identified based on 6-mer seed complementarity from PAR-CLIP analysis. Importantly, mutation of these seed-matching regions within the respective 3′-UTRs abolished this repression, confirming the specificity of miR-340 and miR-382 targeting. These findings provide evidence that miR-340 and miR-382 directly bind to their respective 3′-UTRs and significantly repress the expression of numerous deregulated genes in glioblastoma.

### miR-17 is an oncogenic regulatory miRNA in glioblastoma.

**Figure 3:**
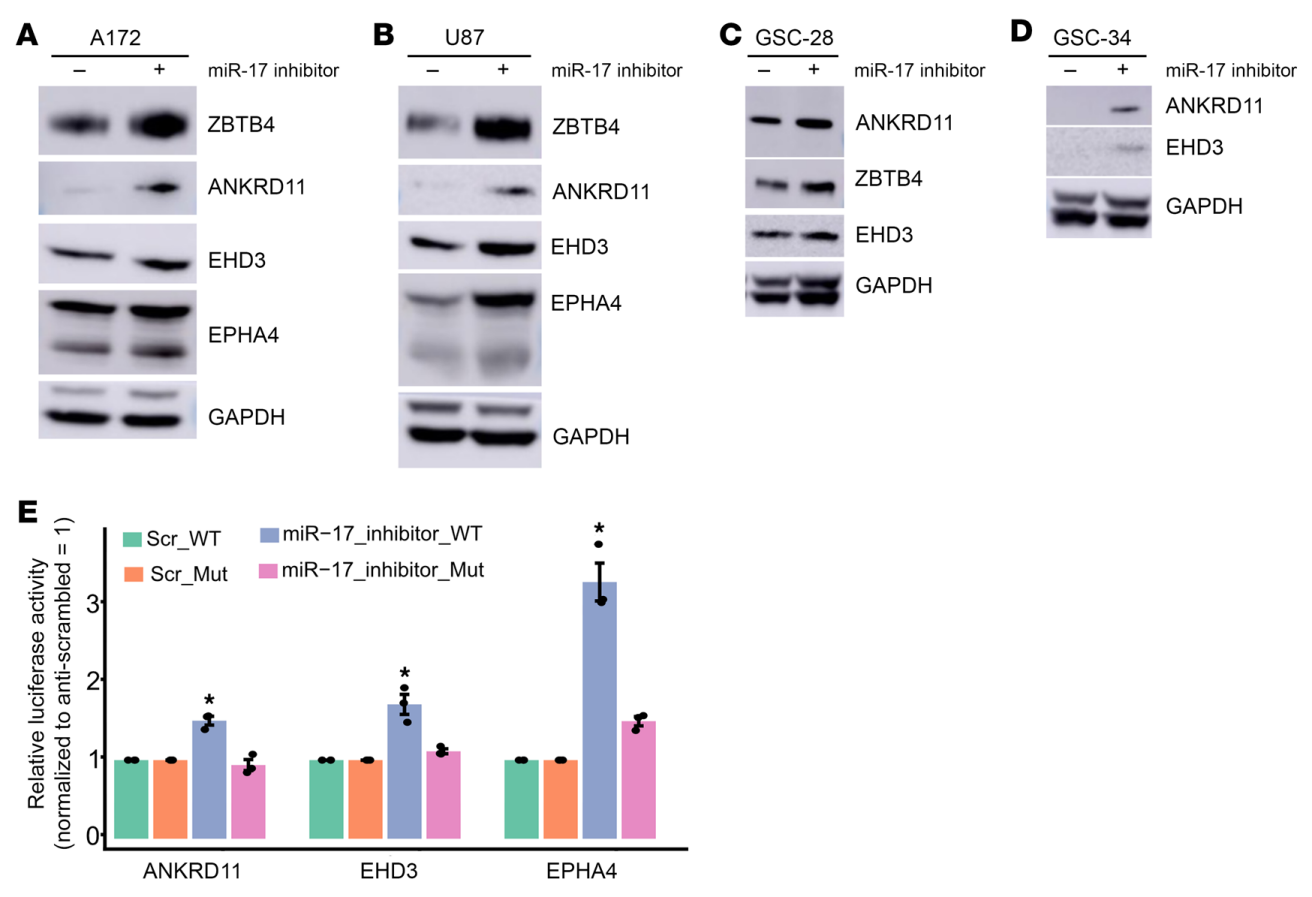
Validation of miR-17 targets in glioblastoma cells and patient-derived stem cells. (**A**–**D**) Glioma cell lines A172, U251, GSC-28, and GSC-34 were transfected with a miRNA inhibitor targeting miR-17. Immunoblots were performed with antibodies against ANKRD11, EHD3, and EPHA4. GAPDH served as the internal control for loading. The data show that the miR-17 inhibitor increased protein expression compared with negative controls. (**E**) U87 cells were cotransfected with miR-17 or miR-17 inhibitor along with psiCheck2 luciferase reporter plasmids containing the wild-type 3′-UTRs of targets ANKRD11, EHD3, EPHA4, and ZBTB4, as well as corresponding mutant 3′-UTR constructs in which the predicted miR-17 binding sites were disrupted. The data show that the miR-17 inhibitor increased luciferase activity for all respective wild-type 3′-UTR targets compared with negative controls, while this effect was abolished or significantly reduced in the mutant constructs. Forty-eight hours after transfection, cells were lysed and luciferase signals were measured. Data represent mean ± SEM from 3 independent experiments. *P < 0.05 by 2-tailed Student’s *t* test.

We identified miR-17 as one of the top oncogenic regulatory miRNAs that targets several deregulated TSGs in glioblastoma. We determined the expression of the selective targets in TCGA glioma samples and found they are highly downregulated in tumor samples compared with GTEx healthy brain samples (Supplemental Figure 4, A–D). We validated some of these targets using immunoblot analysis and 3′-UTR reporter assays in several glioma cell lines and patient-derived GSC lines. Since miR-17 is upregulated in glioblastoma and acts as an oncogene, we inhibited miR-17 by transfecting a miR-17 inhibitor into glioma cell lines (A172, U87, U251) and patient-derived GSC lines (GSC-28 and GSC-34). We selected specific targets — *ZBTB4*, *ANKRD11*, *EHD3*, and *EPHA4* — based on their widespread published roles in glioblastoma. Inhibiting miR-17 resulted in an increased expression of all these targets in both glioblastoma cells and stem cells, indicating that miR-17 suppresses their expression (Figure 3, A–D, and Supplemental Figure 5, A–D). To confirm that miR-17 directly binds to the 3′-UTRs of ANKRD11, EHD3, and EPHA4, we conducted a luciferase reporter assay. The 3′-UTRs of these genes, which contain the miR-17 binding sites, were subcloned into a psiCheck2 reporter plasmid. In addition, mutant constructs harboring point mutations in the predicted miR-17 seed-binding sites were generated. Glioblastoma cells were transfected with either a scrambled anti-miR or an miR-17 inhibitor, along with the luciferase reporter plasmid containing the wild-type or mutant miR-17 target binding sites. Transfection with the miR-17 inhibitor led to a significant derepression (increase) in luciferase signals for ANKRD11, EHD3, and EPHA4 (*P* < 0.05), compared with the scrambled control (Figure 3E), whereas mutation of the binding sites abolished this effect. These data show that miR-17 directly binds to and regulates several deregulated mRNAs in glioblastoma.

Further, we investigated whether the targets of the regulatory miRNAs, miR-340, miR-382, and miR-17, are associated with patient survival. To evaluate survival outcomes, we utilized 2 independent cohorts of primary glioma patient samples from TCGA and the Chinese Glioma Genome Atlas (CGGA) for survival analysis. Kaplan-Meier survival curves were generated and analyzed using the online tools GEPIA and CGGA, where patients were stratified into high– and low–gene expression groups based on target expression levels. For the tumor suppressor targets of miR-340 and miR-382, high expression was correlated with poorer patient survival, while low expression was associated with more favorable outcomes in both TCGA and CGGA cohorts (Supplemental Figure 6, A–G, and Supplemental Figure 7, A–G). Conversely, for the oncogenic miR-17, high expression of its targets was linked to better survival, while low expression was associated with poorer patient prognosis (Supplemental Figure 8, A and B, and Supplemental Figure 9, A and B). Additionally, we performed survival analysis using CGGA data from recurrent glioma patient samples, which revealed similar survival trends for both tumor suppressor and oncogenic miRNA targets (Supplemental Figure 10, A–F, and Supplemental Figure 11). These findings underscore the importance of regulatory miRNAs and their target genes in determining glioma patient outcomes.

### Regulatory miRNAs miR-340 and miR-382 regulate multiple pathways in glioblastoma.

After identifying and validating regulatory miRNAs and their targets, we investigated their functions. We first performed pathway analyses with the targets of miR-340 and miR-382. We performed GO term, KEGG, and Hallmark pathway analysis. The most significant pathways associated with miR-340 were the mitotic cell cycle phase transition, stem cell differentiation, miRNAs in cancer, PI3K-Akt signaling, proteoglycan in cancer, the hallmarks of apoptosis, G2/M checkpoint, and epithelial to mesenchymal transition (Supplemental Figure 12, A–C). The pathways enriched for miR-382 targets were response to hypoxia, signaling pathways associated with miRNA metabolic process, proteoglycan in cancer, miRNAs in cancer, G2/M checkpoint, and epithelial to mesenchymal transition (Supplemental Figure 12, D–F). These data suggest that these miRNAs regulate different cancer-related pathways, and inhibiting them could attenuate glioma growth by inhibiting numerous oncogenic pathways.

### Regulatory miRNAs miR-340 and miR-382 inhibit cell proliferation, invasion, and neurosphere formation in several glioblastoma cell lines.

**Figure 4:**
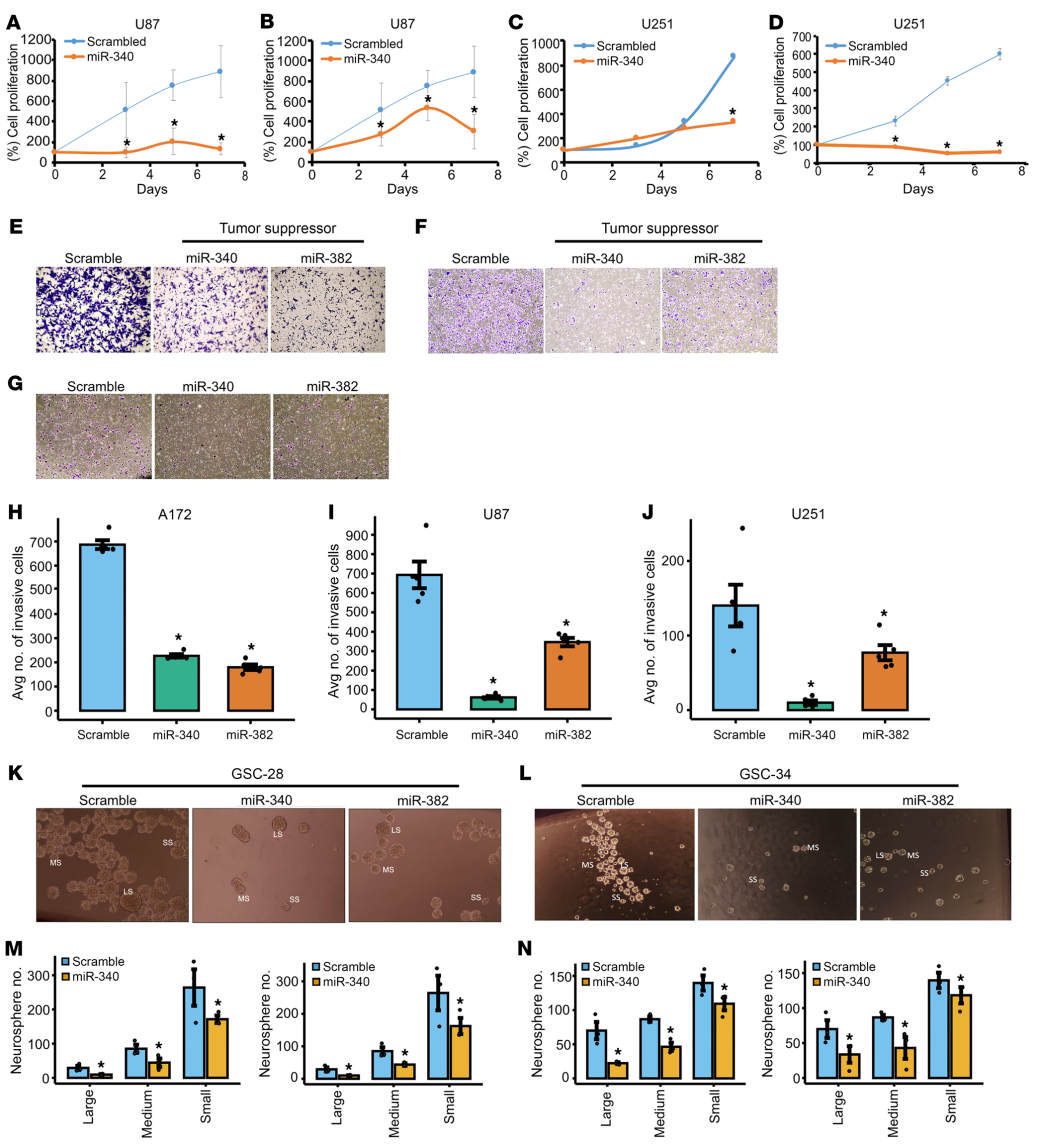
miR-340 and miR-382 inhibit cell proliferation, invasion, and neurosphere formation in glioblastoma. (**A**–**D**) Glioblastoma cell lines U87 and U251 were transfected with a scrambled control, miR-340 precursor, or miR-382 precursor. Cell counts were performed at various time points, and miR-340 and miR-382 showed decreased cell growth compared with negative controls. (**E**–**J**) A172, U87, and U251 cells were transfected with mimic miR-340 and miR-382, and invasion assays were executed. Invaded images from 5–10 random fields were captured and analyzed using ImageJ software (NIH) for quantification. miR-340 and miR-382 decreased invasion compared with negative controls. (**E**–**G**, **K**, and **L**) Original magnification, ×10. (**K**–**N**) Six-well plates were precoated with poly-ornithine, and glioblastoma stem cell lines GSC-28 and GSC-34 were plated and transfected with scrambled control miRNA, miR-340, or miR-382. Neurosphere images were taken from 5 distinct fields 72 hours posttransfection and categorized into large, medium, and small using ImageJ software, with quantifications presented (**M** and **N**). miR-340 and miR-382 decreased neurosphere formation compared with negative controls. LS, large sphere; MS, medium sphere; SS, small sphere. Data represent mean ± SEM from 3 independent experiments. *P < 0.05 by 2-tailed Student’s *t* test.

We then performed a thorough assessment of the functional and experimental therapeutic impacts of miR-340 and miR-382. We first quantified their endogenous expression levels in glioblastoma cell lines, GSCs, and banked human tumors including cell lines (A172, T98G, LN18, U251, U87, SNB19), and stem cell lines (GSC-28, GSC-20, GSC-34, GSC-627, GSC-267), as well as patient glioblastoma samples, using quantitative PCR. Normal human astrocytes and normal human cortex were used as a control. We observed a significant downregulation of miR-340 and miR-382 expression in cell lines compared with normal human astrocytes, mirroring the pattern observed in patient glioblastoma samples compared with normal brain samples (Supplemental Figure 13, A–D). Notably, miR-382 showed upregulation in only a single tumor tissue sample, while all other samples remained consistently downregulated. For miR-340, a majority of the cell lines showed downregulation, although a few cell lines exhibited relatively unchanged expression. These variations are consistent with the well-established intratumoral heterogeneity of glioblastoma. This consistent downregulation hinted at a potential tumor suppressor role for these miRNAs, which was consistent with our analyses (Supplemental Figure 13, A–D). Subsequently, we conducted cell proliferation and invasion assays to determine the biological significance of miR-340 and miR-382 in vitro across different glioblastoma cell lines. Glioblastoma cells were transfected with either scrambled control miRNA or precursor miR-340 or precursor miR-382, and cell proliferation was assessed through trypan blue staining and live-cell counting. Overexpression of precursor miR-340 or miR-382 led to a significant reduction in cell proliferation compared with the scrambled negative control (Figure 4, A–D). To investigate the influence of these miRNAs on glioma cell invasion, we performed Transwell invasion assays using glioblastoma cell lines. The Transwell invasion chambers were precoated with collagen IV, one of the abundant ECM components in the brain. Glioblastoma cell lines were transfected with scrambled control miRNA, or miR-340 or miR-382 precursors, before being plated in the Transwell chambers. These cells were allowed to invade through the collagen IV layer. Transient overexpression of either miR-340 or miR-382 led to a substantial reduction in glioblastoma cell invasion compared with scrambled negative control miRNA-transfected cells (Figure 4, E–J).

Glioblastoma contains self-renewing stem cells contributing to tumor initiation and resistance to therapy (24). To elucidate the effect of the regulatory miRNAs on the self-renewal property of GSCs, we conducted neurosphere assays using 2 patient-derived glioblastoma stem cell lines, GSC-34 and GSC-28. We transfected precursor miR-340 or precursor miR-382 or scrambled controls into the glioblastoma stem cells and counted the number of neurospheres 7 days after miRNA transfection. Neurospheres were categorized based on their size under the microscope (10× original magnification) into large, medium, and small groups. Overexpression of miR-340 and miR-382 resulted in a reduction in the total number of neurospheres in both GSCs. These findings suggest that these miRNAs impair neurosphere formation, potentially reflecting effects on self-renewal capacity (Figure 4, K–N).

### Inhibiting oncogenic miR-17 in glioma cells decreases cell proliferation, invasion, and neurosphere formation in glioblastoma cell lines and GSCs.

**Figure 5:**
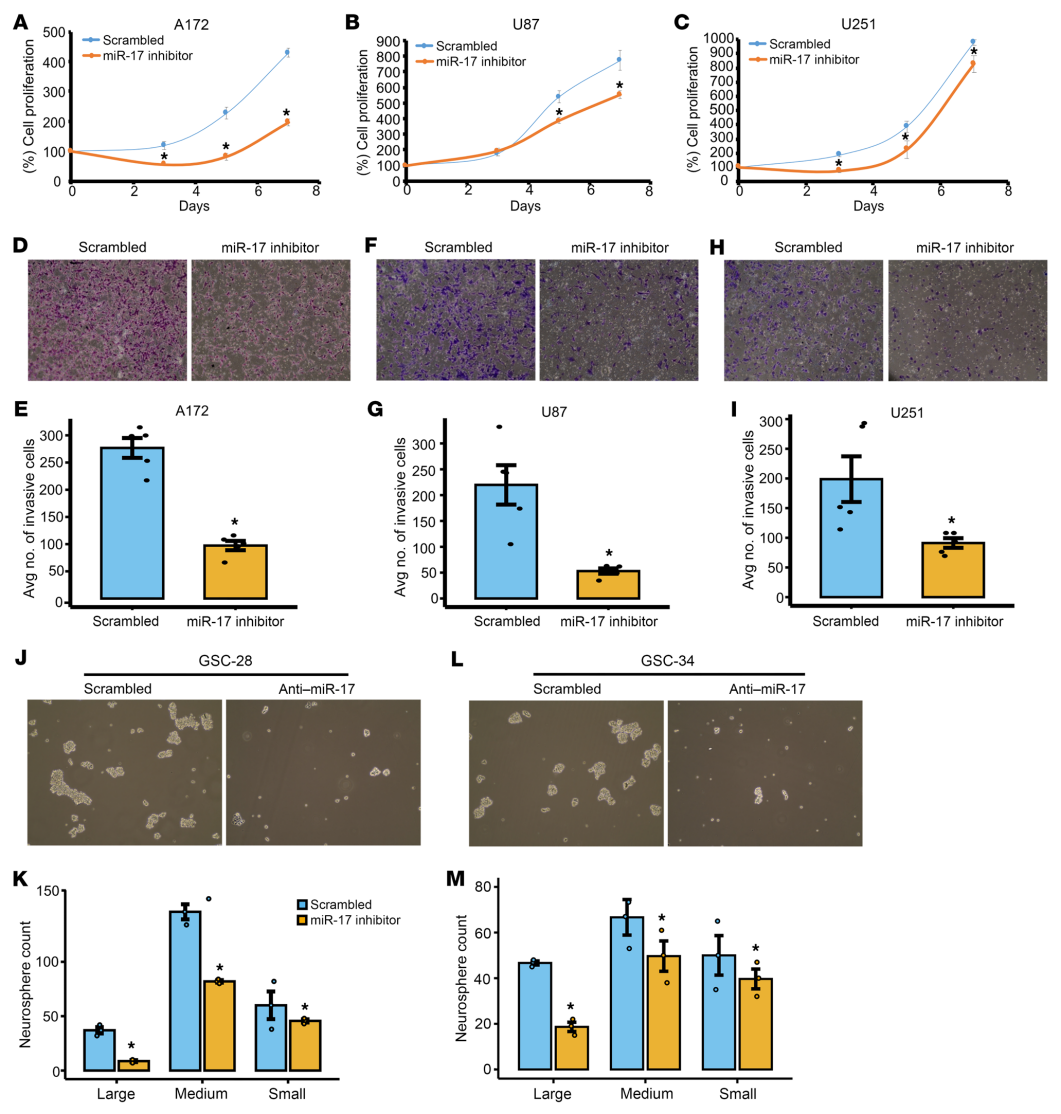
Effects of miR-17 inhibition on cell proliferation, invasion, and neurosphere formation in glioblastoma. (**A**–**C**) A172, U87, and U251 were transfected with either scrambled control miRNA or a miR-17 inhibitor. Cells were counted 48 hours posttransfection using trypan blue exclusion at various intervals to assess viability. (**D**–**I**) A172, U87, and U251 cells were transfected with control or miR-17 inhibitor. Invasion assay was carried out, the invaded cells were stained with crystal violet, and images were captured and analyzed using ImageJ software for quantification. (**D**, **F**, **J**, and **L**) Original magnification, ×10. (**J**–**M**) Glioma stem cell lines GSC-28 and GSC-34, plated on poly-ornithine–coated, 6-well plates, were transfected with either a scrambled control miRNA or an miR-17 inhibitor. Neurospheres were imaged 72 hours posttransfection in neurobasal complete growth medium. Images from 5 microscopic fields were taken, and neurosphere sizes were categorized into large, medium, and small for quantification using ImageJ software. Data are presented as mean ± SEM from 3 independent experiments. *P < 0.05 by 2-tailed Student’s *t* test.

miR-17 is one of the top oncogenic regulatory miRNAs identified by our integrated approach. Similar to the tumor-suppressive regulatory miRNAs, we first checked the endogenous expression of miR-17 in multiple glioma and stem cell lines by RT-qPCR (Supplemental Figure 13, E and F). We observed an increased expression pattern in all glioma and stem cell lines, consistent with our algorithm that identified miR-17 as an oncogenic miRNA. Therefore, we performed a series of functional assays with miR-17 in a spectrum of glioblastoma cell lines, including patient-derived glioblastoma stem cells. For the cell proliferation assay, we transfected the inhibitor against miR-17 into multiple glioma cell lines, then counted the cells on different days. We observed a decrease in cell proliferation, supporting the oncogenic nature of miR-17 in glioma cells (Figure 5, A–C). Transwell cell invasion assays were carried out with miR-17 inhibitor in glioblastoma cell lines. The invasion assays were carried out with a collagen IV–coated Transwell chamber. Inhibition of miR-17 in glioblastoma cell lines led to a decrease in cell invasion through collagen-coated chambers, which were counted by taking images from 5 random microscopic fields and quantified with ImageJ software (NIH) (Figure 5, D–I). The neurosphere formation assay was performed with GSC lines GSC-28 and GSC-34. The transfection of GSC lines with an inhibitor of miR-17 decreases the overall neurosphere formation. We categorized the neurosphere size into 3 different groups, large, medium, and small, based on the size calculator with ImageJ software. The overall neurosphere number in different categories decreased significantly after miR-17 inhibitor treatment compared with the scrambled control treatment (Figure 5, J–M).

### Inhibition of oncogenic miR-17 and overexpression of tumor-suppressive miR-340 and miR-382 reduce in vivo glioblastoma growth.

**Figure 6:**
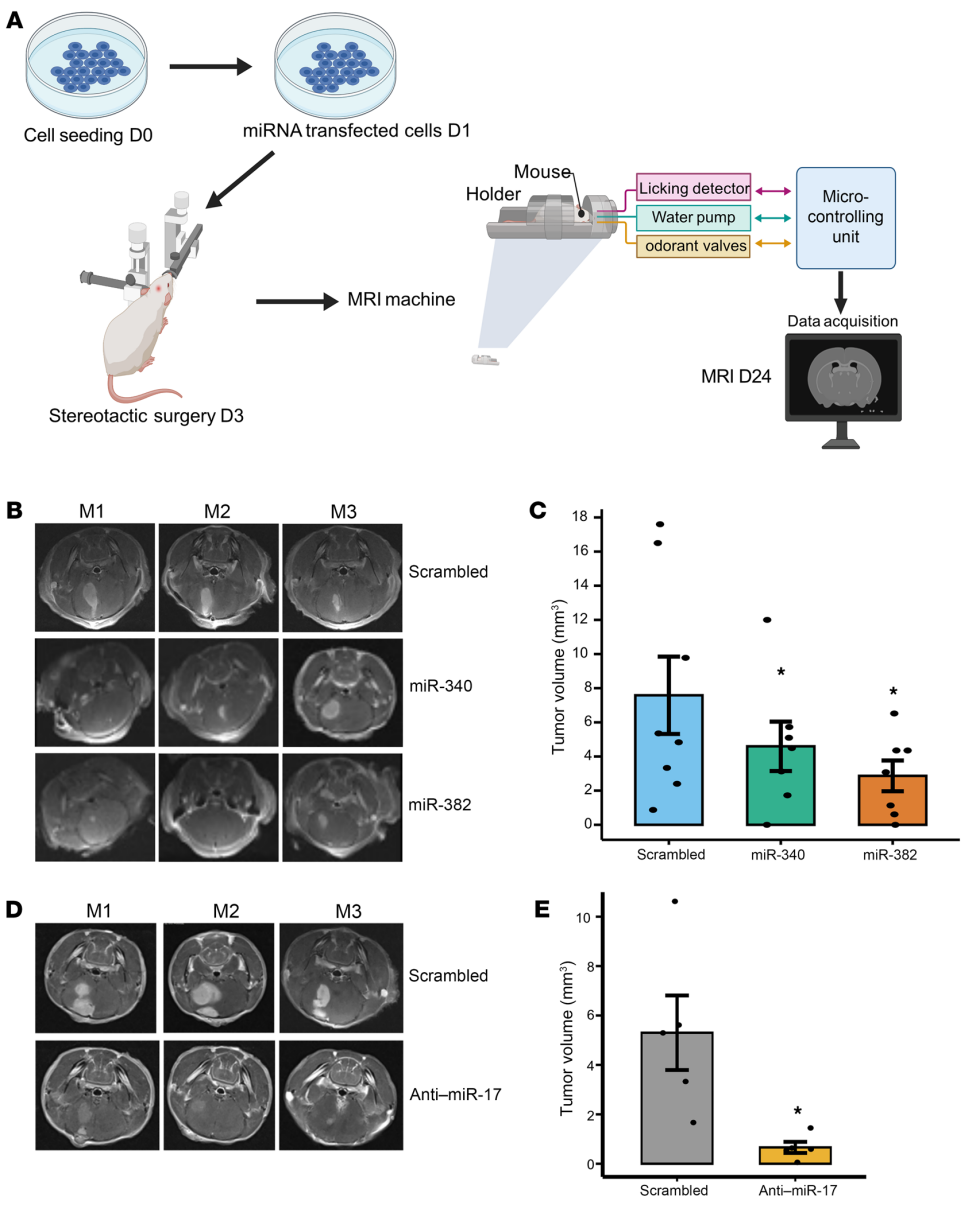
miR-340, miR-382, and miR-17 regulate in vivo tumor growth. (**A**) Schematic overview of the experimental design for tumor implantation and timeline for MRI to assess tumor volume. Each group comprised 7 mice for surgery. (**B**–**E**) U87 cells were transfected with scrambled negative miRNA, miR-340, miR-382, or an inhibitor of miR-17. At 48 hours posttransfection, cells were implanted intracranially into the striata of 5- to 6-week-old immunodeficient mice. Mice were monitored 3–4 weeks, and MRI was performed to evaluate tumor generation. Representative MRIs and quantification showing that miR-340, miR-382, and miR-17 inhibitor showed reduction in tumor volume compared with scrambled controls. *P < 0.05 by 2-tailed Student’s *t* test.

The impact of miR-340 and miR-382 overexpression on xenografted tumor growth in mice was investigated. We transfected 3 × 10^5^ U87 cells with a scrambled negative control miRNA (*n* = 7), precursor miR-340 (*n* = 7), precursor miR-382 (*n* = 7), or miR-17 inhibitor (*n* = 7) (Figure 6A). The transfected cells were then stereotactically implanted into the striata of 6-week-old immunodeficient mice. Over 3 weeks, the mice were closely monitored for tumor growth and survival, and MRI images were obtained to visualize the tumors. After 3 weeks, the scrambled control U87 group exhibited significant tumor growth. In contrast, the groups treated with miR-340 and miR-382 displayed a significant reduction in tumor volume (Figure 6, B and C). Specifically, the scrambled control group reached a tumor volume of 7.58 ± 2.26 mm^3^, while the mice bearing miR-340– or miR-382–transfected tumors showed tumor volumes of 4.59 ± 1.44 mm^3^ and 2.86 ± 0.9 mm^3^, respectively (Figure 6C). The mice implanted with miR-17 inhibitor–transfected cells showed a substantial decrease in tumor volume compared with scrambled control (Figure 6, D and E). The scrambled control mouse group showed tumor volumes of 5.3 ± 1.94 mm^3^, whereas miR-17 inhibitor group exhibited tumor volumes of 0.68 ± 0.29 mm^3^. Collectively, these in vivo findings support the use of miR-340, miR-382, the miR-17 inhibitor, or a combination of them as new therapeutics for glioblastoma.

### Therapeutic delivery of PEG-PBAE BPNs carrying plasmids encoding miR-340 and miR-382 to mice bearing glioblastoma tumors by MRIgFUS inhibits tumor growth.

**Figure 7:**
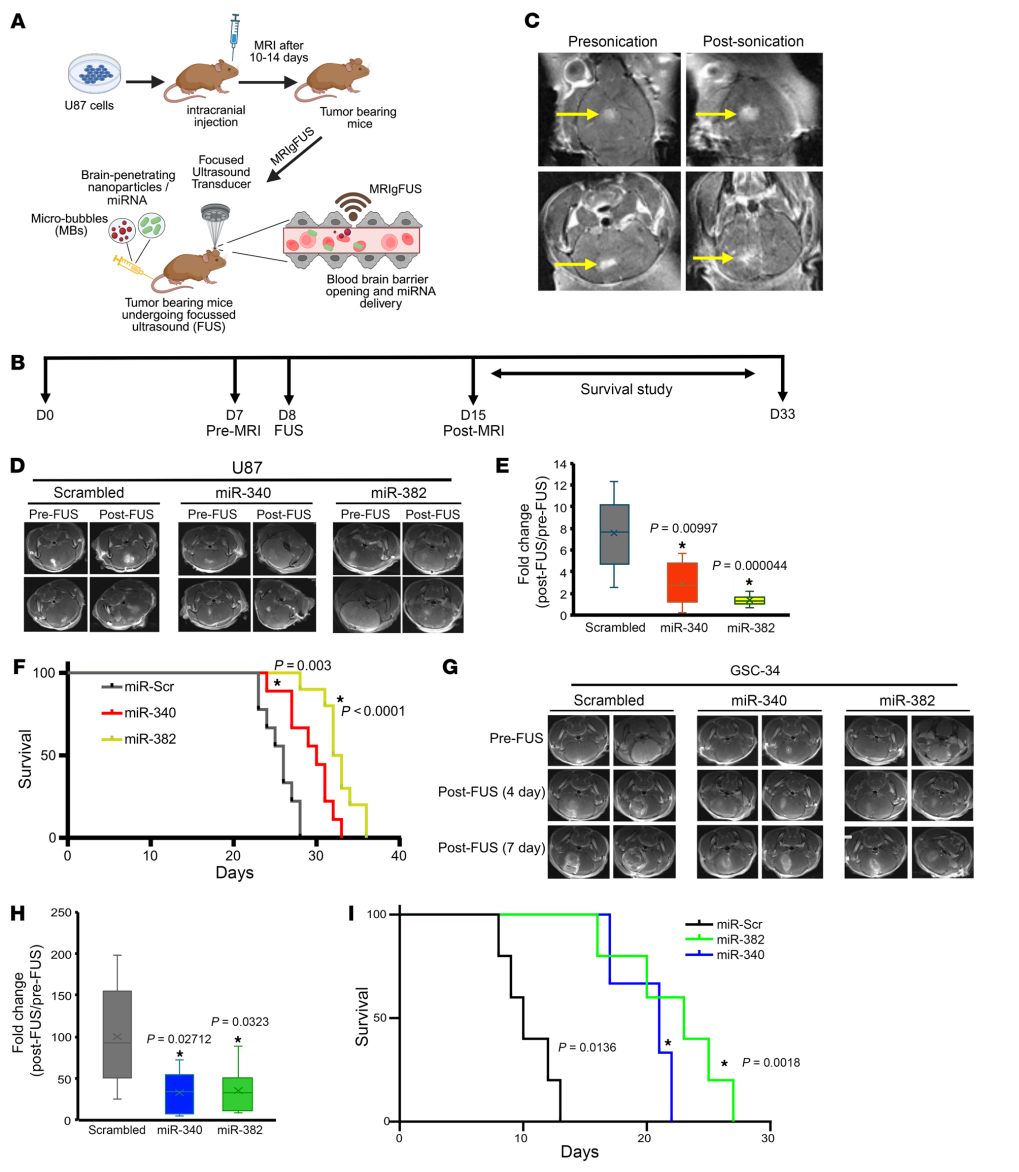
Inhibition of in vivo glioma growth through MRI-guided focused ultrasound, microbubble, and brain-penetrating nanoparticle delivery of miR-340 or miR-382. (**A**) A schematic representation of FUS-MB-BPN–mediated miRNA delivery into mice. (**B**) Overall experimental plan and timeline for FUS-MB-BPN. (**C**) Tumors in mice were sonicated before and after FUS-MB-BPN, and images were captured to validate blood-brain barrier opening. MBs were employed to facilitate blood-brain barrier opening. Arrow indicates the tumor location and show dispersion of the contrast, demonstrating successful opening of the blood-brain barrier. MRI images were acquired using a small-animal MRI system with a slice thickness of 0.7 mm. The acquisition parameters included a repetition time (TR) of 600 ms and an echo time (TE) of 11 ms. (**D** and **E**) MBs were injected through mice’s tail veins, and MRIgFUS was conducted. Upon confirmation of blood-brain barrier opening, BPN conjugated with scrambled, miR-340, or miR-382 were injected through mice’s tail veins. The mice were imaged using MRI, and tumor volume shows significant reduction upon delivery of miR-340 or miR-382 compared with scrambled controls at day 15. Box plots show the interquartile range, median (line), and minimum and maximum (whiskers). (**F**) Kaplan-Meier survival curve showing miR-340 or miR-382 significantly prolonged survival compared with scrambled control mice. (**G**–**I**) For patient-derived glioma stem cell (GSC-34) intracranial xenografts, the same FUS-MB-BPN protocol was applied using miR-340 and miR-382. MRI analysis at day 4 and day 7 post-FUS showed a reduction in tumor volume over time, and Kaplan-Meier survival analysis demonstrated improved survival in miR-340– and miR-382–treated groups compared with scrambled controls. *n* = 7 mice per group. *P < 0.05 by 2-tailed Student’s *t* test for miR-340–treated vs. scrambled control.

For the therapeutic delivery of regulatory miRNA, we developed and employed an approach consisting of MRIgFUS and MBs (FUS-MB) to facilitate the delivery of BPNs carrying miR-340 and miR-382 encoding lentiviral plasmids. This innovative approach (abbreviated FUS-MB-BPN) circumvents the hurdles of miRNA therapeutics, particularly the blood-brain barrier and tumor penetration and transfection, and delivers cargo loads to tumor cells. The schematic plan for FUS-MB-BPN is depicted in Figure 7, A and B. We generated glioblastoma xenografts in immunodeficient mice, accomplished by intracranial implantation of 3 × 10^5^ U87 cells into the striata of mice brains. Once the tumors had formed (7–10 days postinjection), we verified tumor formation by MRI and the safe and effective opening of the blood-brain barrier. This step was achieved through a meticulously orchestrated combination of microbubbles and sonication, resulting in a conspicuous increase in MRI signal intensity surrounding the tumors. This increased signal intensity is attributed to the leakage of the MRI contrast agent, gadobenate dimeglumine, into the brain parenchyma after blood-brain barrier opening (Figure 7C). To induce robust miRNA expression in brain tumors, BPNs were formulated with lentiviral plasmids encoding miR-340 or miR-382. We also prepared the BPNs with plasmids encoding scrambled miRNA sequences as a control group. We extensively characterized these BPNs because the ζ-potential and size of the particles are important parameters for effective blood-brain barrier penetration. The hydrodynamic diameter and ζ-potential of BPNs carrying plasmids encoding scrambled miRNA, miR-340, and miR-382 were measured as 70.2 ± 4.7 nm, 2.8 ± 0.8 mV; 71.2 ± 1.2 nm 2.3 ± 0.6 mV; and 70.7 ± 1.0 nm, 1.8 ± 0.7 mV, respectively (Supplemental Table 8). After tumor implantation, we divided the brain tumor–bearing mice into 2 cohorts receiving plasmids encoding scrambled miRNA or tumor-suppressive miR-340 and miR-382. Each group consisted of 7 mice. The MRIgFUS-MB-BPN procedure was then executed. The BPNs carrying plasmids encoding scrambled miRNA, miR-340, or miR-382 were intravenously injected alongside MBs, and FUS was applied to the tumor regions to transiently open the blood-brain barrier and facilitate the delivery of the plasmid cargoes into the tumor. One week after the FUS-MB-BPN procedure, MRI images were taken to visualize and measure the tumor volumes. Delivery of miR-340 or miR-382 by FUS-MB-BPN procedure into the tumors significantly reduced tumor burden compared with the control group (*n* = 7 mice per group, *P* < 0.05) (Figure 7, D and E). This represents to our best knowledge the first successful FUS-MB-BPN-procedure–based experimental therapeutic delivery of a miRNA to inhibit tumor growth. We also closely monitored the post–FUS-treated mice for survival, which revealed that delivery of miR-340 or miR-382 into brain tumors significantly extended the survival of these mice compared with those treated with the scrambled control miRNA, with miR-382 providing greater survival benefit (Figure 7F). To determine whether the therapeutic efficacy of FUS-MB-BPN–mediated miRNA delivery was restricted to U87 xenografts, we next extended our analysis to additional models. First, intracranial xenografts were established using patient-derived GSCs (GSC-34) and treated with the same FUS-MB-BPN protocol. Delivery of the tumor-suppressive miRNAs miR-340 or miR-382 significantly reduced tumor burden and improved survival relative to the scrambled control group (Figure 7, G–I). To further evaluate treatment kinetics, longitudinal MRI was performed on days 4 and 7 post-FUS, revealing a progressive decline in tumor volume over time and thereby indicating sustained therapeutic efficacy (Supplemental Figure 14, A–D). Importantly, delivery of BPNs carrying miR-340 or miR-382 in the absence of FUS did not produce a substantial reduction in tumor burden (FUS group: scrambled, miR-340, and miR-382: 64.81 ± 10.38, 15.65 ± 3.19, and 22.31 ± 9.99 mm^3^, respectively; without FUS: 62.67 ± 11.97, 77.89 ± 14.67, and 72.09 ± 3.60 mm^3^), highlighting the necessity of FUS-mediated blood-brain barrier opening for effective tumor delivery (Figure 7, G–I, and Supplemental Figure 15, A–H). Collectively, these findings demonstrate that FUS-MB-BPN–mediated delivery is broadly applicable across multiple glioblastoma models and effective for therapeutic miRNA delivery. To assess potential toxicity from FUS-MB, we harvested various organs including the liver, kidney, brain, heart, spleen, lung, lymph nodes, and pancreas from treated mice. We then performed H&E staining on these organs, which were analyzed by a neuropathologist. The data showed no apparent damage to the liver, kidney, brain, or heart in treated mice compared to the nontreated controls (Supplemental Figure 16, A and B). These data demonstrate that FUS-MB-BPN is a safe and effective strategy for delivering miRNAs into brain tumors.

### In vivo target repression and durability following MRIgFUS-mediated delivery of miR-340 and miR-382 to GSC-34 xenografts.

**Figure 8:**
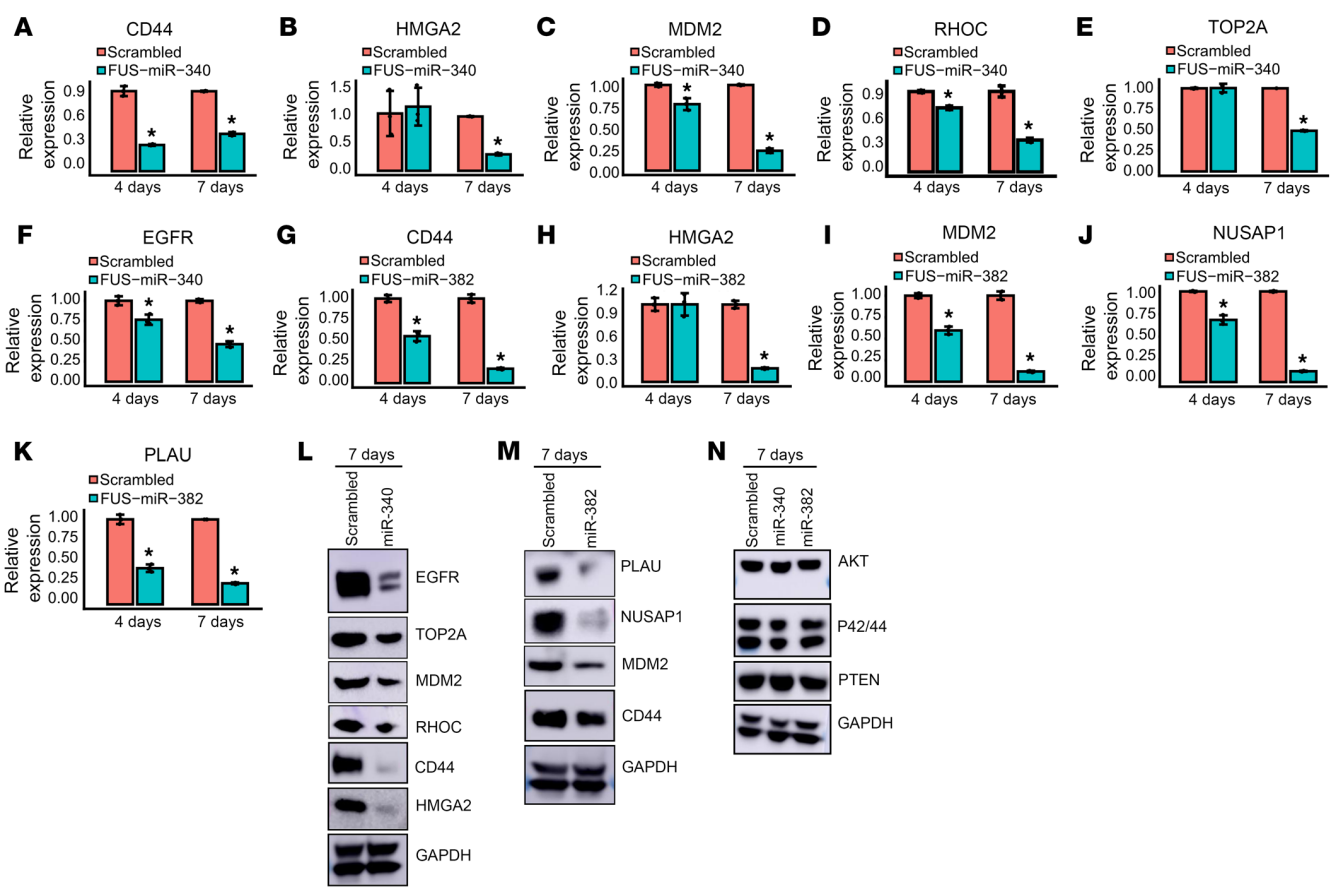
In vivo target repression and durability following MRIgFUS-mediated delivery of miR-340 and miR-382 in GSC-34 glioma xenografts. (**A**–**K**) Quantitative PCR analysis of validated target transcripts demonstrates significant downregulation in tumors treated with miR-340 or miR-382 compared with scrambled controls at day 4, with more uniform and sustained repression observed at day 7. While some targets exhibited modest suppression at day 4, consistent transcript-level repression across all examined targets was evident by day 7, indicating progressive and durable in vivo silencing following FUS-mediated delivery. (**L** and **M**) Western blot analysis of tumor lysates at day 7 confirms reduced protein expression of validated on-target genes in miR-340– and miR-382–treated tumors relative to controls. (**N**) Expression of selected nontarget proteins remains unchanged, supporting specificity of miRNA-mediated repression. Statistical significance was determined using a 2-tailed Student’s *t* test. *P < 0.05.

To determine whether the therapeutic effects observed following FUS-MB-BPN–mediated delivery of miR-340 and miR-382 were associated with effective and sustained repression of their molecular targets in vivo, we performed studies using GSC-34 glioma xenografts. Intracranial tumors were generated by implantation of GSC-34 cells into nude mice. After confirmation of tumor establishment by MRI, mice underwent MRIgFUS in combination with MBs to transiently open the blood-brain barrier, followed by intravenous administration of BPNs carrying plasmids encoding miR-340, miR-382, or scrambled control miRNA. Tumor tissues were harvested at 4 and 7 days after FUS treatment for molecular analyses. Quantitative PCR analysis revealed significant downregulation of validated target transcripts for both miR-340 and miR-382 at 4 days posttreatment compared with scrambled controls (Figure 8, A–K). While several targets showed robust repression at this early time point, a subset demonstrated more modest changes at day 4. Notably, by day 7, repression was consistently observed across all examined targets, indicating progressive and sustained transcript-level suppression following FUS-mediated miRNA delivery (Figure 8, A–K). To determine whether transcript repression translated into functional protein downregulation, we analyzed protein expression in tumor lysates derived from the same GSC-34 samples. Western blot analysis at day 7 demonstrated marked reduction of validated on-target proteins in tumors treated with miR-340 or miR-382 relative to scrambled controls (Figure 8, L and M). In contrast, expression levels of selected off-target proteins remained unchanged, supporting the specificity of miRNA-mediated repression in vivo (Figure 8N). Collectively, these data demonstrate that FUS-MB-BPN delivery of miR-340 and miR-382 into GSC-34 brain tumors induces specific and durable suppression of molecular targets at both the mRNA and protein levels. The progressive repression observed between days 4 and 7 further supports sustained biological activity of the delivered miRNAs in vivo and provides molecular evidence underlying the observed antitumor effects.

## Discussion

Glioblastoma is a lethal and aggressive primary brain tumor. Despite extensive research and clinical trials, targeted therapies have faced insurmountable challenges presented by the blood-brain barrier, tumor invasiveness, resistance to cytotoxic therapies, and tumor heterogeneity (25). One major hurdle toward the success of targeted therapies for glioblastoma is the concurrent deregulation of numerous genes in any single tumor (14, 15). The simultaneous targeting of several deregulated oncogenic drivers using conventional drugs is severely limited by the fact that the drugs needed to simultaneously target many molecules do not currently exist, and because combining several drugs in a clinical setting leads to an exponential increase in toxicity (20). To overcome these limitations, we developed and successfully tested a new miRNA therapeutic strategy.

miRNAs are small noncoding RNAs that can be deregulated in cancers and brain tumors, where they can function as either oncogenes or tumor suppressors (26). Because miRNAs do not require full complementarity with the targeted mRNA sequences to inhibit gene expression, a single miRNA can target multiple genes and inhibit their expression (27). We therefore reasoned that there exist regulatory miRNAs, which can inhibit multiple deregulated genes in glioblastoma, and that these miRNAs can be used as therapeutic agents/targets, equivalent to using multiple drugs in combination (28). Combination drug therapies are difficult because many deregulated molecules do not have drugs that target them and because combining several drugs can lead to exponential increase in toxicity (29, 30). Argonaute proteins AGO1–4 are core components of the RNA-induced silencing complex, but they differ functionally. AGO2 uniquely possesses endonucleolytic (“slicer”) activity, while AGO1 and AGO3 primarily mediate translational repression and mRNA destabilization (31). In contrast, AGO4 is generally expressed at lower levels in most somatic tissues and has a less well-defined role in canonical miRNA-mediated silencing (32). Because AGO1–3 represent the predominant mediators of miRNA-guided repression in glioma cells, our PAR-CLIP analysis focused on these proteins to capture the major functional miRNA–mRNA interactions in this context. However, as these PAR-CLIP experiments were performed in U87 cells, the identified miRNA–mRNA interactions may not fully reflect the heterogeneity across glioblastoma subtypes. Future studies across additional patient-derived models will be important to establish the generalizability of these findings. To find regulatory miRNAs, we developed an approach that integrates miRNA target identification with PAR-CLIP with TCGA data analyses to find miRNAs that target multiple dysregulated important genes in glioblastoma. The initial PAR-CLIP experiments in U87 cells identified potential miRNA–target interactions, but these do not necessarily indicate the direction of miRNA dysregulation in patient tumors. Subsequent integration with TCGA data allows identification of miRNAs that are downregulated in glioma, reflecting the clinical context rather than the cell line expression. This unique approach was invented and developed in our lab. It can be applied to any human cancer.

Our approach uncovered numerous miRNAs that had numerous deregulated targets in glioblastoma. We focused on the highly ranked tumor-suppressive miR-340 and miR-382 and the oncogenic miR-17. We validated a few of their targets, TOP2A, RHOC, CD44, HMGA2, and MDM2 for miR-340 (Figure 2, A–G); CD44, NUSAP1, PLAU, and HMGA2 for miR-382 targets (Figure 2, H–L); and ZBTB4, ANKRD11, EHD3, and EPHA4 (Figure 3, A–D) for miR-17. These target genes were selected for validation based on prior evidence from our glioblastoma models, consistent expression across the cell lines used in this study, and the availability of antibodies for Western blot analysis. Given the well-established heterogeneity of glioblastoma, not all targets were uniformly expressed across all models; therefore, we focused on representative and biologically relevant targets. Importantly, as regulatory miRNAs act by coordinately regulating multiple genes within oncogenic networks, their functional effects are driven by cumulative pathway modulation rather than dependence on any single target (33–40). These selected targets were validated across multiple cell lines, including patient-derived glioma stem cell lines, using immunoblotting and 3′-UTR reporter assays. CD44 is a cell surface adhesion protein highly expressed in many cancers, including cancer stem cells, and regulates cancer progression and metastasis (41). TOP2A has been identified as an oncogene in multiple cancers (34, 42). TOP2A is overexpressed in pan-cancers, and overexpression is correlated with poor prognosis and advanced pathological stages in most cancers (42). RhoC is a member of the RhoGTPase family protein, which has been shown to be involved in cancer cell migration, invasion, and metastasis (43). All selected targets are deregulated in glioblastoma, where they play important roles in regulating malignancy. More targets were identified and validated but are not all discussed in this section due to space limitations.

We then functionally validated the regulatory miRNAs by performing growth, death, invasion, and differentiation assays as well as in vivo tumor growth assays. These assays validated the tumor-suppressive (miR-340 and miR-382) or oncogenic (miR-17 potential) effects of the selected regulatory miRNAs. We showed that the impact of these miRNAs was pervasive and profound as illustrated in Figure 4, A–F. While these functional assays support the tumor-suppressive or oncogenic roles of the selected miRNAs, we acknowledge that neurosphere formation assays were used to assess self-renewal capacity. Although informative, neurosphere assays are less quantitative than extreme limiting dilution assays, which are considered the gold standard for estimating stem cell frequency. We recognize that reduced sphere formation may also be influenced by changes in proliferation rate, which cannot be fully distinguished in this assay system. In addition, we cannot exclude a contribution of altered cell viability to the observed effects. Given their ability to orchestrate concerted regulation of multiple pathways, we aptly designated miR-340, miR-382, and miR-17 as “regulatory miRNAs” and potential therapeutic agents or targets for glioblastoma therapy.

To translate these findings into therapy, we designed and tested an approach for the therapeutic delivery of miRNAs to glioblastoma animal models. We prepared BPNs carrying lentiviral plasmids encoding either miR-340 or miR-282 or scrambled controls, then employed FUS-MB-BPN to deliver this therapeutic payload to mice harboring glioblastoma. This approach led to a significant reduction in tumor growth and prolongation of animal survival. The mice did not exhibit any signs of toxicity. This new noninvasive delivery method represents a pivotal step toward effective and targeted miRNA therapeutics. Efficient drug delivery into brain tumors is impeded by the blood-brain barrier. Utilizing MRIgFUS combined with MBs provides a noninvasive technique to temporarily open the blood-brain barrier for delivering therapeutic molecules with BPNs at the tumor site, offering both temporal and spatial control (44–46). MRIgFUS combined with MBs has been utilized in multiple studies to deliver therapeutic molecules into brain tumor (47). The blood-brain barrier inhibits the majority of chemotherapeutic drugs’ delivery into the brain, including the chemotherapeutic drug doxorubicin (48). The polymeric nanoparticles have many advantages over cationic polymers such as polyethylenamine and poly-l-lysine nanoparticles because they have more stability in the bloodstream, superior blood-brain barrier–crossing potential, increased drug solubility, and more drug encapsulated load and controlled drug release, which make them more suitable for delivering therapeutics when combined with MRIgFUS. Among the promising polymers, PBAEs offer a library of nontoxic, biodegradable materials for the compaction of nucleic acids (49). In our study we used surface-modified PEGylated PBAE nanoparticles, which can penetrate brain parenchyma and open the blood-brain barrier efficiently when combined with MBs and FUS (49, 50). These blood-brain barrier–penetrating nanoparticles are more diffusive in nature and were able to circulate in the brain at least for 24 hours, suggesting they have low adhesive interactions with ECM that could lead to the clearance or low distribution of these particles in the brain parenchyma. The precision of FUS transducers enables them to focus on a millimeter scale, accurately targeting only the tumor region.

Recent advances in FUS-mediated blood-brain barrier disruption have rapidly transitioned from preclinical proof of concept toward clinical application in human glioblastoma. FUS, when paired with intravenously administered MBs, enables safe, targeted, and reversible blood-brain barrier opening that enhances delivery of chemotherapeutics, immunotherapies, and biologics to otherwise inaccessible tumor and peritumoral regions. Early clinical studies using MRIgFUS systems have demonstrated that repeated blood-brain barrier disruption is feasible and well tolerated in patients with recurrent glioblastoma, with evidence of increased intratumoral drug penetration and favorable safety profiles (51, 52). Moreover, ongoing trials are assessing FUS-mediated delivery of standard agents such as temozolomide and new immune modulators, highlighting its potential to augment therapeutic efficacy. These developments position FUS as a versatile platform for improving central nervous system drug delivery and underscore the importance of integrating such technologies into translational frameworks for glioblastoma treatment.

In conclusion, our study developed and successfully tested approaches to identify and deliver therapeutic regulatory miRNAs to glioblastoma. They can be translated into clinical trials using the available clinical-grade FUS-MB-BPN at our and other institutions. The approaches can also be easily adapted for use in other cancers.

## Methods

### Sex as a biological variable.

Only male (or only female) mice were used in this study. This choice was made to reduce variability associated with sex-dependent hormonal differences and to improve experimental consistency in tumor growth models. Findings are expected to be generalizable to both sexes, although sex-specific effects were not directly evaluated.

### Computational and experimental workflow for identifying putative regulatory miRNAs.

Our new methodology for identifying key regulatory miRNAs and their prioritization involved several steps. First, all miRNA–mRNA targets were identified through PAR-CLIP (described in more detail in the Supplemental Methods). Argonaute/target gene complexes were collected, the argonaute protein was digested, and the complexed target genes were then sequenced. T/C alignment analysis was used to identify genes with the distinctive mutation that identifies genes that complex with miRNAs. The sequence target fragments were then compared with a list of known miRNA seed sequences using string sequencing matching in R. Specifically, miRNAs that targeted genes in the 3′-UTR were prioritized, while genes with no miRNA matches or matches in the CDSs or 5′-UTRs were filtered out. Next, we analyzed 166 glioblastoma multiforme RNA-seq datasets from TCGA, which were normalized against 255 normal brain datasets from the GTEx (*n* = 255) project using the bowtie and bedtools genomic sequencing alignment packages. For 42,644 genes, these tumor samples were compared with normal using the DESeq2 package in R, revealing genes that were greater than 2-fold up- and downregulated. Prior to downstream analyses, we implemented prefiltering measures, discarding genes with read counts of fewer than 5 reads. In the third step, the CancerMine database was employed to classify each miRNA target gene as an ONC, a TSG, or neither, using experimentally verified labels from published manuscripts. Using this method, the final curated repository contained 1,849 published oncogenes and 1,478 published tumor suppressors, a 1.25:1 ratio. The fourth step involved performing a survival analysis on the significantly deregulated genes from the previous differential expression analysis. Using the R programming package, survminer, a Cox proportional hazard ratio was determined for each putative target gene. The fifth step involved filtering out targets that did not have a “consistent” and “significant” survival and deregulation trend. For oncogenic targets, these parameters were determined as a minimum 2-fold increase in expression and a multiple-hypothesis-adjusted *P* ≤ 0.05, coupled with either an inconclusive correlation with survival (Cox coefficient between –0.2 and 0.2) or a correlation with poor prognosis (Cox coefficient > 0.2). Tumor-suppressive targets required a minimum 2-fold decrease in expression, an adjusted *P* ≤ 0.05, and either an inconclusive correlation with survival or a correlation with good prognosis (Cox coefficient < –0.2). Targets that met these criteria were labeled “consistent” and were retained for the next step. For this sixth step, the retained “consistent” targets were assigned a composite score based on the percentile ranks of their absolute expression and differential expression scores. For example, a target at the 100th percentile would score 1, and at the 0th percentile would score 0, with the highest possible score being 2 for targets at the 100th percentile for both absolute and differential expression. Subsequently, each miRNA received 2 scores reflecting the composite scores of their annotated targets: a TSG score from oncogenic targets and an ONC score from tumor-suppressive targets. These scores were then ranked by percentile. In the final analysis, the percentile rank difference between the TSG and ONC scores for each miRNA determined its classification. The ONC percentile rank was subtracted from the TSG rank. A difference exceeding 25% classified a miRNA as a TSG miRNA (TSG > ONC), while a difference below –25% classified it as an ONC miRNA (ONC > TSG). This threshold was derived from the 1.25:1 oncogene enrichment in our CancerMine dataset described in step 3 of this analysis. For instance, miR-1185, with a TSG score in the 84th percentile and an ONC score in the 12th percentile, was identified as a projected tumor suppressor miRNA (+72% difference). This approach also minimized the emphasis on miRNAs targeting numerous ONC and TSG genes, as they would rank highly in both categories. For example, miR-4709 ranked in the 100th percentile for TSG and 90th percentile for ONC but would receive “inconclusive” classification by our algorithm, as there is only a 10% score difference in these roles.

### PAR-CLIP.

A total of 1 μg of FLAG-tagged AGO1, AGO2, and AGO3 plasmids was transfected into 2 × 10^5^ U87 cells via Lipofectamine 2000 (Invitrogen) transfection. Following transfection, the cells were subjected to puromycin selection at a concentration of 1 μg/mL for 48 hours to generate stable cell lines. Verification of AGO1, AGO2, and AGO3 overexpression was conducted through immunoblot analysis employing AGO1, AGO2, and AGO3 antibodies. The PAR-CLIP methodology was implemented and adapted from the previously established protocol (20). Briefly, AGO1, AGO2, and AGO3 stable cell lines were cultured overnight in the presence of 100 μM ^4S^U, to label all cellular nascent RNA. Subsequently, these ^4S^U-labeled cells were exposed to 365 nm UV light (utilizing a Thomas Scientific Spectrolinker XL-1500) to effectuate the cross-linking of labeled nascent RNA with RNA binding proteins. Following UV exposure, cells were rinsed twice with 1× PBS, harvested, and lysed using a buffer composed of 50 mM HEPES-KOH (pH 7.5), 150 mM KCl, 2 mM EDTA (pH 8.0), 1 mM NaF, 0.5% NP-40, 0.5 mM DTT, and freshly prepared protease inhibitor cocktails (catalog P8340, Sigma-Aldrich and RNAse T1, catalog ENO541, ThermoFisher Scientific). Cell lysates were centrifuged at 15,000*g* for 15 minutes at 4°C. The supernatant from the lysed cells was subjected to treatment with RNase T1 at a concentration of 1 U/μL for 15 minutes at room temperature. Subsequently, the FLAG-tagged AGO1, AGO2, AGO3 cell lysates’ supernatant underwent IP, facilitated by an anti-FLAG antibody conjugated to Protein G Dynabeads (Thermo Fisher Scientific). The immunoprecipitated material was further subjected to digestion with RNase T1, and the resulting beads were subjected to washing with a high-salt wash buffer. The washed beads were resuspended in a dephosphorylation buffer and treated with calf intestinal alkaline phosphatase for 10 minutes at 37°C to eliminate phosphate groups from RNA molecules. Next, the dephosphorylated beads were treated with polynucleotide kinase and radioactive [γ-32P]-ATP for 30 minutes at 37°C to introduce RNA labeling. The protein-RNA complexes were then separated via SDS-PAGE and subsequently electroeluted. These complexes exhibited a migration pattern at around 100 kDa. The electroeluted samples then underwent digestion with proteinase K to release RNA from the complexes, followed by RNA extraction involving an acid phenol/chloroform mixture and ethanol precipitation. The extracted RNA was then converted into cDNA, and adaptor ligation was executed following previously established protocols (31). The resulting libraries were sequenced; the generated short reads were mapped against the human genome hg38, mRNA, and miRNA precursor databases; and the clustered sequences were identified. The PAR-CLIP clustered sequences were identified by T-to-C conversion at the ^4S^U cross-linked site. The majority of the clustered sequences were found at the 3′-UTRs. Finally, the true target sites were determined from the clustered sequences based on the list of input miRNA seed sequences.

### DNA-BPN synthesis and characterization.

The preparation of BPN formulation was conducted in accordance with the protocol outlined in a previous publication (53). In summary, PBAE and PEG-PBAE were synthesized through the conjugation of 1,11-diamino-3,6,9-trioxaundecane (obtained from Millipore Sigma) or 5 kDa methoxy-PEG-*N*-hydrosuccinimide (from Sigma-Aldrich) with the acrylate groups on PBAE (sourced from Sigma-Aldrich), respectively. The scrambled control miRNA (catalog PMIRH000PA-1), miR-340 (catalog PMIRH340PA-1), and miR-382 (catalog PMIRH382PA-1) lentiviral plasmids were purchased from System Biosciences. All constructs are based on the pCDH-CMV-MCS-EF1-copGFP backbone, into which the respective precursor miRNA sequences were subcloned according to the manufacturer’s design. A mixture of PBAE and PEG-PBAE polymers was then prepared at a 3:2 ratio by PBAE amount to create a highly PEGylated surface. For the formulation of DNA-BPN, polymers and nucleic acids were vigorously mixed at a weight ratio of 60 and a volume ratio of 1:5. This mixture was allowed to incubate at room temperature for 30 minutes to facilitate nanoparticle assembly. Subsequently, the solution was placed into 100 kDa MWCO Amicon Ultra Centrifugal Filters (MilliporeSigma) and centrifuged at 1,000*g* for 15 minutes at 4°C. To remove residual polymers from the DNA-BPN solution, the concentrated DNA-BPNs, with a nucleic acid concentration adjusted to 1 mg/mL, were diluted 10-fold with DNase/RNase-Free Distilled Water and recentrifuged under the same conditions. After undergoing 2 additional washing steps, the final DNA-BPN solution, with a nucleic acid concentration of 1 mg/mL, was prepared for subsequent in vivo experiments. Physicochemical properties of BPNs are described in Supplemental Table 8.

### MRIgFUS.

To establish tumors, immunodeficient mice aged 6–8 weeks were utilized. Specifically, 3 × 10^5^ U87 cells (ATCC) or GSC-34 patient-derived stem cells were intracranially introduced into the striata of immunodeficient mice. Jeongwu Lee, Cleveland Clinic (Cleveland, Ohio, USA), and Erik P. Sulman (New York, New York, USA), provided the glioblastoma stem cell lines GSC-34 and GSC-28 was from MD Anderson Cancer Center, Houston, Texas, USA. After a 3-week injection period, MRIgFUS was used following a well-established protocol, with some modifications (44). Briefly, the MRIgFUS experimental setup featured an MRI-compatible, prefocused, 8-element phased array, accompanied by a 1.5 MHz geometrically focused transducer boasting a 25 mm active diameter and a focal ratio of 0.8. These components were interconnected through a phased array generator and a radiofrequency power amplifier. It was connected to an MRI-compatible motorized stage to precisely control the transducer’s movements in the rostral-caudal and medial-lateral orientations. Degassed water was introduced into the spherical transducer’s membrane to ensure effective coupling between the membrane and the mice’s brains. At the same time, acoustic gel was applied to both the inflated membrane and the shaved portion of the mice’s skull. These measures prevented the entrapment of air bubbles. For intravenous injections, a catheter was inserted into the mouse’s tail. MBs (25 μL/kg body weight) and an MRI contrast agent, gadobenate dimeglumine (0.1 mL), were administered via this catheter with saline. Subsequently, a series of MRI images were captured. The precise positioning of the mouse’s brain relative to the transducer was determined by locating the transducer’s position within the MRI space. During sonication and MRI imaging, the mice were positioned in a prone posture. The region of interest encompassing the tumor was defined, and sonication was carried out using a 1.5 MHz transducer to induce the opening of the blood-brain barrier around the tumor. MRI images were acquired using a surface coil incorporated into the FUS system. The effectiveness of blood-brain barrier opening was verified by comparing MRI sections before and after sonication. This MRIgFUS approach provided a powerful method for noninvasive modulation of the blood-brain barrier, enabling targeted delivery of therapeutic agents to brain tumors in preclinical models.

Detailed descriptions of all other methods can be found in the Supplemental Methods.

### Statistics.

All data are represented as mean ± SEM from 3 independent biological replicates. The *P* value is calculated by 2-tailed Student’s *t* tests when comparing 2 groups. *P* values < 0.05 were considered statistically significant.

### Study approval.

Animal experiments were approved by the University of Virginia (Charlottesville, Virginia, USA) Animal Care and Use Committee (ACUC) (Protocol No. 3542-05-21). All animal procedures, including handling, monitoring, housing, and experimental interventions, were conducted in accordance with the NIH *Guide to the Care and Use of Laboratory Animals* (National Academies Press, 201 and institutional ACUC regulations. Human glioma cell lines were used for intracranial implantation in mouse models. The use of human cell lines was approved by the Institutional Review Board of the University of Virginia.

### Data availability.

The PAR-CLIP dataset generated in this study has been deposited in the Gene Expression Omnibus under accession number GSE293517. All the data values are reported in the Supporting Data Values file (https://github.com/ss7st/miRNA_PARCLIP_analysis.git). All codes and materials supporting the findings of this study are available from the corresponding author upon reasonable request.

## Author contributions

SS, YZ, MKG, CD and R Abounader conceptualized the study. SS, YZ, MKG, CD, FH, EQXM, SB, YS, PM, XW, GK, AP, HL, KH, TS, MM, FG, NC, RRC, WL, SC, PK, ALK, JSS and JM developed the methodology. SS, YZ, MKG, CD and R Abounader conducted the investigation. SS, YZ, CD, MKG, FH, PM, MD and KH contributed to visualization. R Abounader acquired the funding, administered the project, and supervised the study. SS and R Abounader wrote the original draft. SS, YZ, MKG, CD, FH, EQXM, SB, YS, PM, XW, GK, AP, HL, KH, MD, TS, MM, FG, NC, RRC, LDO, R Anbu, WL, SC, BK, PK, ALK, DS, JSS, JH, JM, MH and R Abounader reviewed and edited the manuscript.

## Conflict of interest

The authors have declared that no conflict of interest exists.

## Funding support

This work is the result of NIH funding, in whole or in part, and is subject to the NIH Public Access Policy. Through acceptance of this federal funding, the NIH has been given a right to make the work publicly available in PubMed Central.

- NIH/NCI U01 CA220841, NIH/NINDS R01 NS122222, NIH/NINDS R21 NS122136, NIH/NCI Cancer Center Support Grant P30 CA044579.
- University of Virginia Comprehensive Cancer Center Pilot Grant.
- Schiff Foundation Grant.
- Ben and Catherine Ivy Foundation Grant.
- Focused Ultrasound Foundation Grant.

## Supplementary Material

Supplemental data

Unedited blot and gel images

Supplemental table 1

Supplemental table 2

Supplemental table 3

Supplemental table 4

Supplemental table 5

Supplemental table 6

Supplemental table 7

Supporting data values

## Version 1 06/30/2026

In-Press Preview

## Version 2 08/17/2026

Electronic publication
