# Supplementary material for: Therapeutic delivery of microRNAs discovered to target deregulated glioblastoma pathways inhibits tumor growth in mice
Source: J Clin Invest. 2026 Jun 30;136(16):e195639. doi: 10.1172/JCI195639 (PMC13476142; doi:10.1172/JCI195639)
Supplement: Supplementary file 1 [file jci-136-195639-s009.pdf]

Supplemental methods

Cell culture and transfection

All glioblastoma cell lines used in this study were procured from the American Type Culture Collection (ATCC). Specifically, U87 cells were cultured and maintained in Minimal Essential Medium (MEM) supplemented with one mM sodium pyruvate, one mM non-essential amino acids (NEAA), 10 ml of 7.5% sodium bicarbonate, 1% penicillin/streptomycin, and 10% fetal bovine serum. A172 and 293T cells were cultured in DMEM high glucose medium supplemented with 1% penicillin/streptomycin and 10% fetal bovine serum. Glioblastoma stem cells, GSC-34, we from Cleveland Clinic, and GSC-28 were obtained from MD Anderson Cancer Center. These stem cells were cultured following a previously published protocol (*25*). The culture medium for stem cells consisted of 2.5 ml of N-2 (0.5X), 5 ml of B27 (0.5X), 50 ng/ml EGF, 50 ng/ml FGF, 0.5 mM glutamine, and 1% penicillin/streptomycin. Regular maintenance included dissociation using an accutase solution when the neurospheres reached a specific size. The primary astrocytes were from Lonza Bisoscience and were cultured in growth medium kit from Lonza Bioscience. All cells were cultured at 37°C in a humidified incubator with a 5% CO2 atmosphere to maintain cell viability. Both glioblastoma and GSCs were transiently transfected with either 30 nM of a scrambled negative control miRNA, target-specific mimic primary miRNA (ThermoFisher, hsa-miR-340, Catalog # AM17100, hsa-miR-382, Catalog # AM17100), or 30-50 nM of a miRNA inhibitor(ThermoFisher, hsa-miR-17 Inhibitor Catalog # 4464084), using the Lipofectamine RNAiMax reagent (ThermoFisher). Following transfection, the culture

medium was replaced with a complete growth medium after 6-8 hours. Subsequently, cells were harvested 48 hours post-transfection for various analyses, including RNA isolation, cDNA synthesis, qPCR, and cell lysate preparation for immunoblot analysis to validate miRNA targets.

3'UTR reporter assay

We conducted luciferase reporter assays to investigate miRNA interactions with specific target genes according to the previously published literature (*25*). First, we PCR amplified the 3'-UTRs (3' untranslated regions) targeted by miR-340, miR-382 and miR-17 from genomic DNA. These amplified sequences were then cloned into the psiCheck2 vector, which had been digested with BamH1 and Not1 restriction enzymes. The primers used for amplifying genomic DNA are shown in **Supplementary Table 1**. All reporter plasmids were subjected to Sanger sequencing and verification to ensure sequence accuracy. for the reporter assay, we co-transfected the primary miRNAs along with 1 μg of the psiCheck2 reporter plasmid into our cells of interest. After 48 hours of transfection, we lysed the cells using 1X passive lysis buffer. Subsequently, we performed a luciferase reporter assay using a Promega dual luciferase reporter assay kit. Firefly luciferase activity served as an internal control to normalize the luciferase signal, allowing us to assess the interaction of miRNAs on the regulation of specific target genes.

Invasion assay

To evaluate the effects of miRNAs on cellular invasion, we followed a previously published protocol (*66*). In brief, transwell invasion chambers were coated with 75 μl of a

250 µg/ml collagen IV solution. After 48 hours of miRNA transfection, cells were counted, and 1 x 10^5 cells were re-suspended in 300 µl of 1% serum-containing, antibiotic-free medium. The cells were then added to each transwell chamber, with 750 µl of fetal bovine serum-containing medium in the bottom chamber, creating a gradient that stimulates cell invasion through the collagen IV-coated membrane. Following 6-8 hours of incubation, the invaded cells were stained with crystal violet, and five random fields were captured and counted using ImageJ software.

Gel electrophoresis and immunoblot analysis

Immunoblot assay was performed according to previously published protocols (*67*). After 48 hours of miRNA transfection, cells were harvested and cell lysis was performed using RIPA buffer (50 mM Tris-HCl pH 8.0, 150 mM NaCl, 5 mM EDTA, 0.1% SDS, and 1X protease and phosphatase inhibitor). The protein concentration in the lysates was determined using the Bradford method. Subsequently, 40-100 µg of cell lysates were loaded onto each lane, separated via 4-12% SDS-PAGE, and transferred to a nitrocellulose membrane. To prevent non-specific binding, the membrane was blocked with 5% skim milk and then incubated overnight at 4^0C with primary antibodies targeting specific proteins, including CD44 (1:1000, Catalog#37259,Cell Signaling Technology), TOP2A(1:500, Catalog#sc-3659, Santa Cruz Biotechnology), ANKRD11(1:1000, Catalog# sc-81049, Santa Cruz Biotechnology), ZBTB4(1:1000, Catalog#sc-514883, Santa Cruz Biotechnology), EPHA4(1:1000, Catalog#sc-365503, Santa Cruz Biotechnology), EHD3(1:1000, Catalog#sc-100723, Santa Cruz Biotechnology), GAPDH(1:2000, Catalog#sc-166545, Santa Cruz Biotechnology), MDM2(1:1000,

Catalog#OP115, Sigma Aldrich), RHOC(1:1000, Catalog# 10632-1-AP, Thermo Fisher Scientific), HMGA2(1:1000, Catalog#8179, Cell Signaling Technology), PLAU(1:1000, Catalog# 17968-1-AP, Thermo Fisher Scientific), NUSAP1(1:1000, Catalog#H00051203-B01P). After primary antibody incubation, the membrane was probed with horseradish peroxidase-conjugated secondary antibodies (goat anti-mouse or rabbit) at room temperature for 1 hour. Detection was achieved using a pico-sensitive ECL reagent (Thermo Scientific), and images were captured using an Amersham 600 imager.

Cell proliferation assay

$2X10^4$-$5X10^4$ U87 and U251 cells were seeded and transfected with 30 nM of either scrambled negative control miRNA or primary mimic miR-340, miR-382, or miR-17 inhibitor. After a transfection period of 48 hours, the cells were trypsinized and counted at various time points for seven days. The resulting cell counts were then used to generate growth curves.

RNA isolation, cDNA synthesis, and qPCR

Total RNA was extracted from primary astrocytes, U87, A172, U251, GSC-28 and GSC-34 cells using the Qiagen miRNeasy kit following the manufacturer's protocol. GBM tissue samples were homogenized, and total RNA was isolated using the same kit. Subsequently, 100 ng to 1 µg of total RNA was employed for cDNA synthesis utilizing either the miRscript or miRCURY LNA RT kit. The miRCURY LNA SYBR Green PCR kit was used to assess the expression levels of mature miRNAs. The miRNA fold change

was calculated using the delta-delta Ct method, with primary astrocytes used as a control sample to normalize the fold change.

Neurosphere assay

The neurosphere assay was conducted according to a previously published protocol (*66*). Briefly a 6-well plate was initially coated with a poly-ornithine solution and incubated at 37^0C in a tissue culture incubator for one hour. Afterward, the plates were washed twice with 1X PBS. Glioblastoma stem cells GSC-34 and GSC-28 were dissociated and seeded onto the coated plate. Transfection with the scrambled negative control and the primary mimic miR-340, miR-382, and inhibitor against miR-17 was conducted using the Lipofectamine RNAiMax reagent. After 48 hours of transfection, glioblastoma stem cells were dissociated using an accutase solution and cultured in a neurobasal growth medium for another seven days. The number of neurospheres was quantified both manually and also with ImageJ software based on their size classification (large, medium, and small) and represented in a bar graph.

In vivo effects of miRNA expression

To assess the impact of miRNA overexpression or inhibition in vivo, a mouse xenograft model was employed. Six-week-old athymic nude mice were obtained from the Jackson Laboratory. U87 cells ($3X10^5$) were seeded in a 6-well plate and transfected with 30 nM of either scrambled negative control miRNA or precursor mir-340, miR-382, or mir-17 inhibitor. After 48 hours of transfection, the cells were trypsinized, counted, and utilized for intracranial injection. All mouse experiments were conducted following the University

of Virginia Institutional guidelines. The transfected cell ($3X10^5$) were intracranially injected and stereotactically implanted into the striata of immunodeficient mice, according to a previously established protocol (*66, 67*). After three weeks, MRI imaging was used to visualize and quantify tumor growth. To enhance tumor signal intensity, gadopentetate dimeglumine was intraperitoneally injected. Imaging was conducted using T1-weighted coronal sections with a field size of 2.5 mm × 2.5 mm and a resolution of 256×256 pixels. Osirix lite software was used to calculate tumor volume using MRI tumor images.

Microbubbles preparation

Gas microbubbles were produced in-house using a method previously described (*68*) involving sonication of decafluorobutane gas. The process begins with distearoyl phosphatidylcholine (Lipoid, Germany, or Avanti Polar Lipids, Alabama) and PEG150 monostearate (obtained from Stepan Kessco, Illinois) being dispersed in normal saline at a concentration of 2 mg/ml for each component. This mixture is then sonicated at 20 KHz and 30% power using a Q700 Sonicator (Qsonica, Newton, CT) for 3 minutes to ensure the formation of a submicron micellar dispersion.

To introduce decafluorobutane into the mixture, the headspace of a vial was filled with the gas via a teflon capillary tubing that sparged the gas through the aqueous medium. Sonication was then continued at maximum power for an additional 30 seconds to disperse the gas into the medium and form microbubbles. Following sonication, the sample was allowed to cool and then floated at normal gravity to remove any bubbles larger than 5 µm, in accordance with the guidelines similar to those in {Klibanov, 2004

#104}. The prepared microbubbles were then dispensed into individual vials under a decafluorobutane atmosphere and the vials were stoppered and stored under refrigeration, ensuring they were not frozen. These gas microbubbles are characterized by their neutral nature.

Supplementary Figure Legends

Figure S1: Identification of miRNA targets through PAR-CLIP in glioblastoma cells: A) U87 cells stably expressing Flag-tagged AGO1, AGO2, AGO3 were immunoprecipitated with Flag antibody and immunoblotted with Flag antibody to detect Ago1, Ago2, and Ago3. Supernatant and input were examined to assess pull-down efficiency. The bands at the labeled AGO position show successful pulldown of Ago1, Ago2 and Ago3 respectfully. B) Radiolabeled RNA fragments purified from AGO1, AGO2, AGO3-RNA immuno-complexes were separated on a 15% polyacrylamide gel, and regions of 19-35 bp were excised and purified for 3' and 5' adaptor ligation reactions. C, D) 3' (C) and 5' (D) adaptor ligation reactions were conducted as described in the materials and methods section which shown successful incorporation of adaptors. E) The ligated RNA fragments were reverse transcribed, and libraries were generated for AGO1, AGO2, AGO3 samples. The libraries from AGO1, AGO2, AGO3 were subjected to amplification at different cycles to prevent saturation. F) Heatmaps displaying all the miRNA targets identified in U87 cells by AGO1, AGO2, AGO3 through PAR-CLIP which show that each miRNA exhibits numerous targets across the different AGOs. G-I) Venn diagram showing the distribution of identified targets in 5'-UTR (G), Coding (H) and Intronic (I) regions from AGO1, AGO2 and AGO3 PAR-CLIP

Figure S2: Expression of miR-340 and miR-382 targets in TCGA glioma and GTEx normal brain samples. A–G) Expression levels of validated miR-340 and miR-382 targets — *TOP2A*, *CD44*, *RHOC*, *MDM2*, *HMGA2*, *NUSAP1*, and *PLAU* — were analyzed using the GEPIA online tool in TCGA glioma and GTEx normal brain samples. All targets were significantly upregulated in tumor samples (red bars) compared to normal samples (gray bars). *=$P < 0.05$.

Figure S3: Quantification of target repression and validation of direct miRNA binding by 3′UTR luciferase assays with seed-site mutations. (A–I) Quantification of immunoblot analyses following transfection of glioblastoma cell lines A172, U87, U251, and patient-derived glioma stem cell lines GSC-28 and GSC-34 with scrambled negative control, miR-340, or miR-382. Protein levels of TOP2A (A), RHOC (B), CD44 (C), HMGA2 (D), MDM2 (E), CD44 (F), NUSAP1 (G), PLAU (H), and HMGA2 (I) were normalized to GAPDH and quantified by densitometry. Bar graphs represent relative protein expression compared to scrambled control, demonstrating significant downregulation of target proteins following miR-340 or miR-382 transfection. (J,K) Quantification of 3′UTR luciferase reporter assays in U87 cells co-transfected with miR-340 (J) or miR-382 (K) and psiCheck2 constructs containing either wild-type or seed-sequence–mutated 3′UTRs of the indicated targets. For miR-340, targets included CD44, RHOC, HMGA2, MDM2, EGFR, and PDGFRA. For miR-382, targets included PLAU, CD44, NUSAP1, and MDM2. miR-340 and miR-382 significantly reduced luciferase activity of wild-type 3′UTR

constructs, whereas mutation of the predicted miRNA binding sites abrogated this repression, confirming direct targeting. Data are presented as mean ± SEM from independent experiments.

Figure S4: Expression of miR-17 targets in TCGA glioma and GTEx normal brain samples. A–D) Expression analysis of miR-17 targets — *ZBTB4*, *ANKRD11*, *EHD3*, and *EPHA4* — using GEPIA shows significant downregulation in glioma samples relative to normal brain tissues. *=$P < 0.05$.

Figure S5: Quantification of miR-17–mediated target regulation and validation of direct binding by seed-sequence mutation. A–D) Densitometric quantification of immunoblots shown in Figure 3 following transfection of A172, U251, GSC-28, and GSC-34 cells with a miR-17 inhibitor. Protein levels of ZBTB4, ANKRD11, EHD3, and EPHA4 were normalized to GAPDH. Bar graphs represent relative protein expression compared to negative control, confirming significant upregulation of target proteins upon miR-17 inhibition. E,F) Luciferase reporter assays in U87 cells co-transfected with miR-17 or anti-miR-17 and psiCheck2 constructs containing either wild-type or seed-sequence–mutated 3′UTRs of ANKRD11, EHD3 and EPHA4. Inhibition of miR-17 increased luciferase activity of wild-type 3′UTR reporters, whereas mutation of the predicted miR-17 binding sites abolished this effect, confirming direct and sequence-specific targeting. Data are presented as mean ± SEM from independent experiments.

Figure S6: Kaplan–Meier survival analysis of tumor-suppressive miR-340 and miR-382 targets in TCGA primary glioma patients. A–G) Survival analysis was conducted using the GEPIA tool based on TCGA glioma cohorts. Patients were stratified into high and low expression groups for each target gene. High expression of the target gene (red line) was significantly associated with poor prognosis ($P < 0.05$).

Figure S7: Kaplan–Meier survival analysis of miR-340 and miR-382 targets in CGGA primary glioma patients. A–G) Using the CGGA glioma dataset, high expression of the validated tumor-suppressive targets of miR-340 and miR-382 (red line) was significantly associated with poor prognosis ($P < 0.05$).

Figure S8: Kaplan–Meier survival analysis of oncogenic miR-17 targets in TCGA primary glioma patients. A,B) Survival analysis of selected miR-17 targets in TCGA glioma cohorts. High expression of the target gene (red line) was significantly associated with better prognosis ($P < 0.05$).

Figure S9: Kaplan–Meier survival analysis of oncogenic miR-17 targets in CGGA primary glioma patients. A,B) Kaplan–Meier analysis of selected miR-17 targets in

CGGA glioma samples showed that high expression (red line) was associated with improved survival (*P* < 0.05).

Figure S10: Kaplan–Meier survival analysis of miR-340 and miR-382 targets in CGGA primary glioma recurrent patients. (A–G**)** High expression of validated tumor-suppressive targets of miR-340 and miR-382 (red lines) was significantly associated with poor prognosis in the CGGA recurrent glioma dataset (*P* < 0.05).

Figure S11. Kaplan–Meier survival analysis of the oncogenic miR-17 target EHD3 in recurrent glioma patients from the CGGA primary glioma dataset. Patients were stratified into high and low EHD3 expression groups using the CGGA online analysis tool. High EHD3 expression was significantly associated with improved overall survival in recurrent glioma (*P* < 0.05).

Figure S12: miR-340 and miR-382 regulate cancer pathways: A-F) Targets identified for miR-340 and miR-382 through PAR-CLIP and The Cancer Genome Atlas (TCGA), were utilized as input for pathway analysis involving Gene Ontology (GO) terms for Biological Processes (BP), Kyoto Encyclopedia of Genes and Genomes (KEGG), and gene set enrichment analysis. The significant pathways are highlighted by red dots.

Figure S13: Endogenous expression of miR-340, miR-382, and miR-17 in different glioblastoma cell lines and tissues: A,B) Total RNA was extracted from various glioblastoma tissue samples, followed by cDNA synthesis using the Qiagen miScript RT kit. One microgram of RNA was utilized for cDNA synthesis. The cDNA samples were diluted, and RT-qPCR was conducted with miRNA primers against miR-340 (A), miR-382 (B). Normal brain samples (NB) were used as controls, and U6 was employed as an internal control for normalization. MiR-340 and miR-382 showed decreased expression compared to normal brain samples. C,D) Different glioblastoma cell lines, including A172, T98G, LN18, U251, U87, SNB19, and patient-derived glioblastoma cell lines (GSC-28, GSC-20, GSC-34, GSC-627, GSC-267) were cultured, and cells were harvested for total RNA isolation using the Qiagen miRNeasy kit. Normal human astrocytes (NHA) were used as normal controls. One microgram of total RNA was used for cDNA synthesis with the miRCURY kit, followed by qPCR with primers against miR-340 (C), miR-382 (D). MiR-340 and miR-382 showed decreased expression compared to normal human astrocytes. E) Total RNA was extracted from different glioblastoma tissue samples followed by cDNA synthesis with miScript RT kit using miR-17 primer set. MiR-17 showed elevated expression in all the glioblastoma tissue samples. F) Expression of miR-17 in various glioma cell lines and patient derived glioma stem cell lines by qPCR. miR-17 showed elevated expressed compared to normal human astrocytes. Results are from three independent experiments. * =P < 0.05.

Figure S14: FUS-MB-BPN Delivery of miR-340 and miR-382 reduces the growth of GSC-derived xenografts (a longitudinal analysis): A-D) In patient-derived glioma stem

cell (GSC-34) intracranial xenografts, the MRI-guided focused ultrasound with microbubbles (MRIgFUS-MB)–assisted BPN delivery protocol was applied using Scrambled (A), mir-340(B) and mir-382(C). MRI analysis performed at day 4 and day 7 post-FUS demonstrated a progressive reduction in tumor volume over time in treated groups (D). Statistical significance was determined using a two-tailed Student's t-test (*=P < 0.05).

Figure S15: BPN-Conjugated miRNAs without FUS show no significant impact on Tumor Growth: A-F) To assess the effect of BPN-conjugated miRNAs in the absence of FUS, BPN particles carrying either scrambled miRNA (A, B), miR-340 (C, D), or miR-382 (E, F) were intravenously injected via the tail vein into tumor-bearing mice. Tumor volumes were monitored by MRI at day 4 and day 7 post-injection. No significant changes in tumor volume were observed in mice treated with miR-340- or miR-382-conjugated BPNs compared to the scrambled miRNA control group. G) Tumor fold-change analysis further demonstrated no significant reduction in tumor growth in either the miR-340- or miR-382-treated groups relative to scrambled controls. H) Kaplan–Meier survival analysis showed that treatment with miR-340 or miR-382 delivered via BPNs alone did not result in a significant improvement in overall survival compared to the scrambled miRNA control group.

Figure S16: Magnetic Resonance-guided Focused Ultrasound (MRgFUS)–microbubbles and brain penetrating nanoparticle (FUS-MB-BPN) mediated delivery of miRNA into mouse brain tumors does not induce toxicity. A) Panel A illustrates the delivery of BPN-conjugated miR-142-3p into the mouse brain using MRgFUS, followed by harvesting of various organs under both FUS and non-FUS conditions, with subsequent H&E staining. B) Panel B presents a table summarizing toxicity assessments by an experienced neuropathologist across different organs Liver, Kidney, Brain, Heart, Spleen, Lung, Lymph nodes, Pancreas which showed there was no apparent off-target toxicity to the organs.

Table S1: AGO1–3 PAR-CLIP Binding Sites Overlapping miRNA Seed Sequences and annotated Target Transcripts.

Table S2: Tumor Suppressor and Oncogenic Scores of miRNAs Derived from Integrated AGO1–3 PAR-CLIP Target Analysis.

Table S3: Integrated AGO1–3 Scoring of Oncogenic and Tumor Suppressor miRNAs with Consolidated Unique Target Genes.

Table S4: Integrated AGO1–3 Cumulative Scoring and Consolidated Target Analysis of Tumor Suppressor miRNAs and Corresponding Top 10 Hits.

Table S5: Integrated AGO1–3 Cumulative Scoring and Consolidated Target Analysis of Oncogenic miRNAs and Corresponding Top 10 Hits.

Table S6: Summary of Tumor Suppressive miRNAs and Their Oncogenic Target Scores

Table S7: Summary of oncogenic miRNAs and Their Tumor Suppressive Target Scores

Table S8: Physicochemical properties of PEG-PBAE nanoparticles.

Figure S1

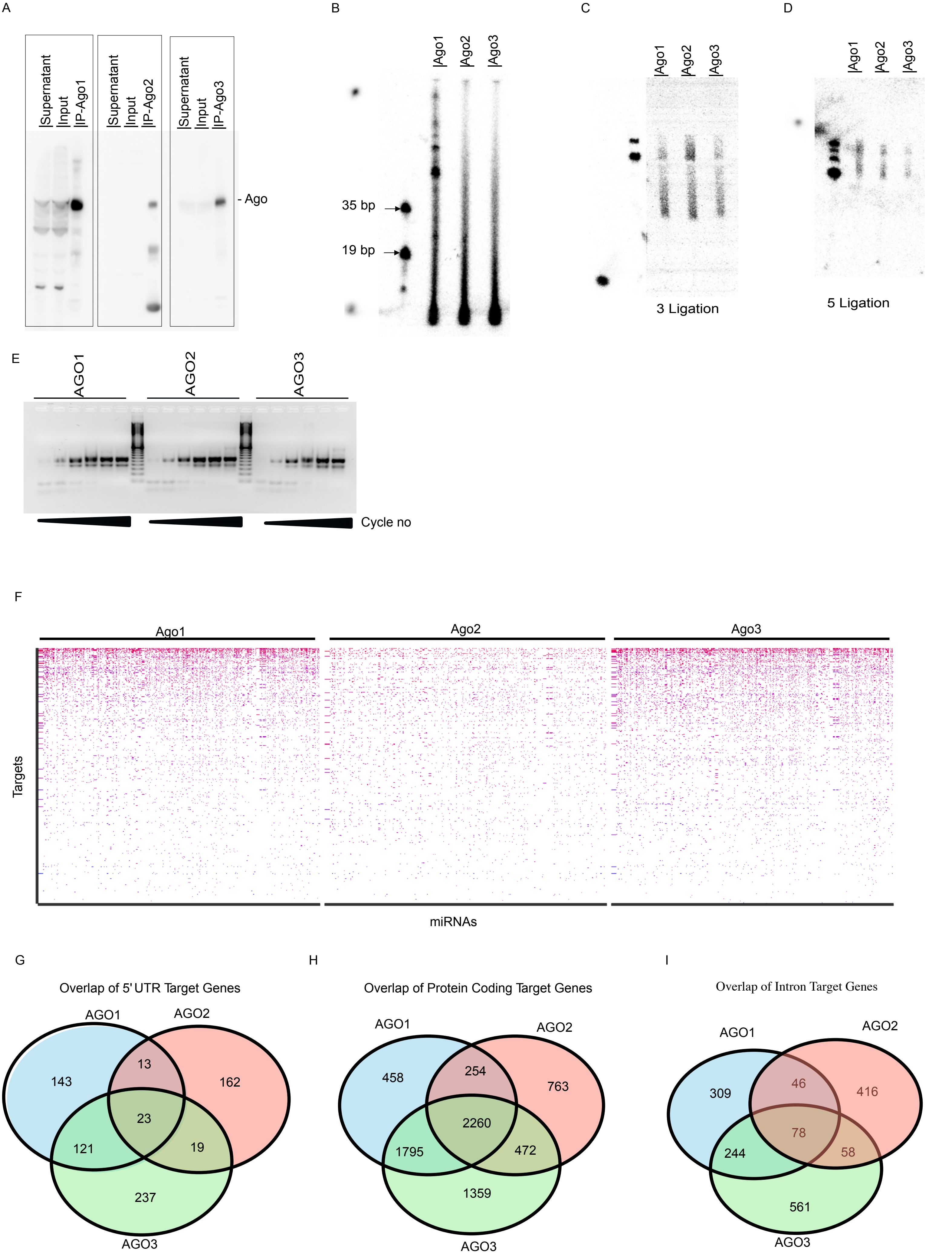
A
|Supernatant
|Input
|IP-Ago1
|Supernatant
|Input
|IP-Ago2
|Supernatant
|Input
|IP-Ago3
- Ago
B
|Ago1
|Ago2
|Ago3
35 bp
19 bp
C
|Ago1
|Ago2
|Ago3
3 Ligation
D
|Ago1
|Ago2
|Ago3
5 Ligation
E
AGO1
AGO2
AGO3
Cycle no
F
Ago1
Ago2
Ago3
Targets
miRNAs
G
Overlap of 5' UTR Target Genes
AGO1
AGO2
AGO3
143
13
162
23
121
19
237
H
Overlap of Protein Coding Target Genes
AGO1
AGO2
AGO3
458
254
763
2260
1795
472
1359
I
Overlap of Intron Target Genes
AGO1
AGO2
AGO3
309
46
416
78
244
58
561

Figure S2

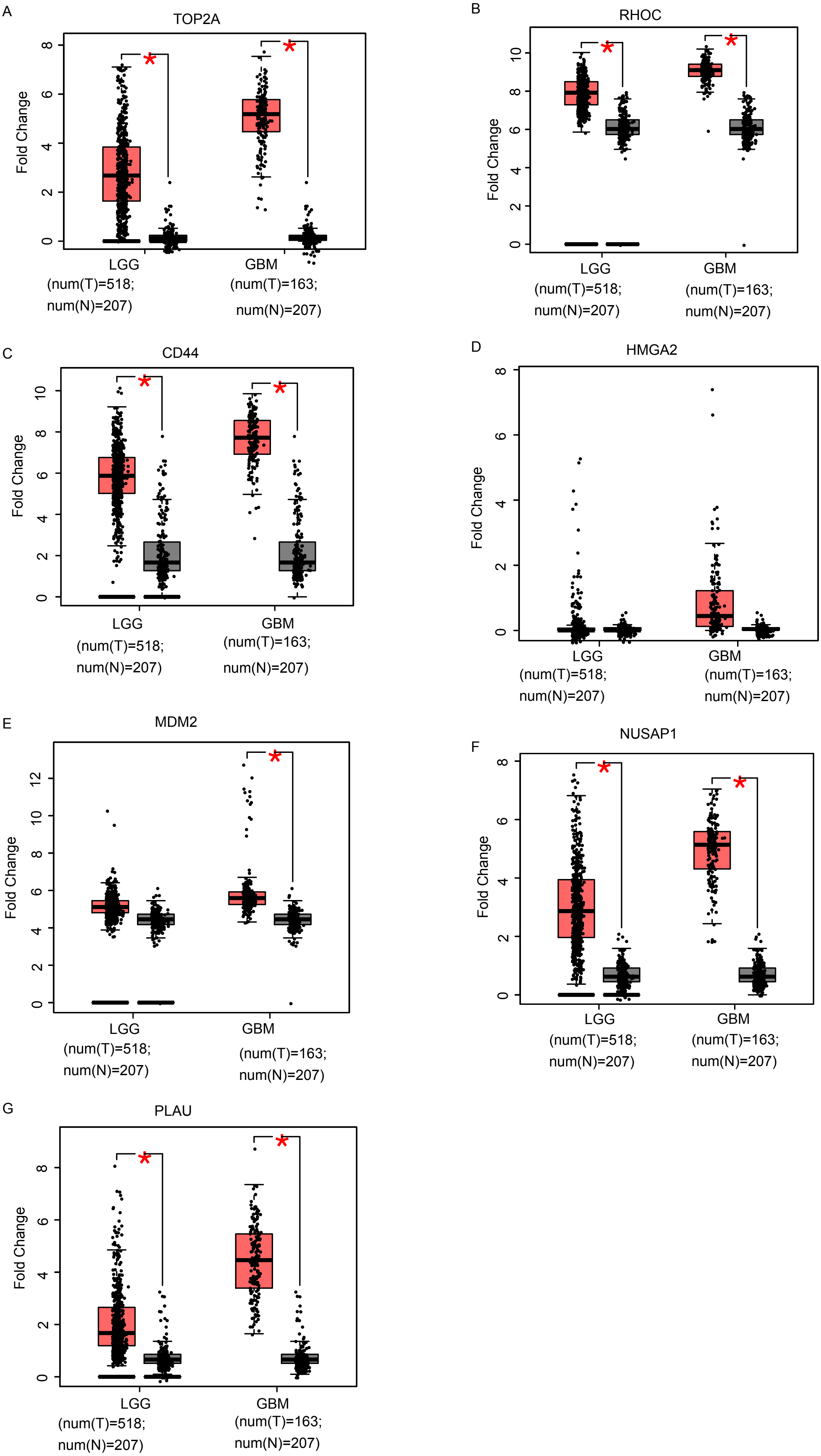
A
TOP2A
Fold Change
0
2
4
6
8
LGG
(num(T)=518;
num(N)=207)
GBM
(num(T)=163;
num(N)=207)
B
RHOC
Fold Change
0
2
4
6
8
10
LGG
(num(T)=518;
num(N)=207)
GBM
(num(T)=163;
num(N)=207)
C
CD44
Fold Change
0
2
4
6
8
10
LGG
(num(T)=518;
num(N)=207)
GBM
(num(T)=163;
num(N)=207)
D
HMGA2
Fold Change
0
2
4
6
8
LGG
(num(T)=518;
num(N)=207)
GBM
(num(T)=163;
num(N)=207)
E
MDM2
Fold Change
0
2
4
6
8
10
12
LGG
(num(T)=518;
num(N)=207)
GBM
(num(T)=163;
num(N)=207)
F
NUSAP1
Fold Change
0
2
4
6
8
LGG
(num(T)=518;
num(N)=207)
GBM
(num(T)=163;
num(N)=207)
G
PLAU
Fold Change
0
2
4
6
8
LGG
(num(T)=518;
num(N)=207)
GBM
(num(T)=163;
num(N)=207)

Figure S3

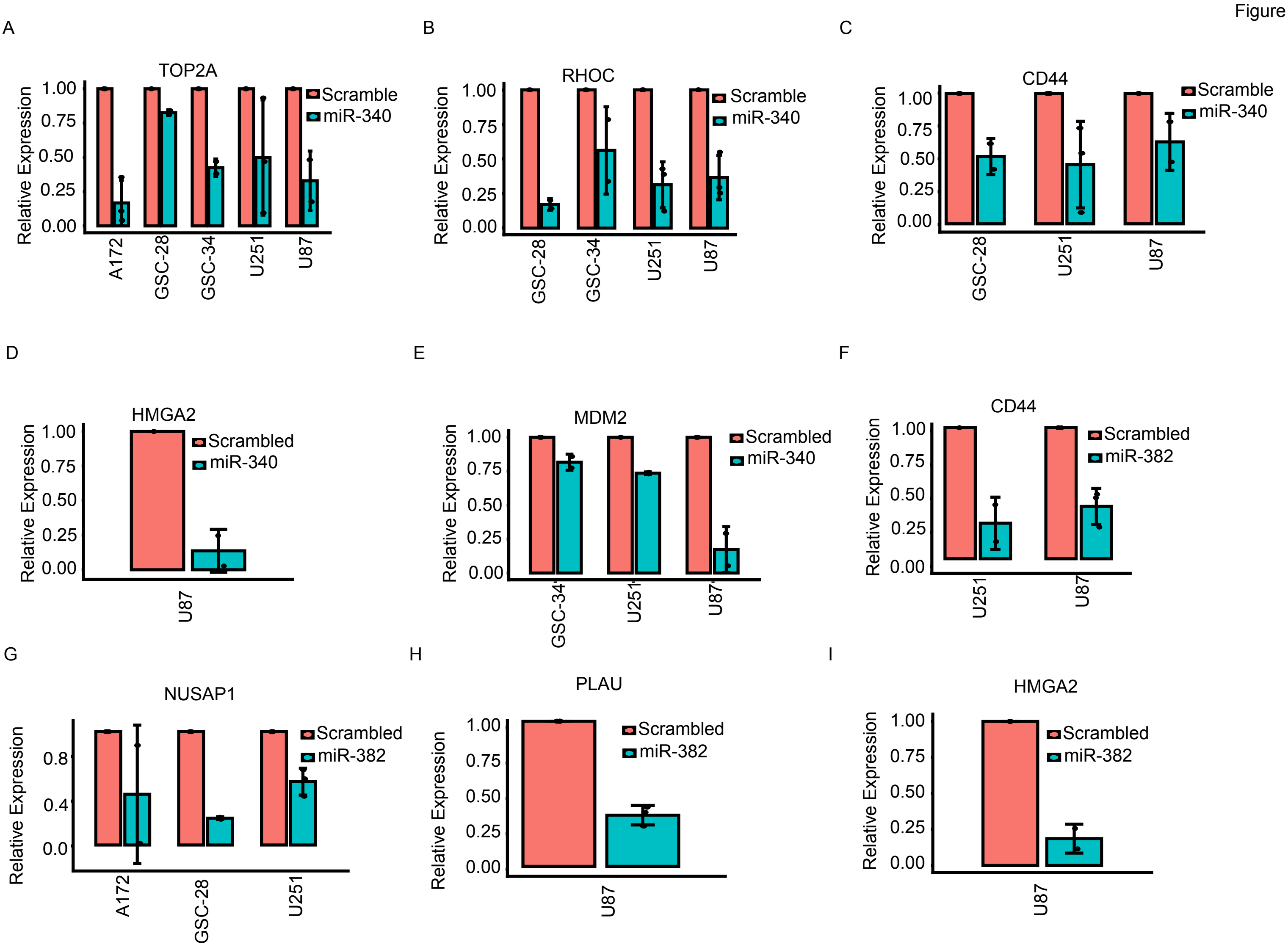
A
TOP2A
Relative Expression
1.00
0.75
0.50
0.25
0.00
Scramble
miR-340
A172
GSC-28
GSC-34
U251
U87
B
RHOC
Relative Expression
1.00
0.75
0.50
0.25
0.00
Scramble
miR-340
GSC-28
GSC-34
U251
U87
C
CD44
Relative Expression
1.00
0.75
0.50
0.25
0.00
Scramble
miR-340
GSC-28
U251
U87
D
HMGA2
Relative Expression
1.00
0.75
0.50
0.25
0.00
Scrambled
miR-340
U87
E
MDM2
Relative Expression
1.00
0.75
0.50
0.25
0.00
Scrambled
miR-340
GSC-34
U251
U87
F
CD44
Relative Expression
1.00
0.75
0.50
0.25
0.00
Scrambled
miR-382
U251
U87
G
NUSAP1
Relative Expression
0.8
0.4
0.0
Scrambled
miR-382
A172
GSC-28
U251
H
PLAU
Relative Expression
1.00
0.75
0.50
0.25
0.00
Scrambled
miR-382
U87
I
HMGA2
Relative Expression
1.00
0.75
0.50
0.25
0.00
Scrambled
miR-382
U87

Figure S4

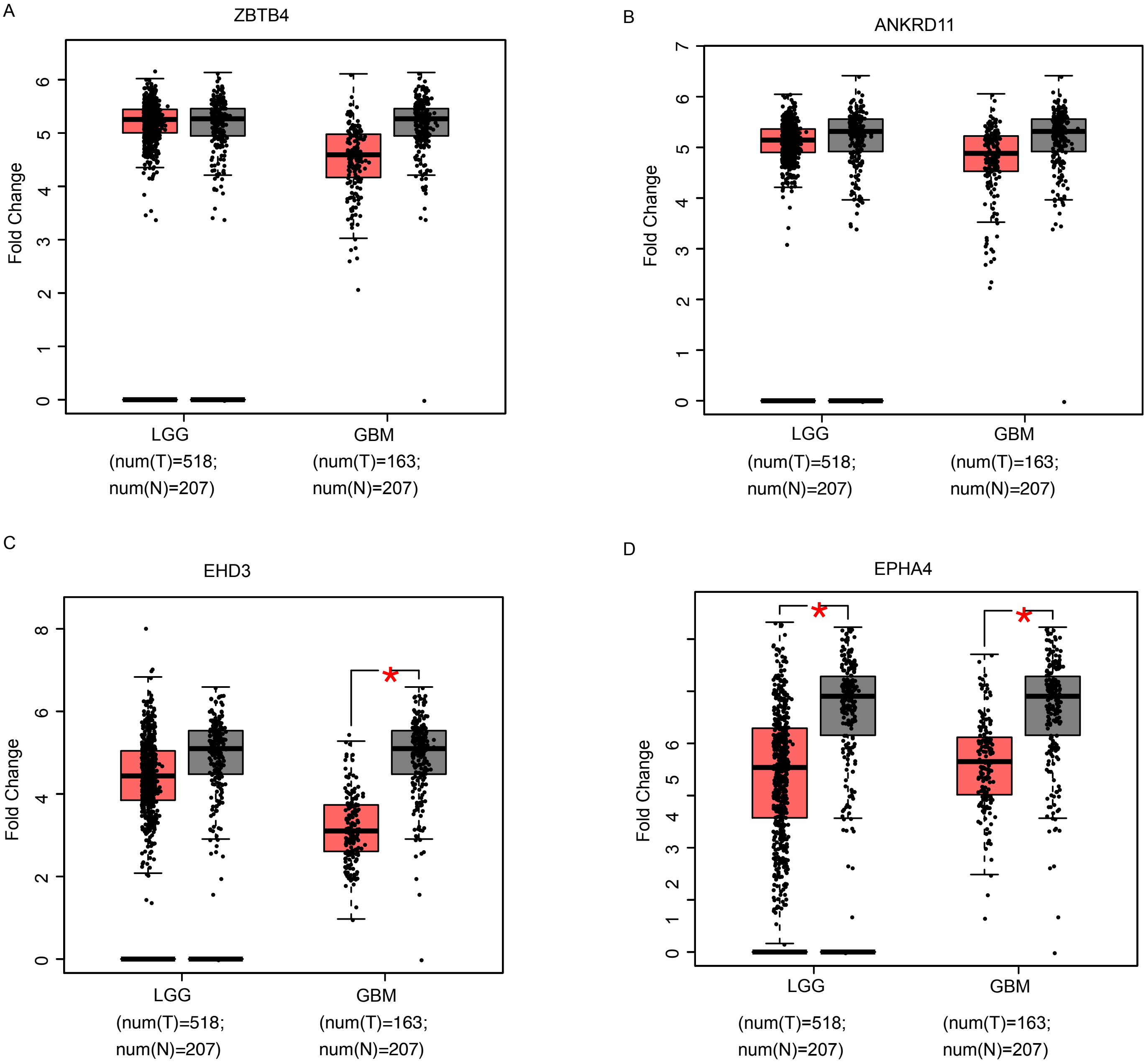
A
ZBTB4
Fold Change
0
1
2
3
4
5
6
LGG
(num(T)=518;
num(N)=207)
GBM
(num(T)=163;
num(N)=207)
B
ANKRD11
Fold Change
0
1
2
3
4
5
6
7
LGG
(num(T)=518;
num(N)=207)
GBM
(num(T)=163;
num(N)=207)
C
EHD3
Fold Change
0
2
4
6
8
*
LGG
(num(T)=518;
num(N)=207)
GBM
(num(T)=163;
num(N)=207)
D
EPHA4
Fold Change
0
1
2
3
4
5
6
*
*
LGG
(num(T)=518;
num(N)=207)
GBM
(num(T)=163;
num(N)=207)

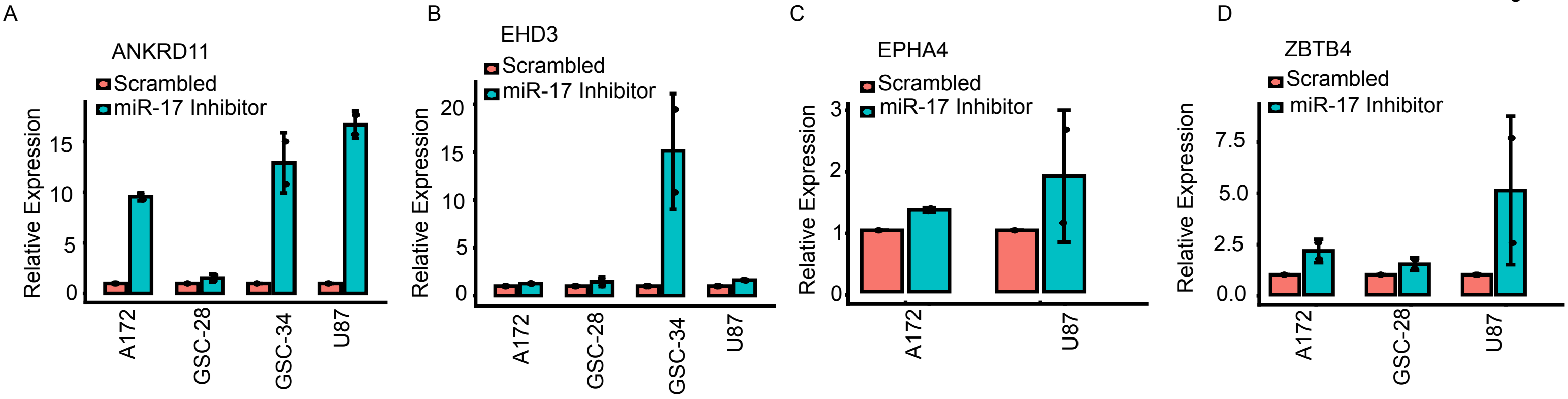
A
ANKRD11
Scrambled
miR-17 Inhibitor
Relative Expression
0
5
10
15
A172
GSC-28
GSC-34
U87
B
EHD3
Scrambled
miR-17 Inhibitor
Relative Expression
0
5
10
15
20
A172
GSC-28
GSC-34
U87
C
EPHA4
Scrambled
miR-17 Inhibitor
Relative Expression
0
1
2
3
A172
U87
D
ZBTB4
Scrambled
miR-17 Inhibitor
Relative Expression
0.0
2.5
5.0
7.5
A172
GSC-28
U87

Figure S6

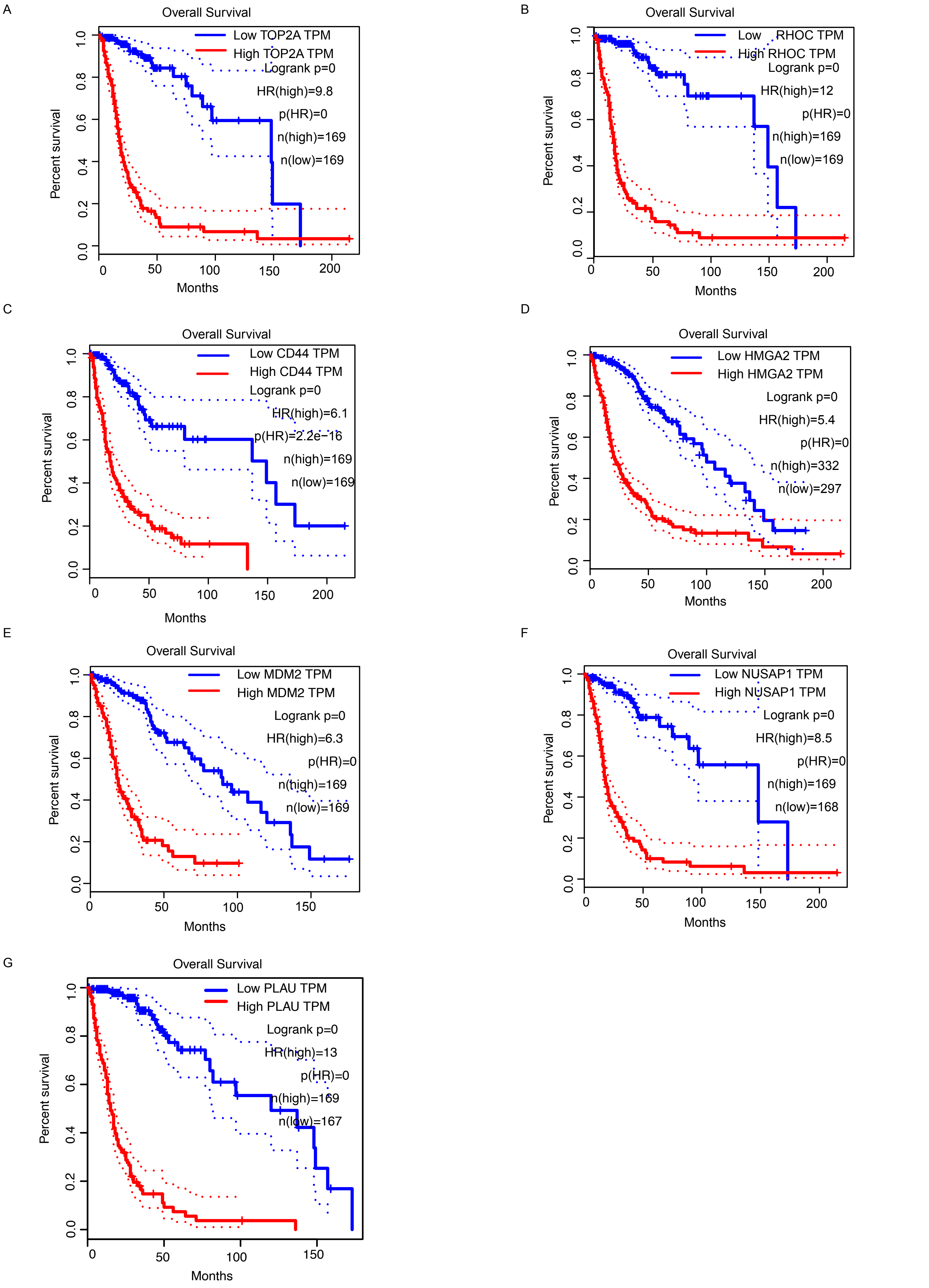
A
Overall Survival
Low TOP2A TPM
High TOP2A TPM
Logrank p=0
HR(high)=9.8
p(HR)=0
n(high)=169
n(low)=169
Percent survival
Months
B
Overall Survival
Low RHOC TPM
High RHOC TPM
Logrank p=0
HR(high)=12
p(HR)=0
n(high)=169
n(low)=169
Percent survival
Months
C
Overall Survival
Low CD44 TPM
High CD44 TPM
Logrank p=0
HR(high)=6.1
p(HR)=2.2e−16
n(high)=169
n(low)=169
Percent survival
Months
D
Overall Survival
Low HMGA2 TPM
High HMGA2 TPM
Logrank p=0
HR(high)=5.4
p(HR)=0
n(high)=332
n(low)=297
Percent survival
Months
E
Overall Survival
Low MDM2 TPM
High MDM2 TPM
Logrank p=0
HR(high)=6.3
p(HR)=0
n(high)=169
n(low)=169
Percent survival
Months
F
Overall Survival
Low NUSAP1 TPM
High NUSAP1 TPM
Logrank p=0
HR(high)=8.5
p(HR)=0
n(high)=169
n(low)=168
Percent survival
Months
G
Overall Survival
Low PLAU TPM
High PLAU TPM
Logrank p=0
HR(high)=13
p(HR)=0
n(high)=169
n(low)=167
Percent survival
Months

Figure S7

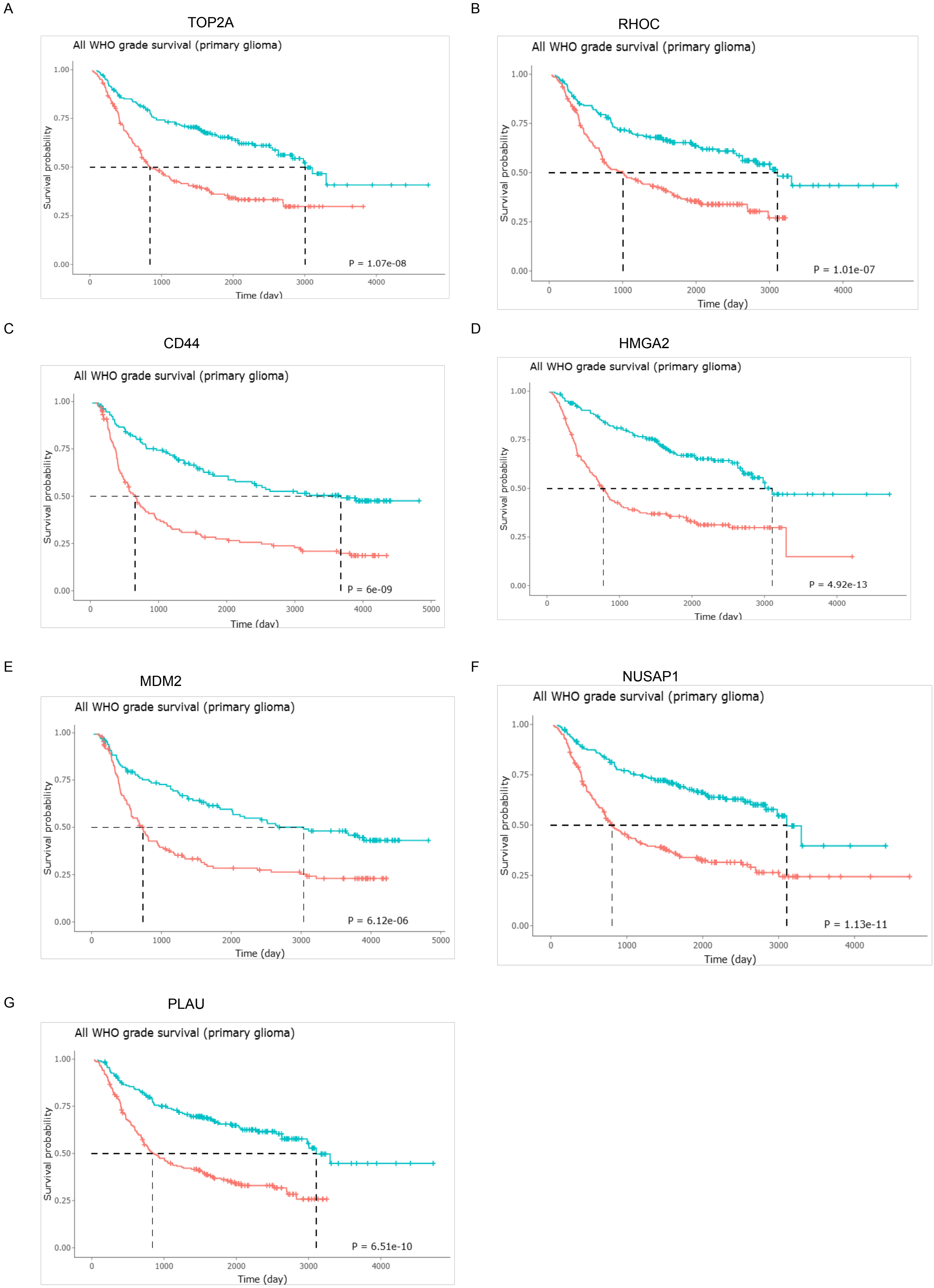
A
TOP2A
All WHO grade survival (primary glioma)
Survival probability
Time (day)
P = 1.07e-08
B
RHOC
All WHO grade survival (primary glioma)
Survival probability
Time (day)
P = 1.01e-07
C
CD44
All WHO grade survival (primary glioma)
Survival probability
Time (day)
P = 6e-09
D
HMGA2
All WHO grade survival (primary glioma)
Survival probability
Time (day)
P = 4.92e-13
E
MDM2
All WHO grade survival (primary glioma)
Survival probability
Time (day)
P = 6.12e-06
F
NUSAP1
All WHO grade survival (primary glioma)
Survival probability
Time (day)
P = 1.13e-11
G
PLAU
All WHO grade survival (primary glioma)
Survival probability
Time (day)
P = 6.51e-10

Figure S8

A

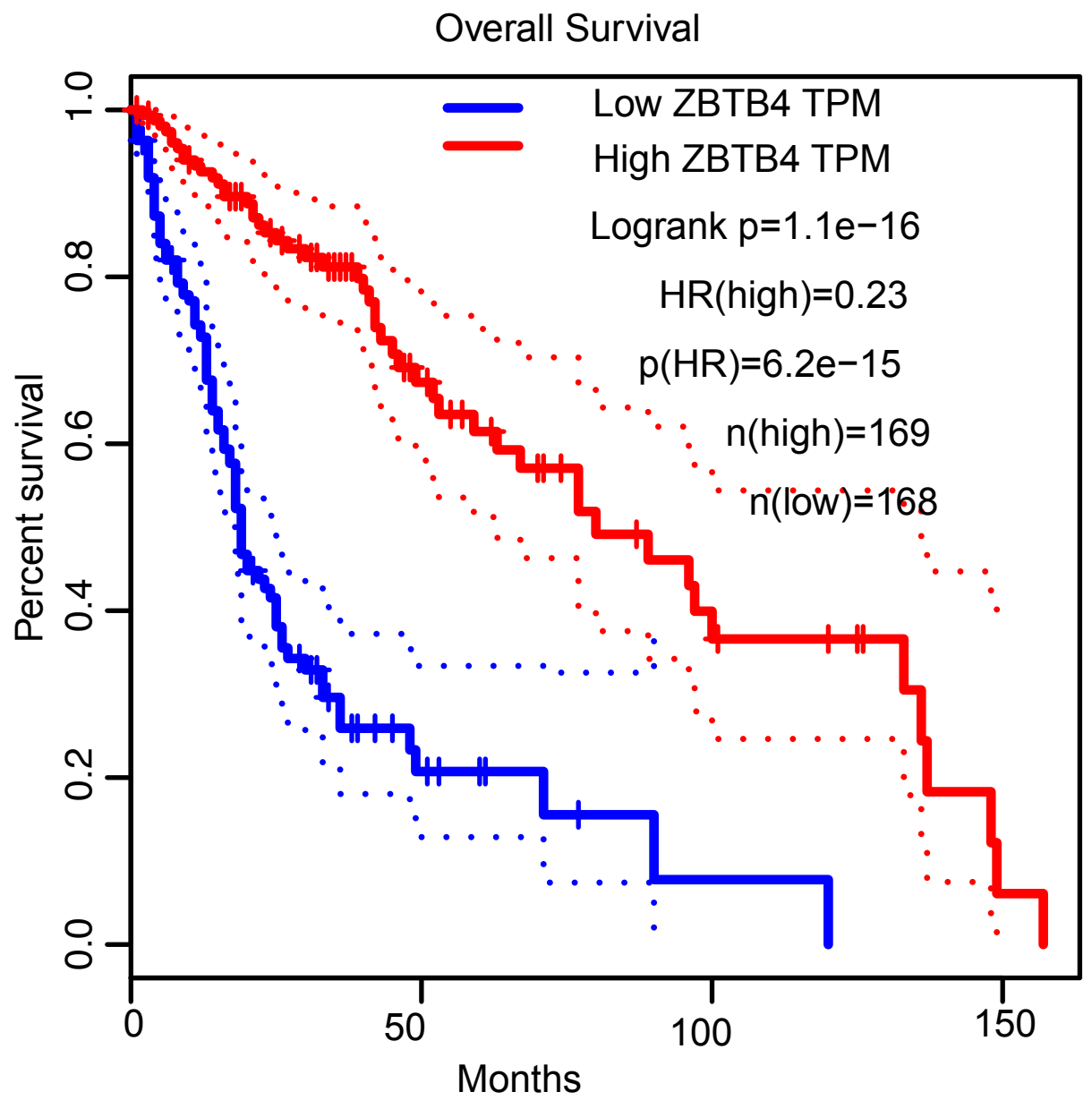
Overall Survival
Low ZBTB4 TPM
High ZBTB4 TPM
Logrank p=1.1e−16
HR(high)=0.23
p(HR)=6.2e−15
n(high)=169
n(low)=168
Percent survival
0.0
0.2
0.4
0.6
0.8
1.0
0
50
100
150
Months

B

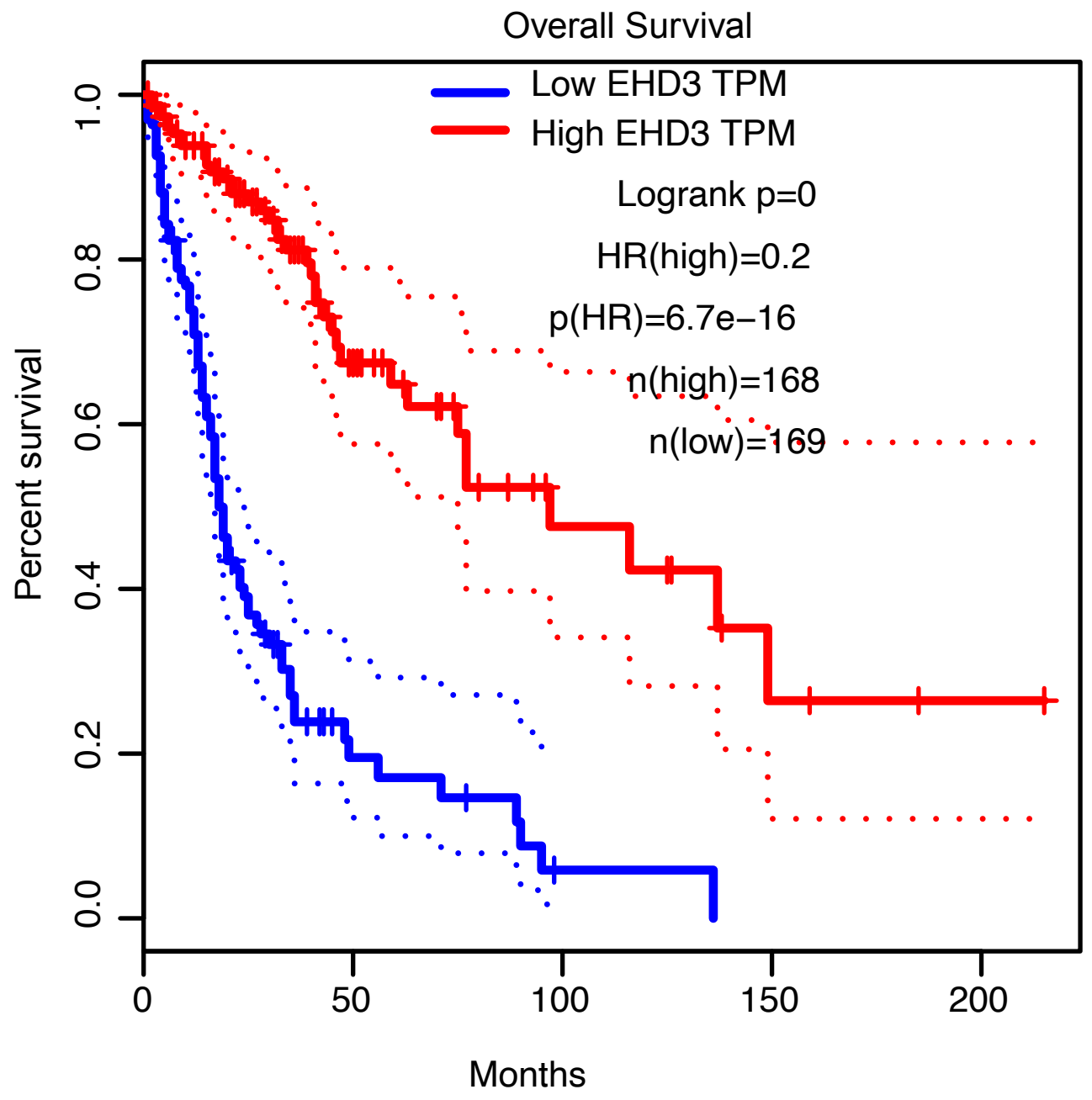
Overall Survival
Low EHD3 TPM
High EHD3 TPM
Logrank p=0
HR(high)=0.2
p(HR)=6.7e−16
n(high)=168
n(low)=169
Percent survival
0.0
0.2
0.4
0.6
0.8
1.0
0
50
100
150
200
Months

Figure S9

A

ZBTB4

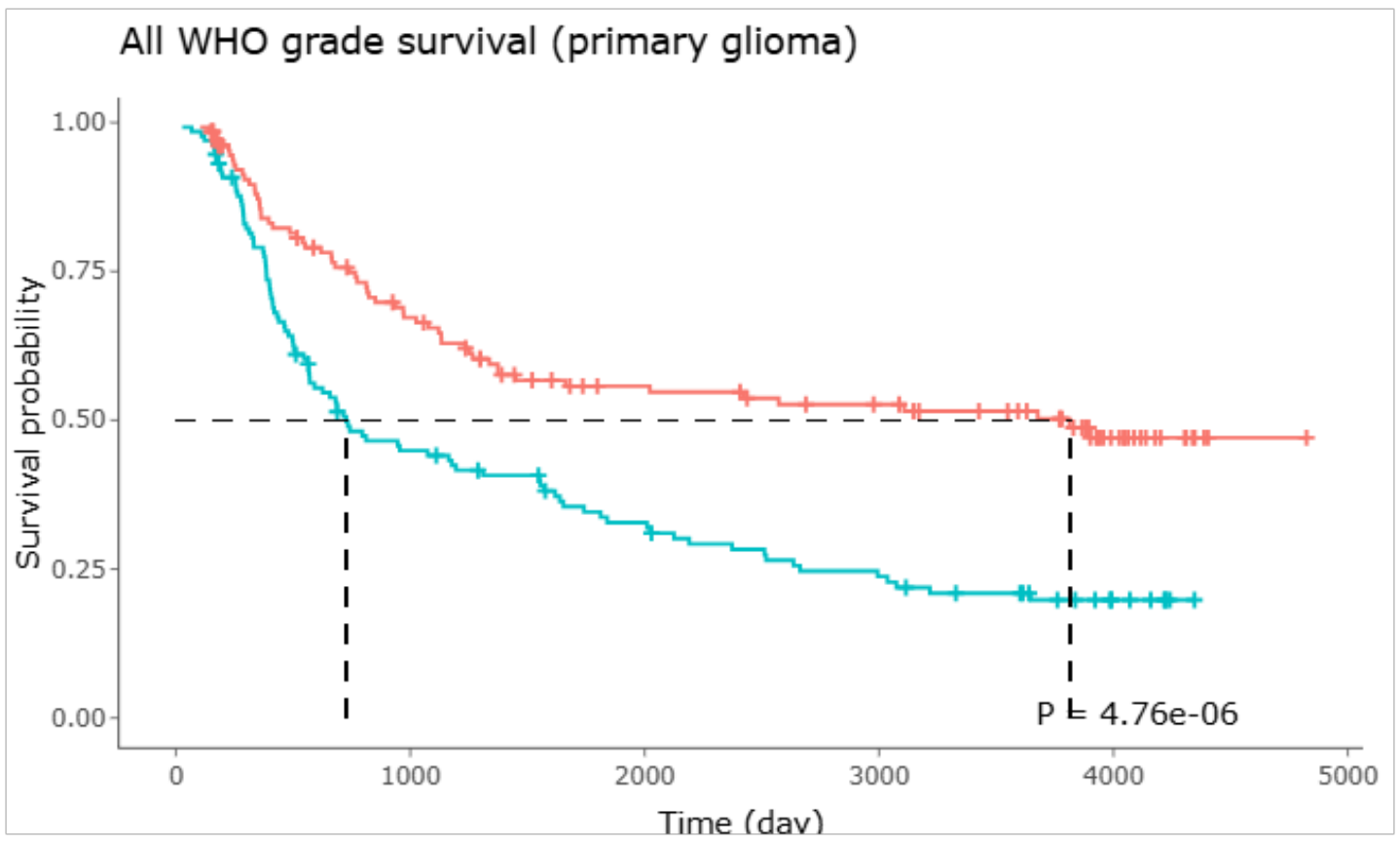

B

EHD3

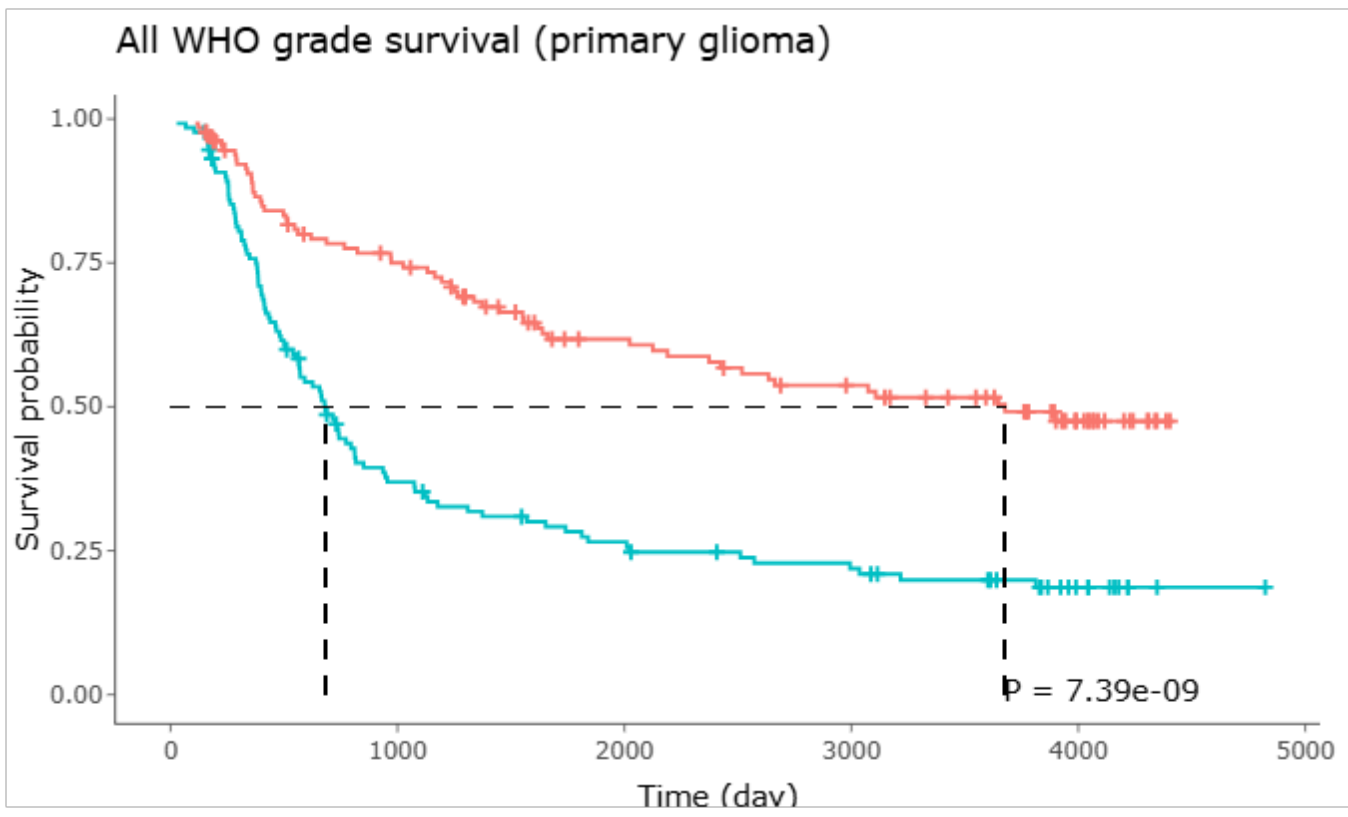

Figure S10

A

TOP2A

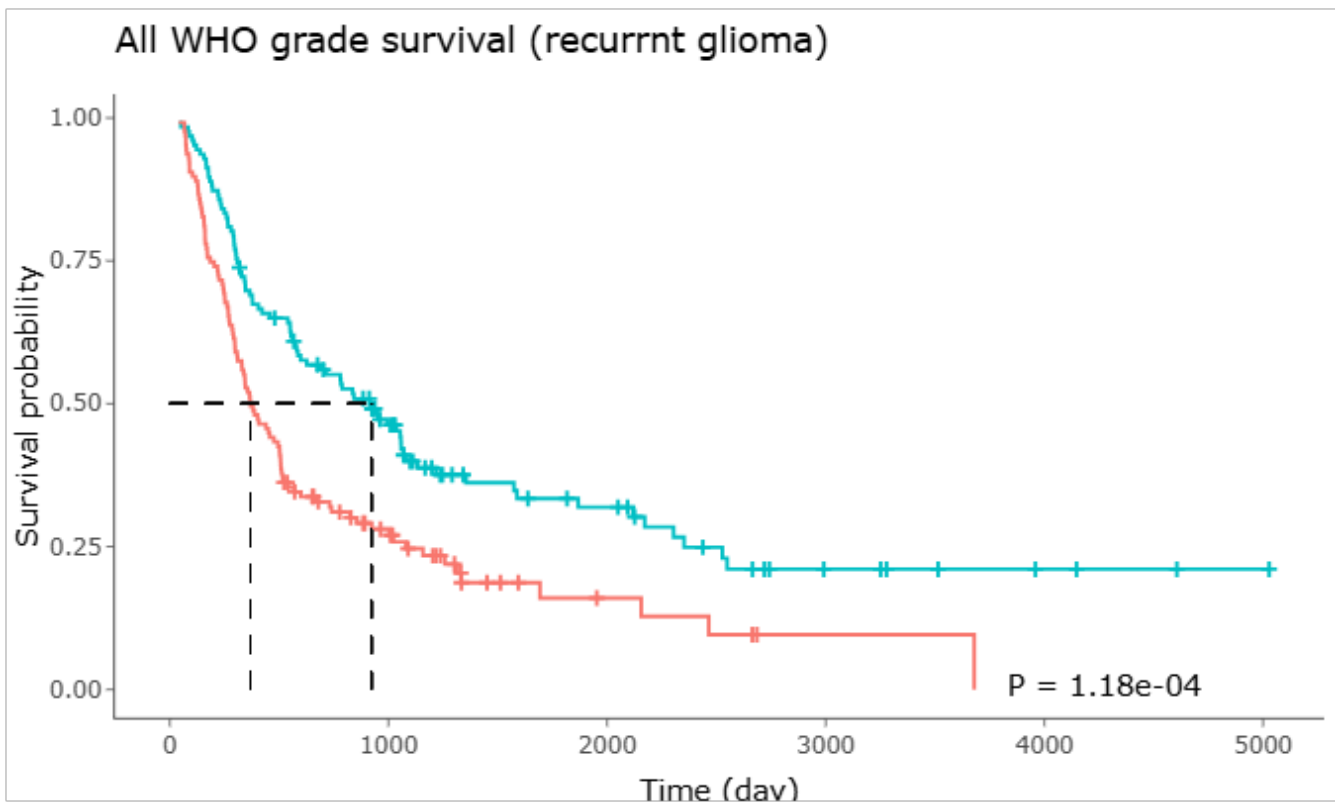

B

RHOC

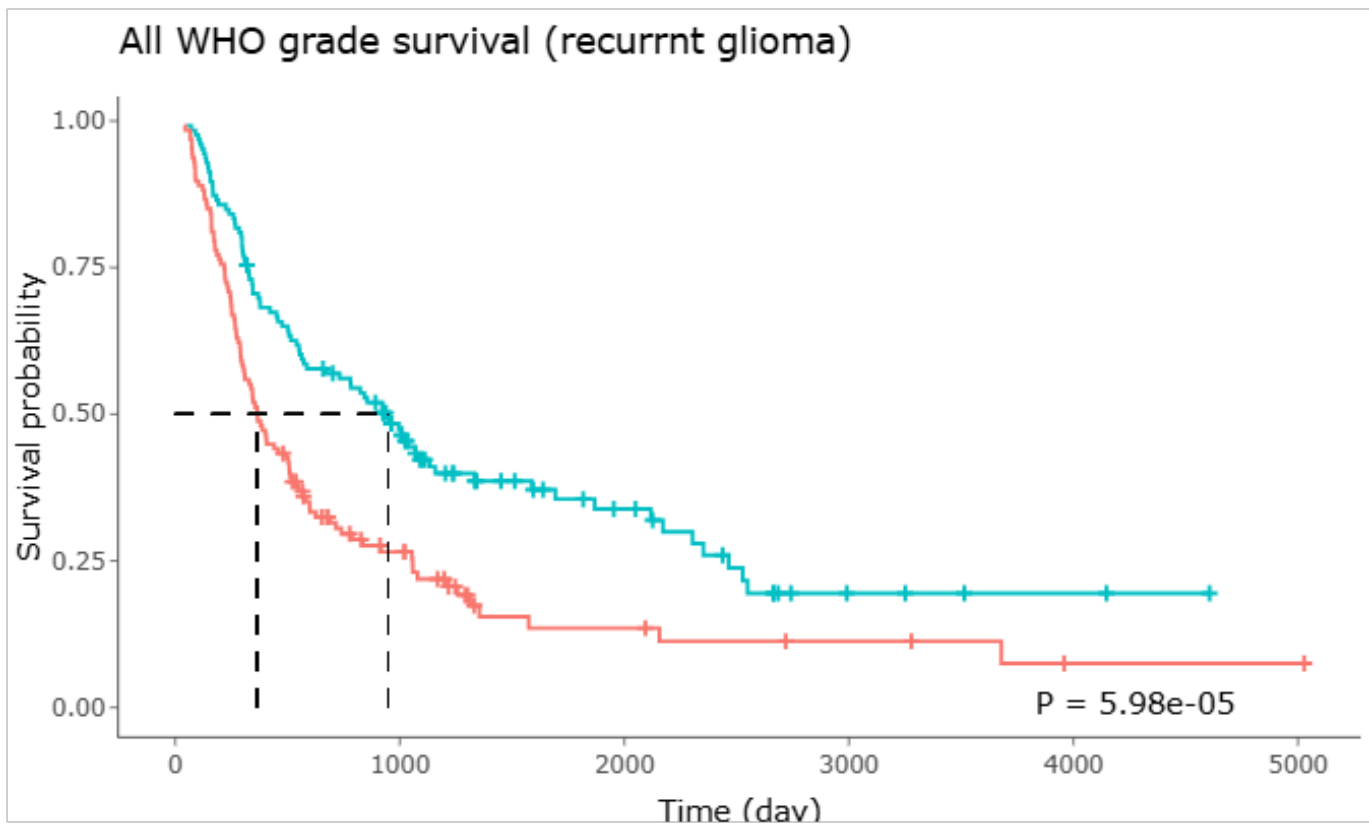

C

CD44

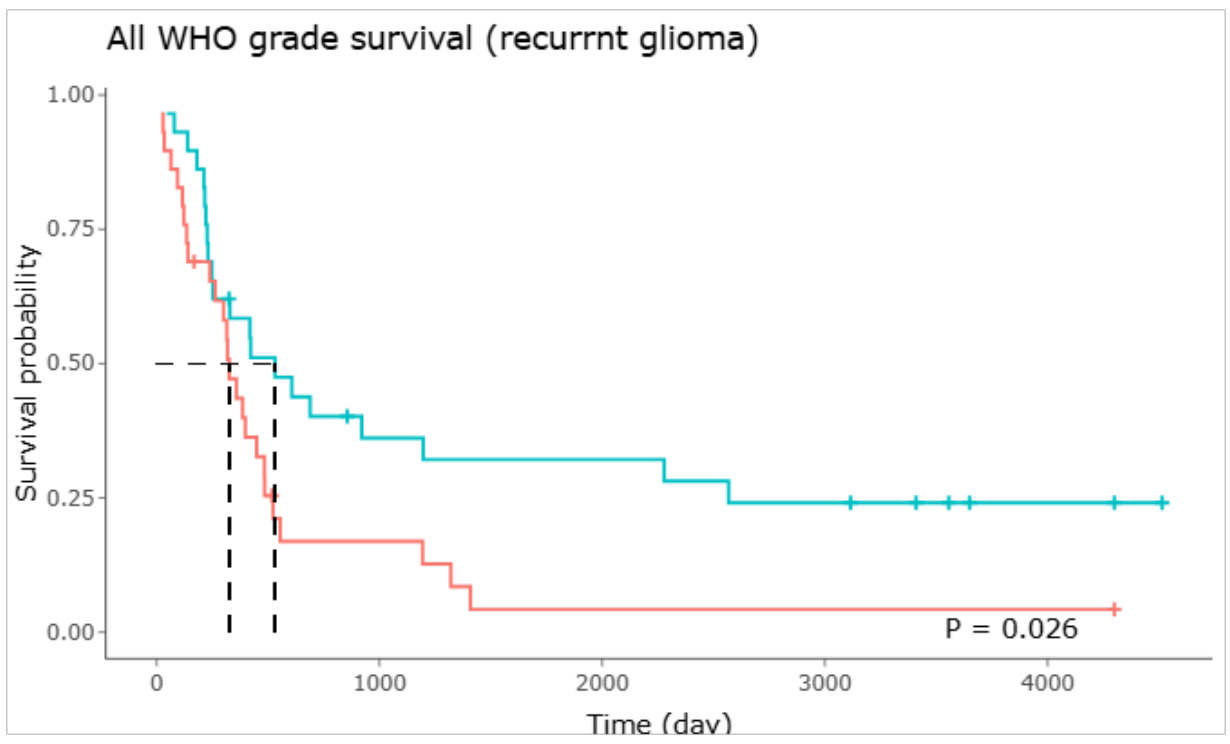

D

NUSAP1

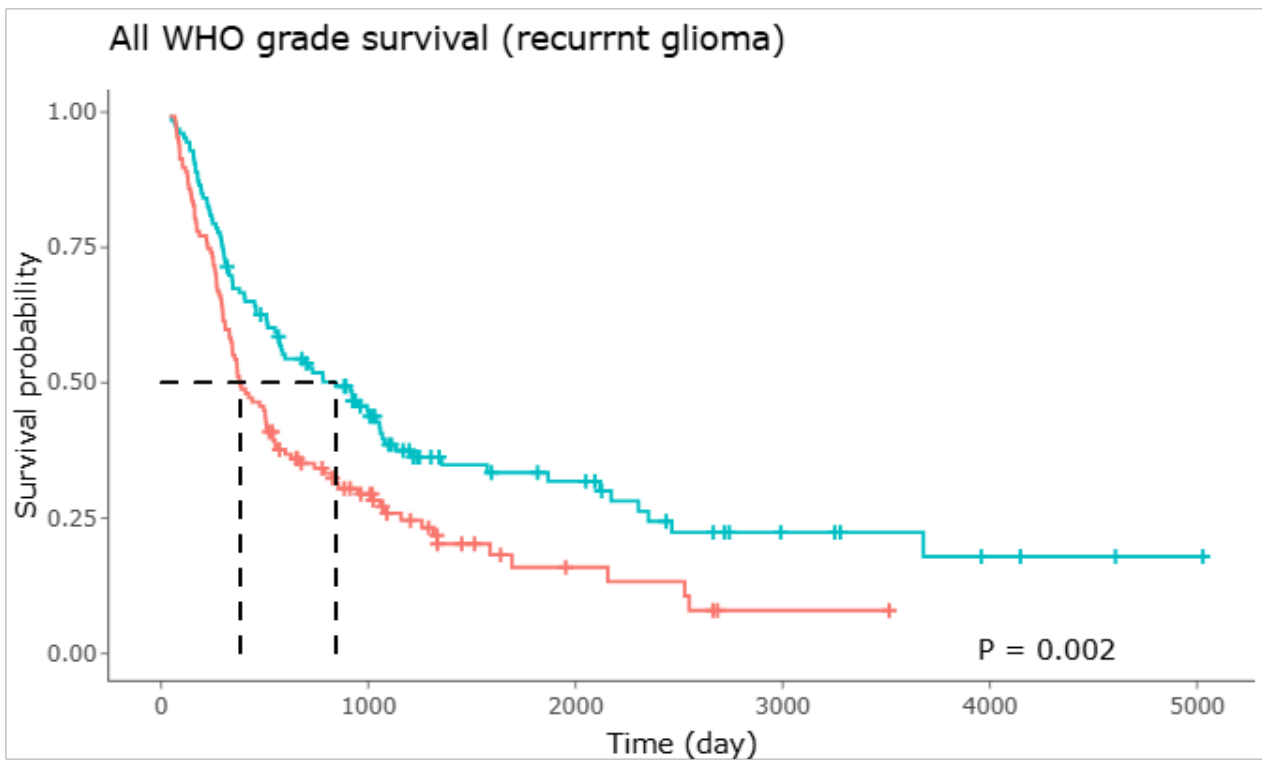

E

HMGA2

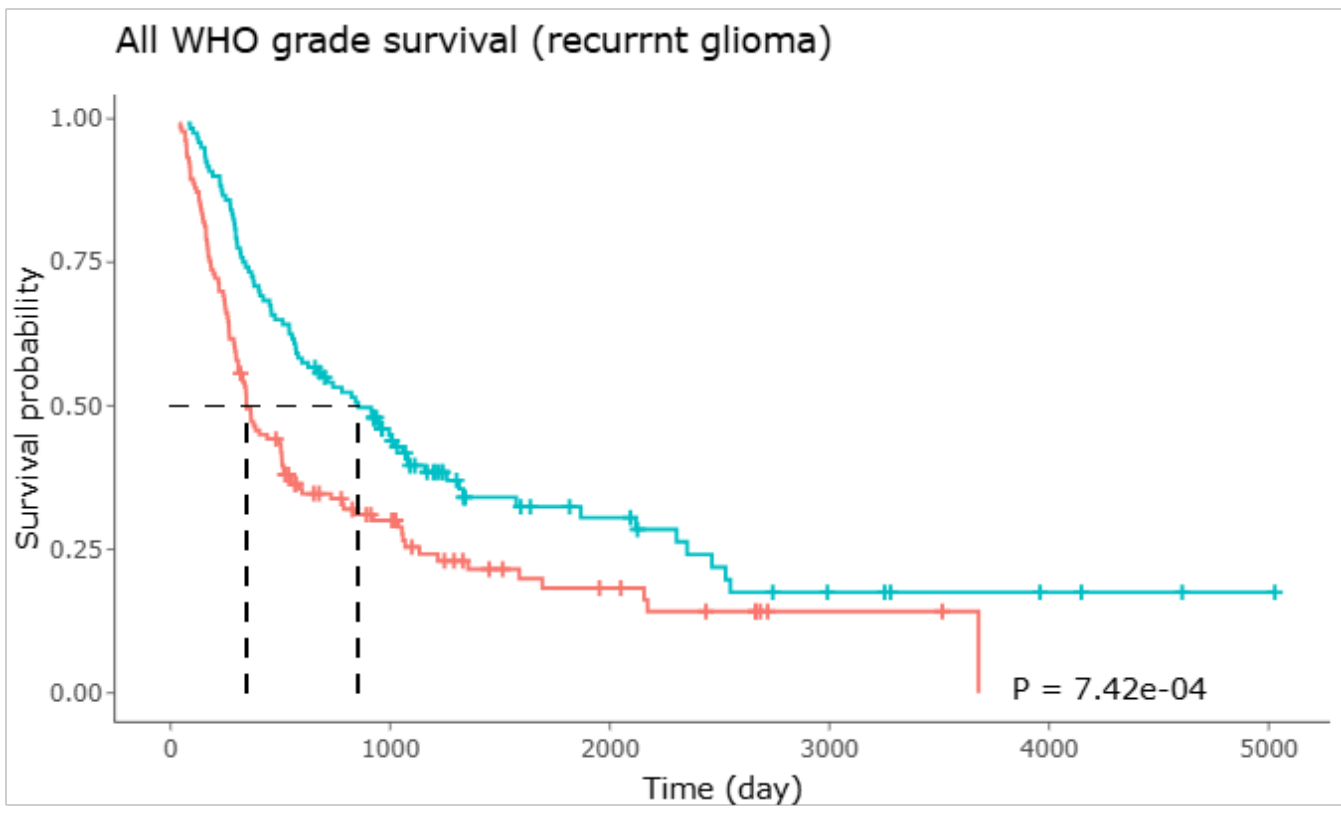

F

PLAU

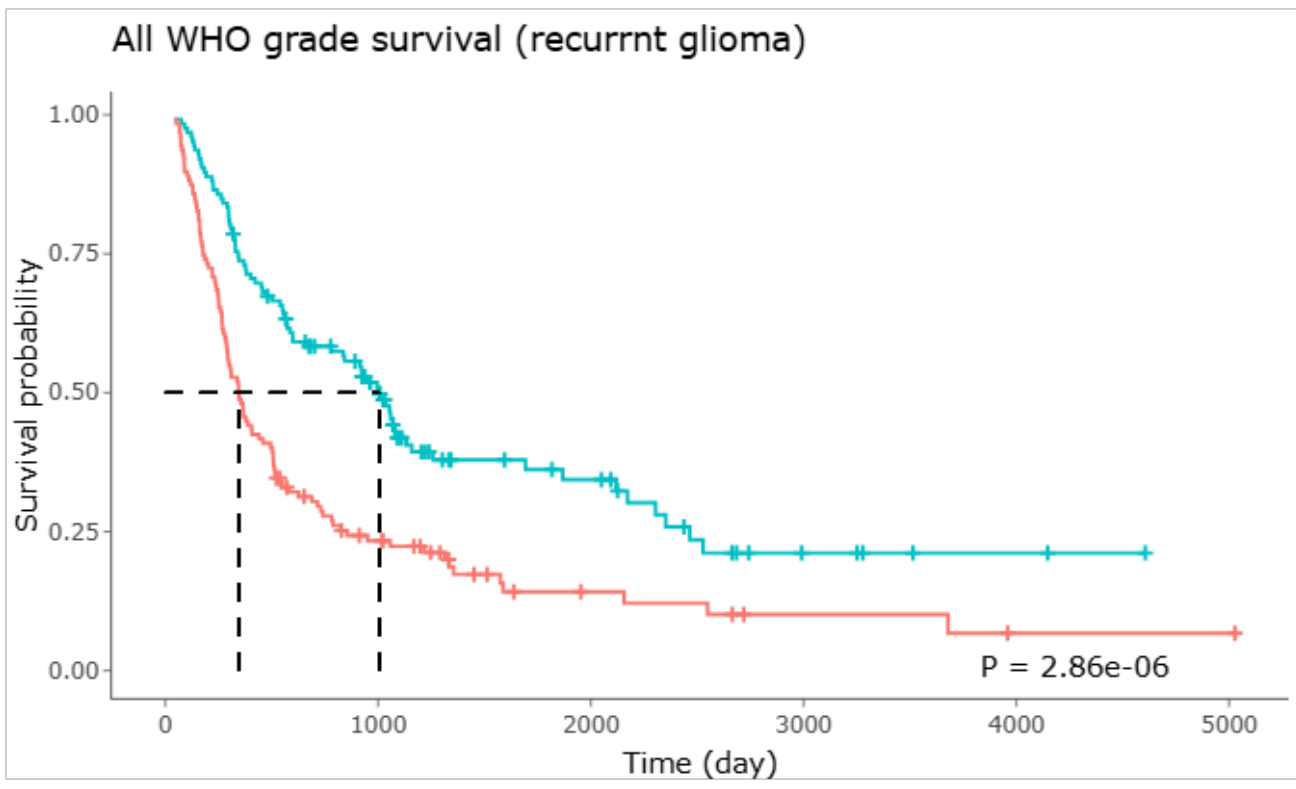

Figure S11

EHD3

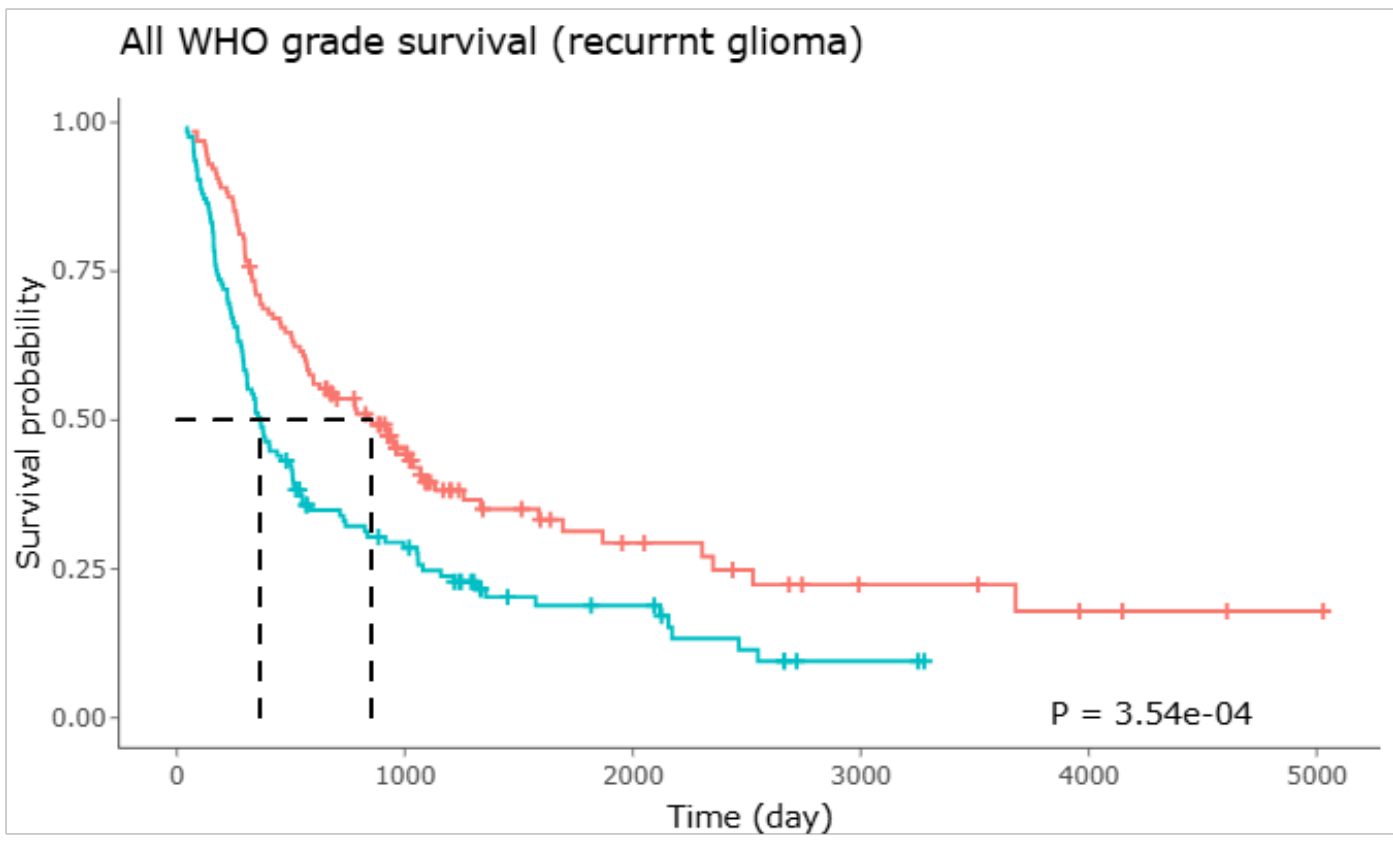

Figure S12

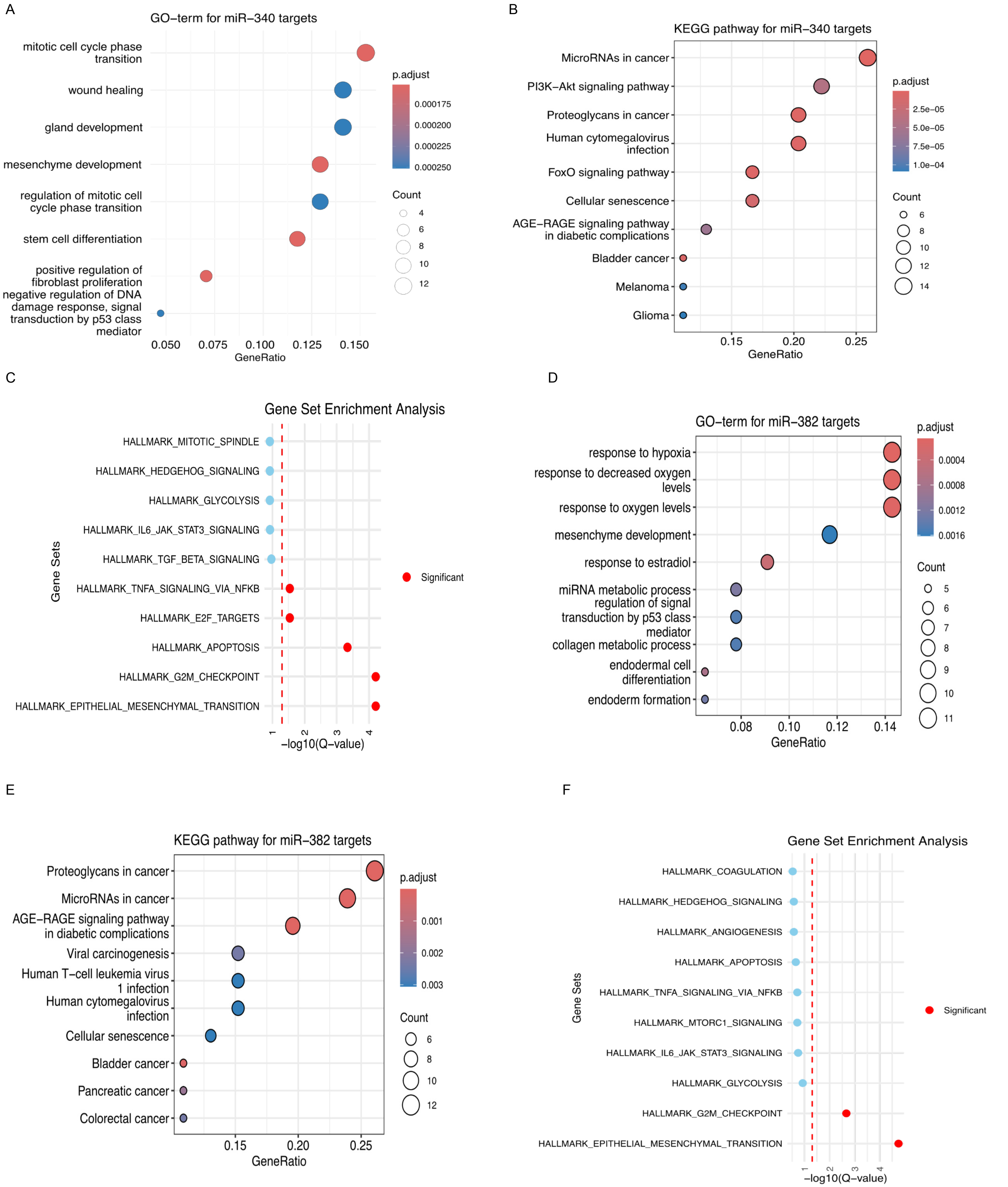
A
GO−term for miR−340 targets
mitotic cell cycle phase transition
wound healing
gland development
mesenchyme development
regulation of mitotic cell cycle phase transition
stem cell differentiation
positive regulation of fibroblast proliferation
negative regulation of DNA damage response, signal transduction by p53 class mediator
0.050
0.075
0.100
0.125
0.150
GeneRatio
p.adjust
0.000175
0.000200
0.000225
0.000250
Count
4
6
8
10
12
B
KEGG pathway for miR−340 targets
MicroRNAs in cancer
PI3K−Akt signaling pathway
Proteoglycans in cancer
Human cytomegalovirus infection
FoxO signaling pathway
Cellular senescence
AGE−RAGE signaling pathway in diabetic complications
Bladder cancer
Melanoma
Glioma
0.15
0.20
0.25
GeneRatio
p.adjust
2.5e−05
5.0e−05
7.5e−05
1.0e−04
Count
6
8
10
12
14
C
Gene Set Enrichment Analysis
Gene Sets
HALLMARK_MITOTIC_SPINDLE
HALLMARK_HEDGEHOG_SIGNALING
HALLMARK_GLYCOLYSIS
HALLMARK_IL6_JAK_STAT3_SIGNALING
HALLMARK_TGF_BETA_SIGNALING
HALLMARK_TNFA_SIGNALING_VIA_NFKB
HALLMARK_E2F_TARGETS
HALLMARK_APOPTOSIS
HALLMARK_G2M_CHECKPOINT
HALLMARK_EPITHELIAL_MESENCHYMAL_TRANSITION
1
2
3
4
−log10(Q−value)
Significant
D
GO−term for miR−382 targets
response to hypoxia
response to decreased oxygen levels
response to oxygen levels
mesenchyme development
response to estradiol
miRNA metabolic process
regulation of signal transduction by p53 class mediator
collagen metabolic process
endodermal cell differentiation
endoderm formation
0.08
0.10
0.12
0.14
GeneRatio
p.adjust
0.0004
0.0008
0.0012
0.0016
Count
5
6
7
8
9
10
11
E
KEGG pathway for miR−382 targets
Proteoglycans in cancer
MicroRNAs in cancer
AGE−RAGE signaling pathway in diabetic complications
Viral carcinogenesis
Human T−cell leukemia virus 1 infection
Human cytomegalovirus infection
Cellular senescence
Bladder cancer
Pancreatic cancer
Colorectal cancer
0.15
0.20
0.25
GeneRatio
p.adjust
0.001
0.002
0.003
Count
6
8
10
12
F
Gene Set Enrichment Analysis
Gene Sets
HALLMARK_COAGULATION
HALLMARK_HEDGEHOG_SIGNALING
HALLMARK_ANGIOGENESIS
HALLMARK_APOPTOSIS
HALLMARK_TNFA_SIGNALING_VIA_NFKB
HALLMARK_MTORC1_SIGNALING
HALLMARK_IL6_JAK_STAT3_SIGNALING
HALLMARK_GLYCOLYSIS
HALLMARK_G2M_CHECKPOINT
HALLMARK_EPITHELIAL_MESENCHYMAL_TRANSITION
1
2
3
4
−log10(Q−value)
Significant

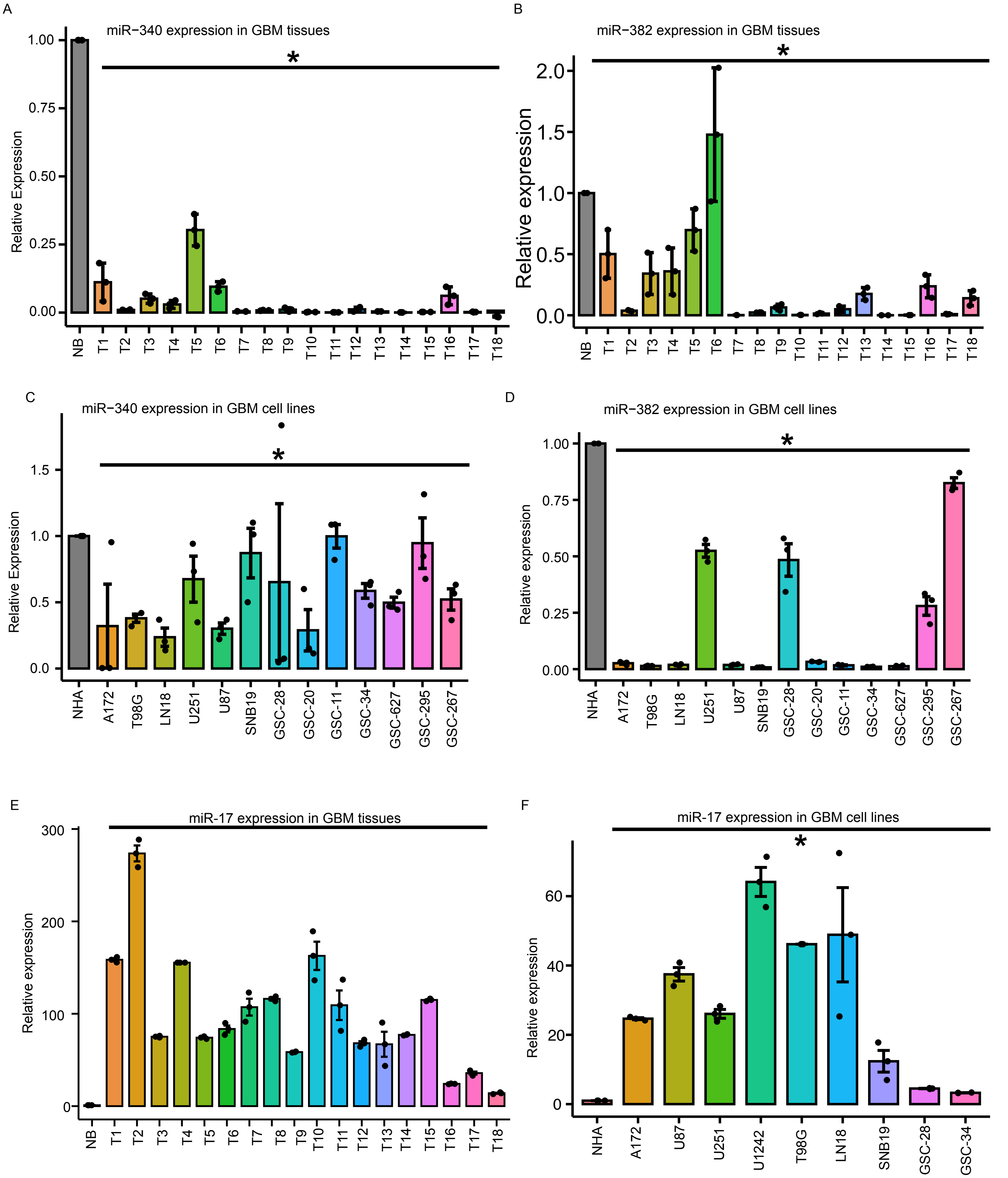
A
miR−340 expression in GBM tissues
Relative Expression
*
NB
T1
T2
T3
T4
T5
T6
T7
T8
T9
T10
T11
T12
T13
T14
T15
T16
T17
T18
B
miR−382 expression in GBM tissues
Relative expression
*
C
miR−340 expression in GBM cell lines
Relative Expression
*
NHA
A172
T98G
LN18
U251
U87
SNB19
GSC-28
GSC-20
GSC-11
GSC-34
GSC-627
GSC-295
GSC-267
D
miR−382 expression in GBM cell lines
Relative expression
*
E
miR-17 expression in GBM tissues
Relative expression
F
miR-17 expression in GBM cell lines
Relative expression
*
NHA
A172
U87
U251
U1242
T98G
LN18
SNB19
GSC-28
GSC-34

Figure S14

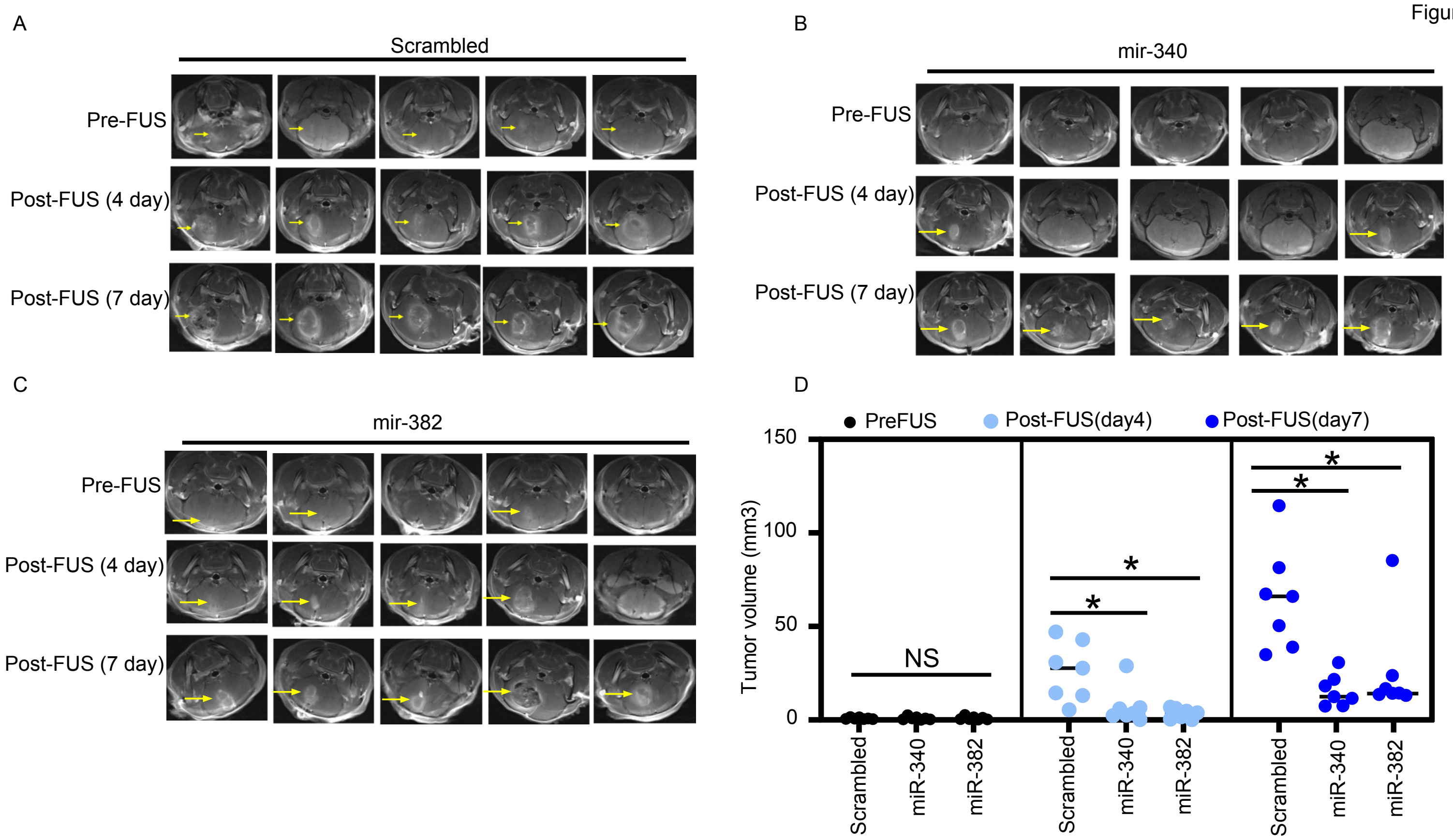

A
Scrambled
Pre-FUS
Post-FUS (4 day)
Post-FUS (7 day)
B
mir-340
Pre-FUS
Post-FUS (4 day)
Post-FUS (7 day)
C
mir-382
Pre-FUS
Post-FUS (4 day)
Post-FUS (7 day)
D
PreFUS
Post-FUS(day4)
Post-FUS(day7)
Tumor volume (mm3)
150
100
50
0
NS
*
Scrambled
miR-340
miR-382

Figure S15

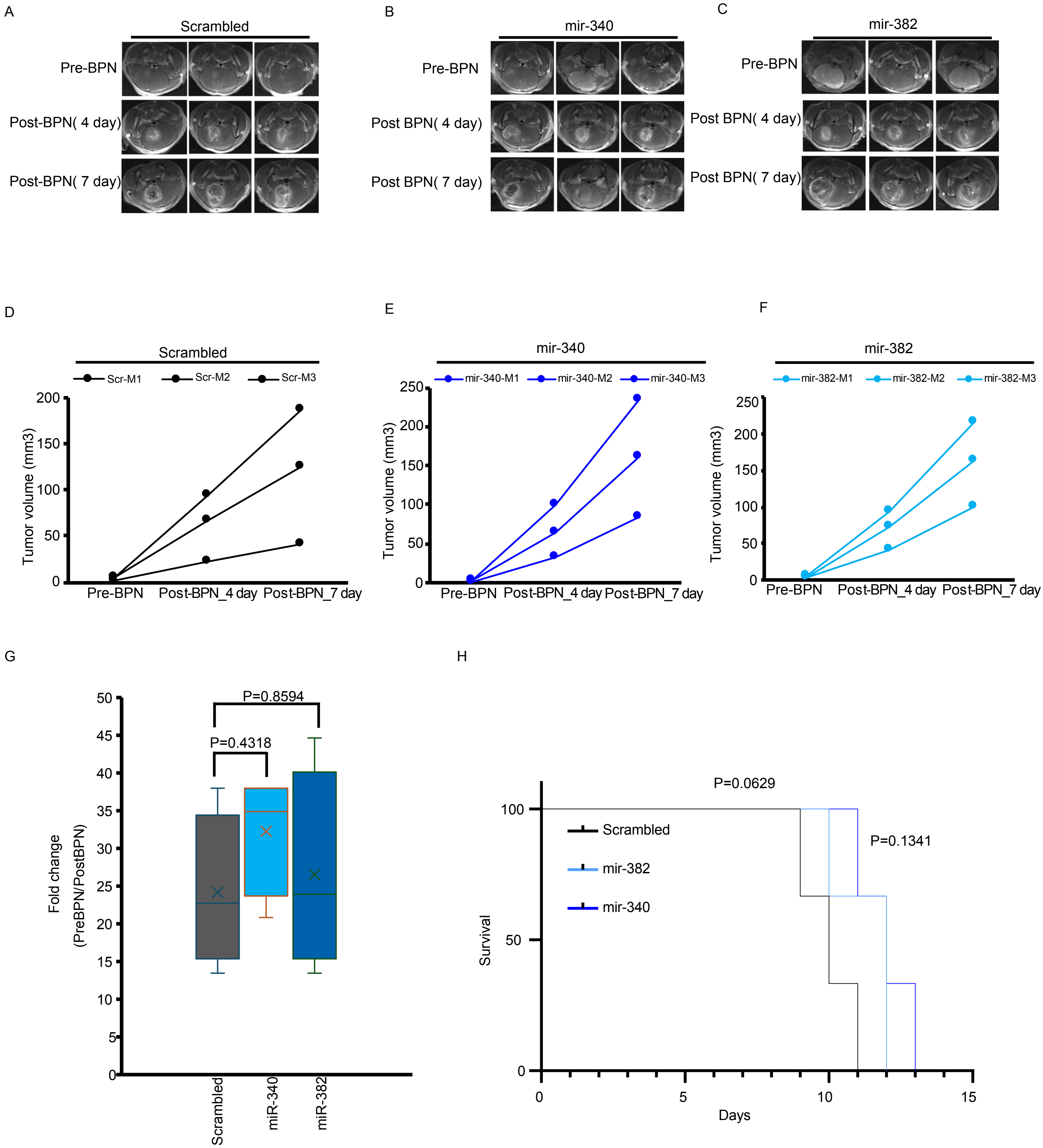

A
Scrambled
Pre-BPN
Post-BPN(4 day)
Post-BPN(7 day)
B
mir-340
Pre-BPN
Post BPN(4 day)
Post BPN(7 day)
C
mir-382
Pre-BPN
Post BPN(4 day)
Post BPN(7 day)
D
Scrambled
Scr-M1
Scr-M2
Scr-M3
Tumor volume (mm3)
0
50
100
150
200
Pre-BPN
Post-BPN_4 day
Post-BPN_7 day
E
mir-340
mir-340-M1
mir-340-M2
mir-340-M3
Tumor volume (mm3)
0
50
100
150
200
250
Pre-BPN
Post-BPN_4 day
Post-BPN_7 day
F
mir-382
mir-382-M1
mir-382-M2
mir-382-M3
Tumor volume (mm3)
0
50
100
150
200
250
Pre-BPN
Post-BPN_4 day
Post-BPN_7 day
G
P=0.8594
P=0.4318
Fold change
(PreBPN/PostBPN)
0
5
10
15
20
25
30
35
40
45
50
Scrambled
miR-340
miR-382
H
P=0.0629
P=0.1341
Scrambled
mir-382
mir-340
Survival
0
50
100
Days
0
5
10
15

Figure S16

A

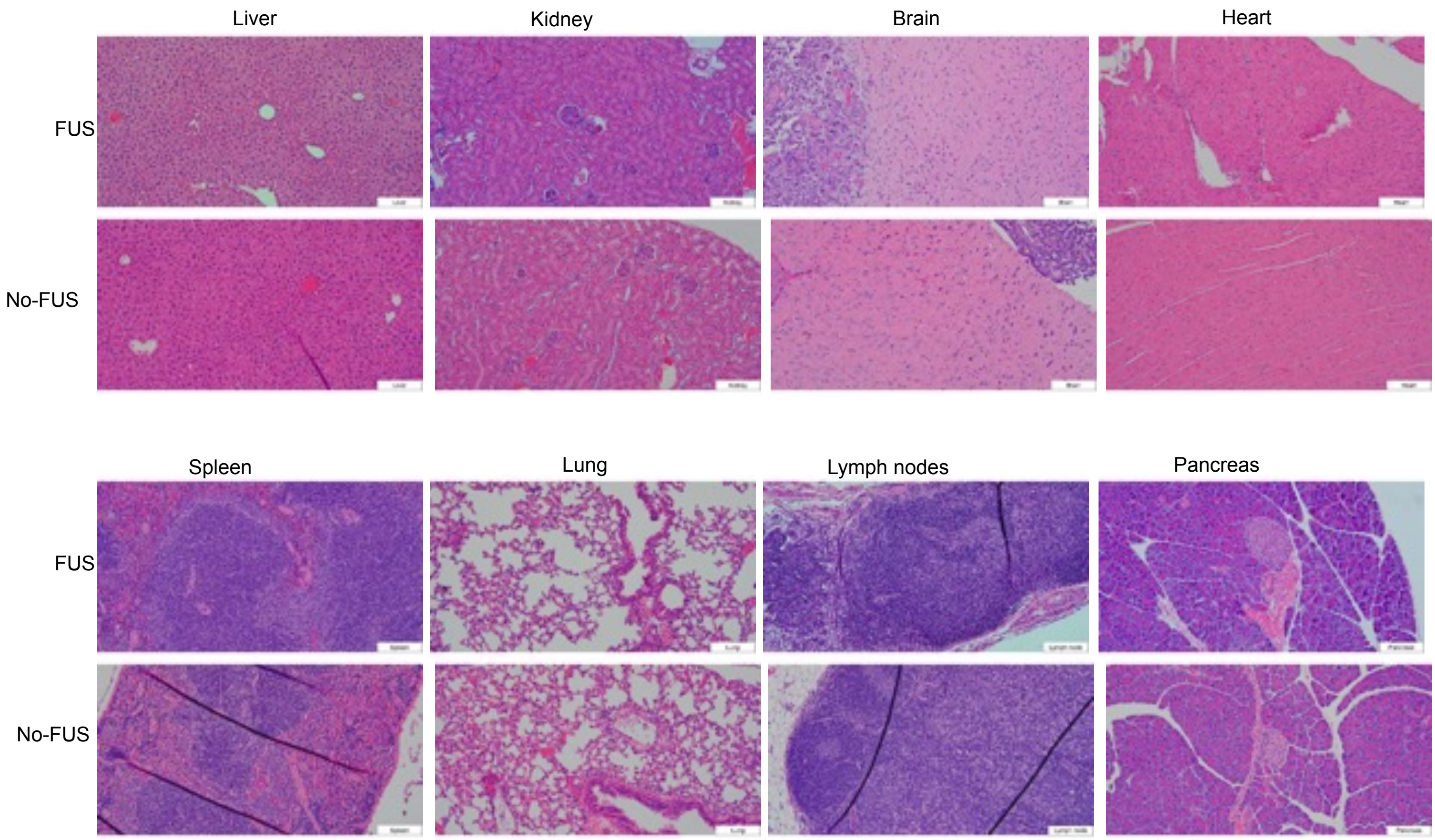

B

Organ	FUS	No FUS
Pancreas	No injury	No injury
Lymph nodes	No injury	No injury
Kidneys	No injury	No injury
Liver	No injury	No injury
Spleen	No injury	No injury
Heart	No injury	No injury
Lungs	No injury	No injury
Brain	Tumor, no injury	Tumor, no injury

Table S8. Physicochemical properties of PEG-PBAE particles.

NP	Hydrodynamic diameter[a] ±SD(nm)	PDI[a]±SD	ζ-potential[lb]±SD(mv)
PEG-PBABE/miR-control	70.2±4.7	0.16±0.03	2.8±0.8
PEG-PBABE/miR-382	71.7±1.2	0.14±0.02	2.3±0.6
PEG-PBABE/miR-340	70.7±1.0	0.12±0.02	1.8±0.7

aHydrodynamic diameter (number mean) and polydispersity index (PDI) were measured by dynamic light scattering (DLS) in 10 mM NaCl at pH 7.4. Mean ±SD(n=4). bζ-potential was measured by laser doppler anemometry in 10 mM NaCl at pH 7.4. Mean ±SD(n=4)
