# Supplementary material for: Therapeutic delivery of microRNAs discovered to target deregulated glioblastoma pathways inhibits tumor growth in mice
Source: J Clin Invest. 2026 Jun 30;136(16):e195639. doi: 10.1172/JCI195639 (PMC13476142; doi:10.1172/JCI195639)
Supplement: Supplementary file 2 [file jci-136-195639-s010.pdf]

Figure 2A

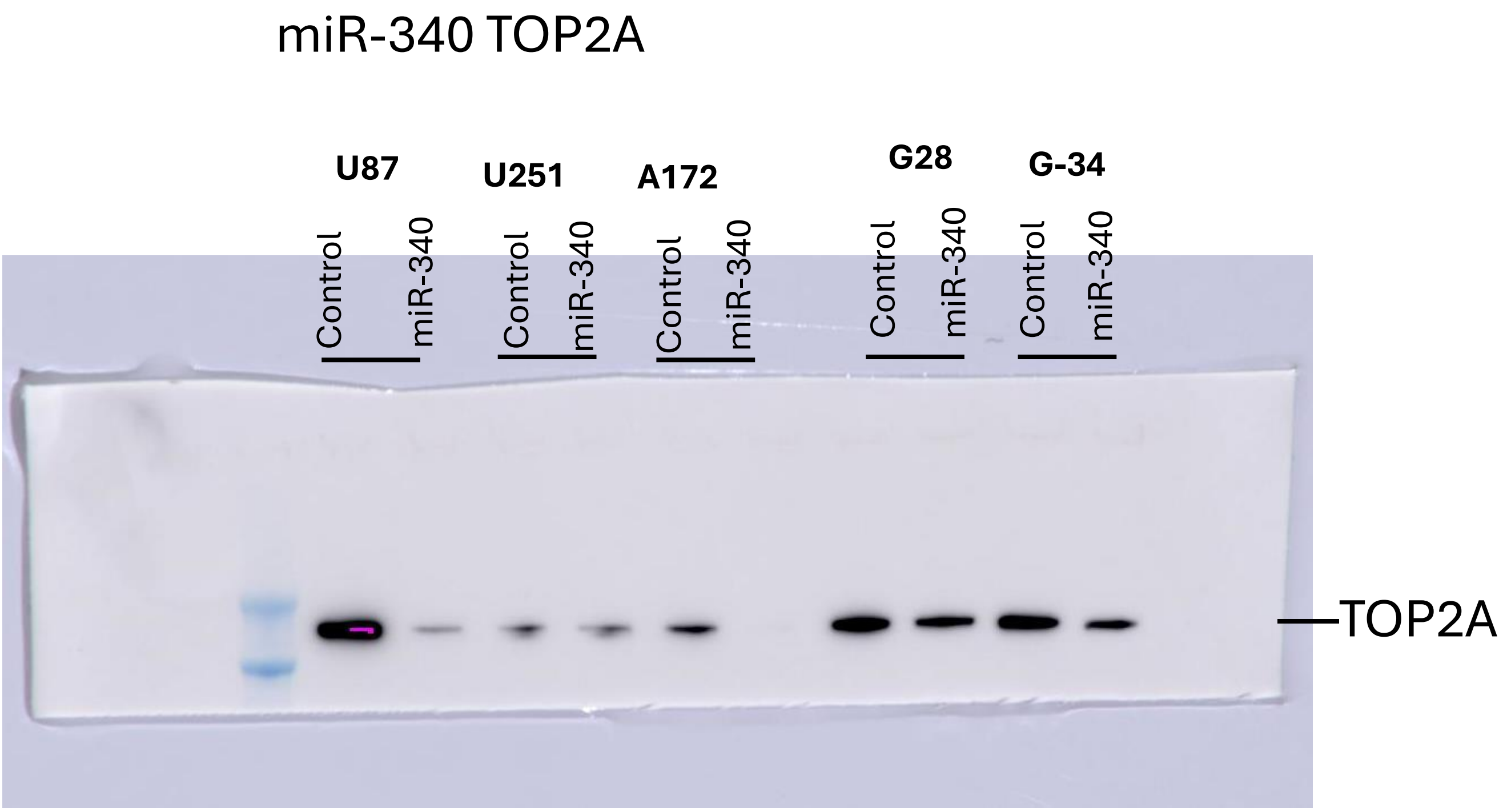
miR-340 TOP2A
U87
U251
A172
G28
G-34
Control
miR-340
Control
miR-340
Control
miR-340
Control
miR-340
Control
miR-340
TOP2A

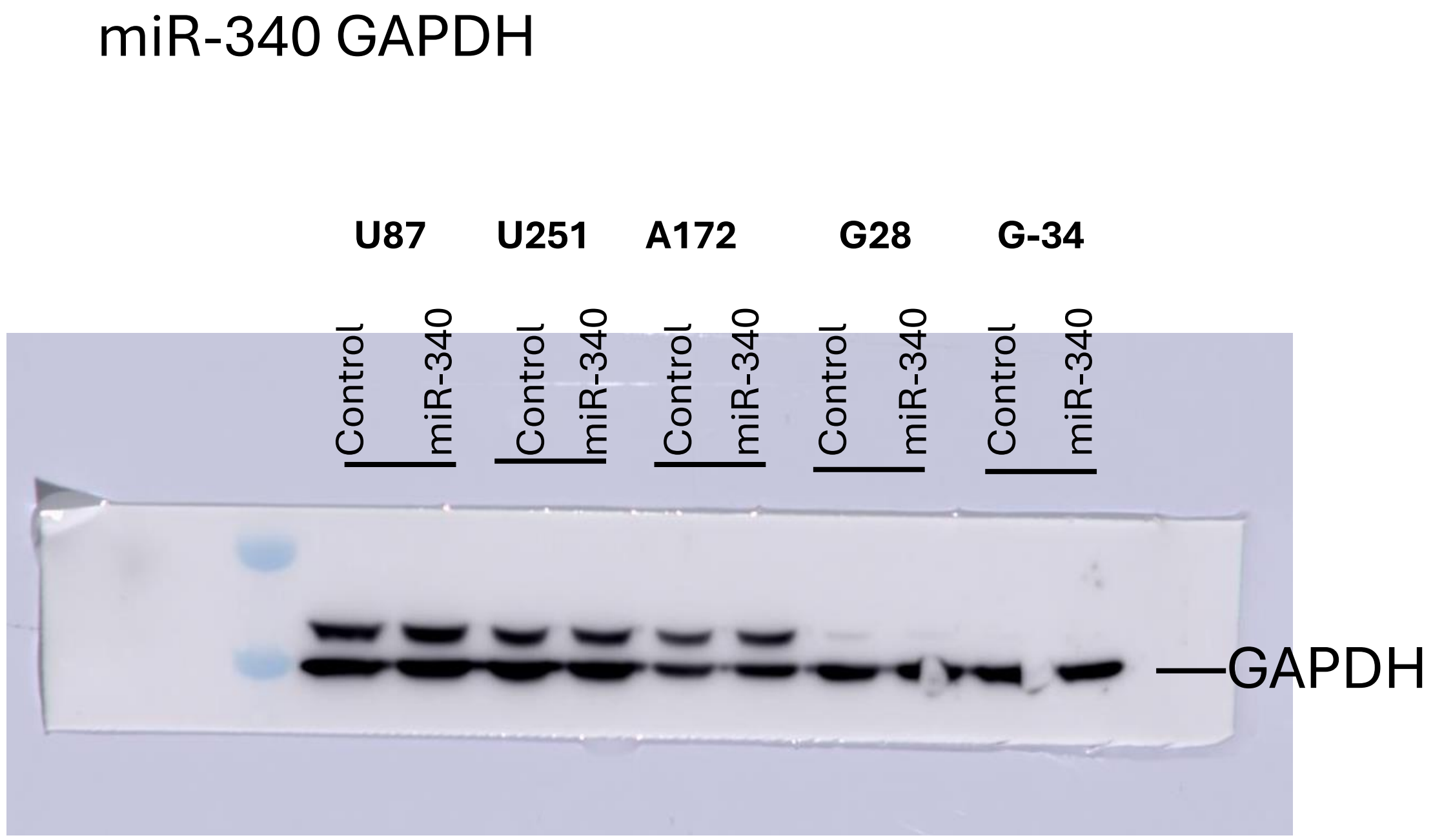
miR-340 GAPDH
U87
U251
A172
G28
G-34
Control
miR-340
Control
miR-340
Control
miR-340
Control
miR-340
Control
miR-340
GAPDH

Figure 2B

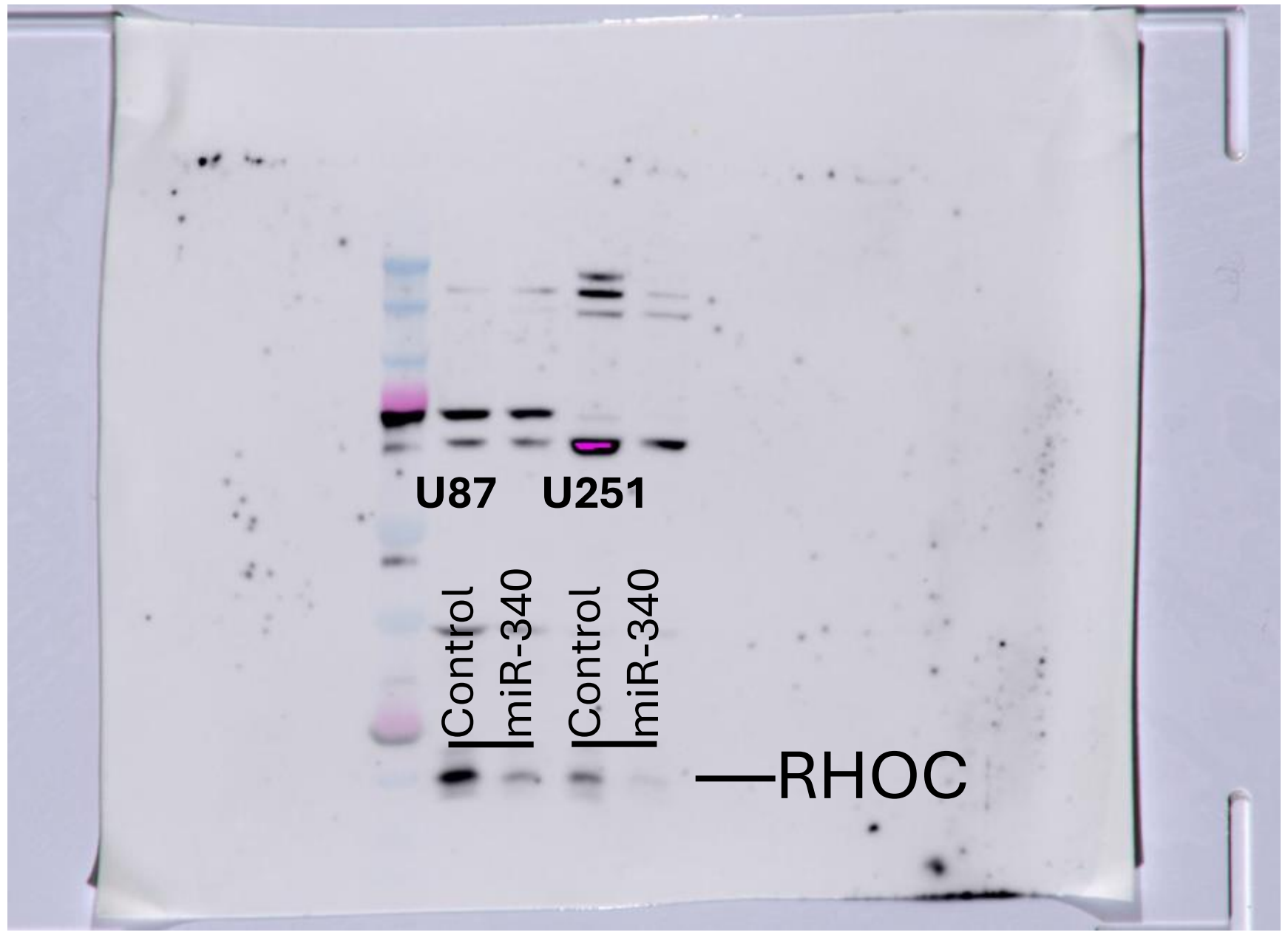

Figure 2B

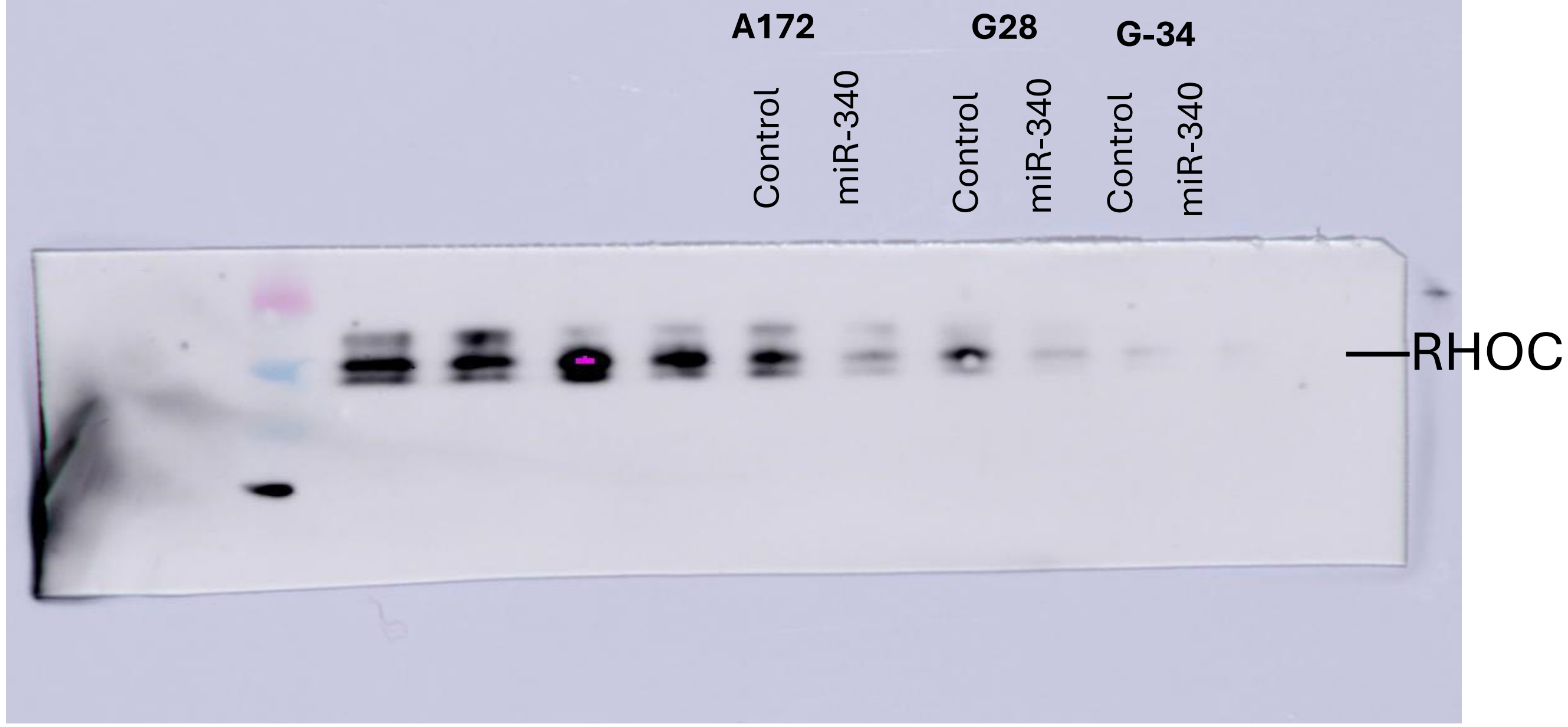
A172
G28
G-34
Control
miR-340
Control
miR-340
Control
miR-340
RHOC

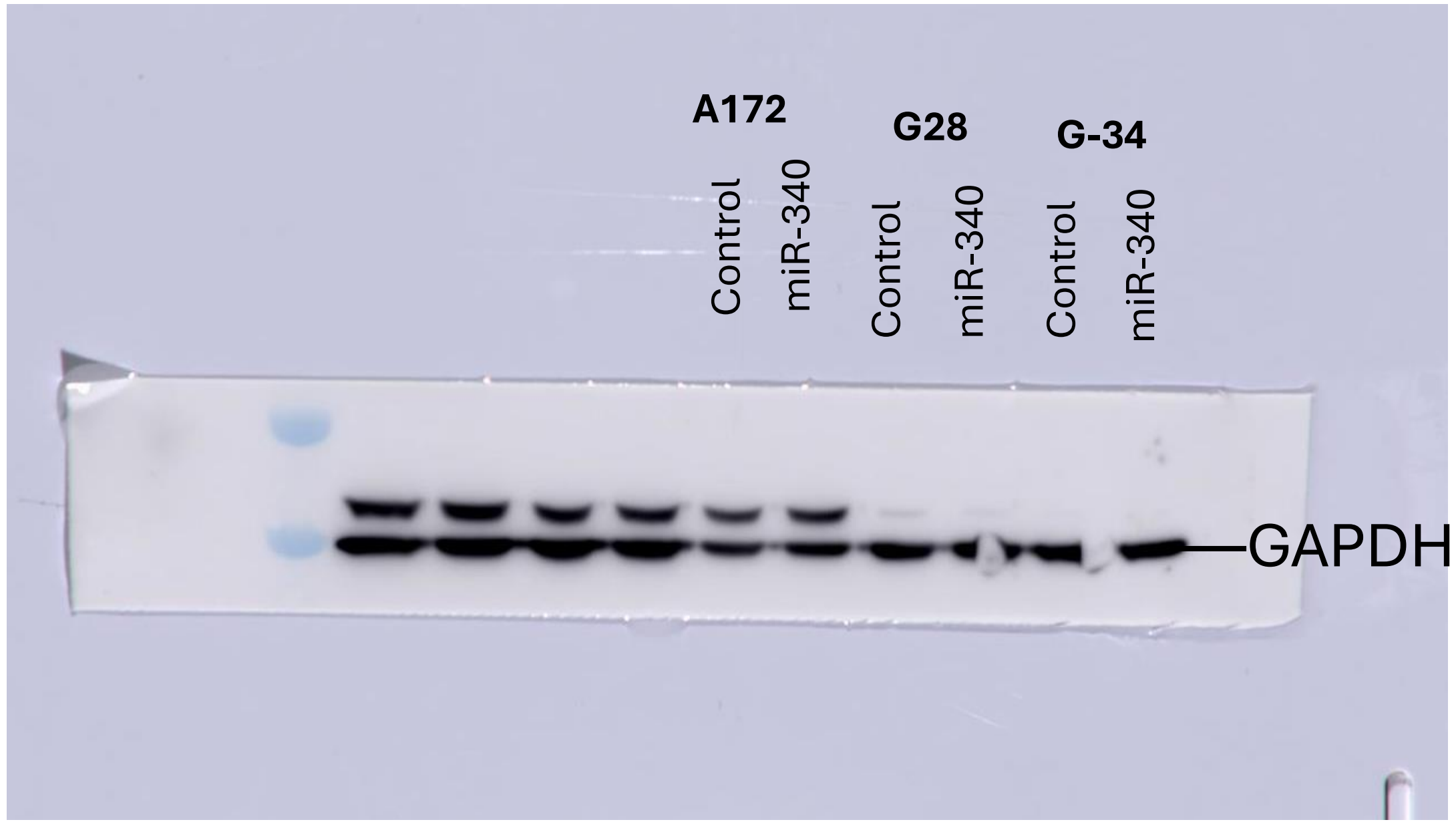
A172
G28
G-34
Control
miR-340
Control
miR-340
Control
miR-340
GAPDH

Figure 2C

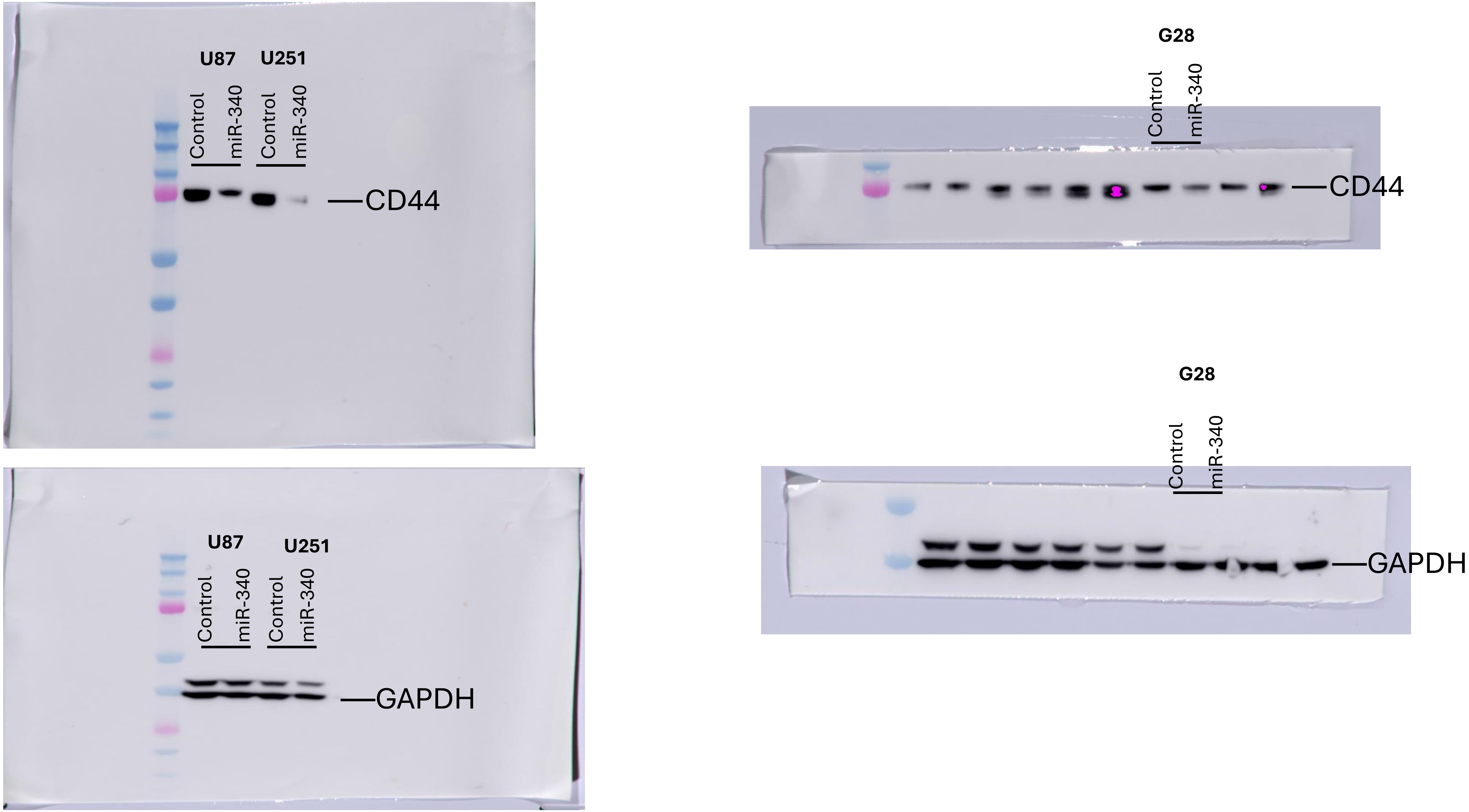
U87
U251
Control
miR-340
Control
miR-340
CD44
U87
U251
Control
miR-340
Control
miR-340
GAPDH
G28
Control
miR-340
CD44
G28
Control
miR-340
GAPDH

Figure 2D

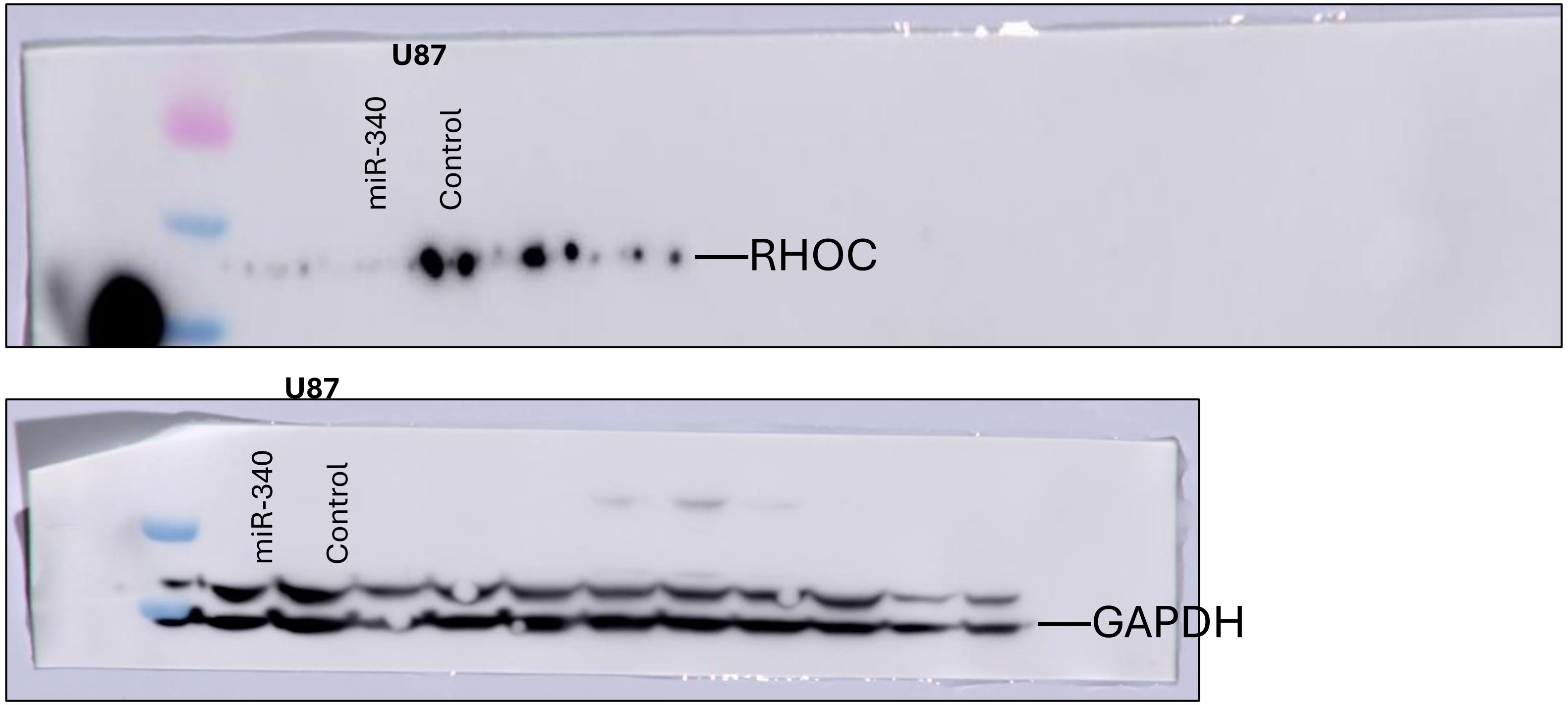

Figure 2E

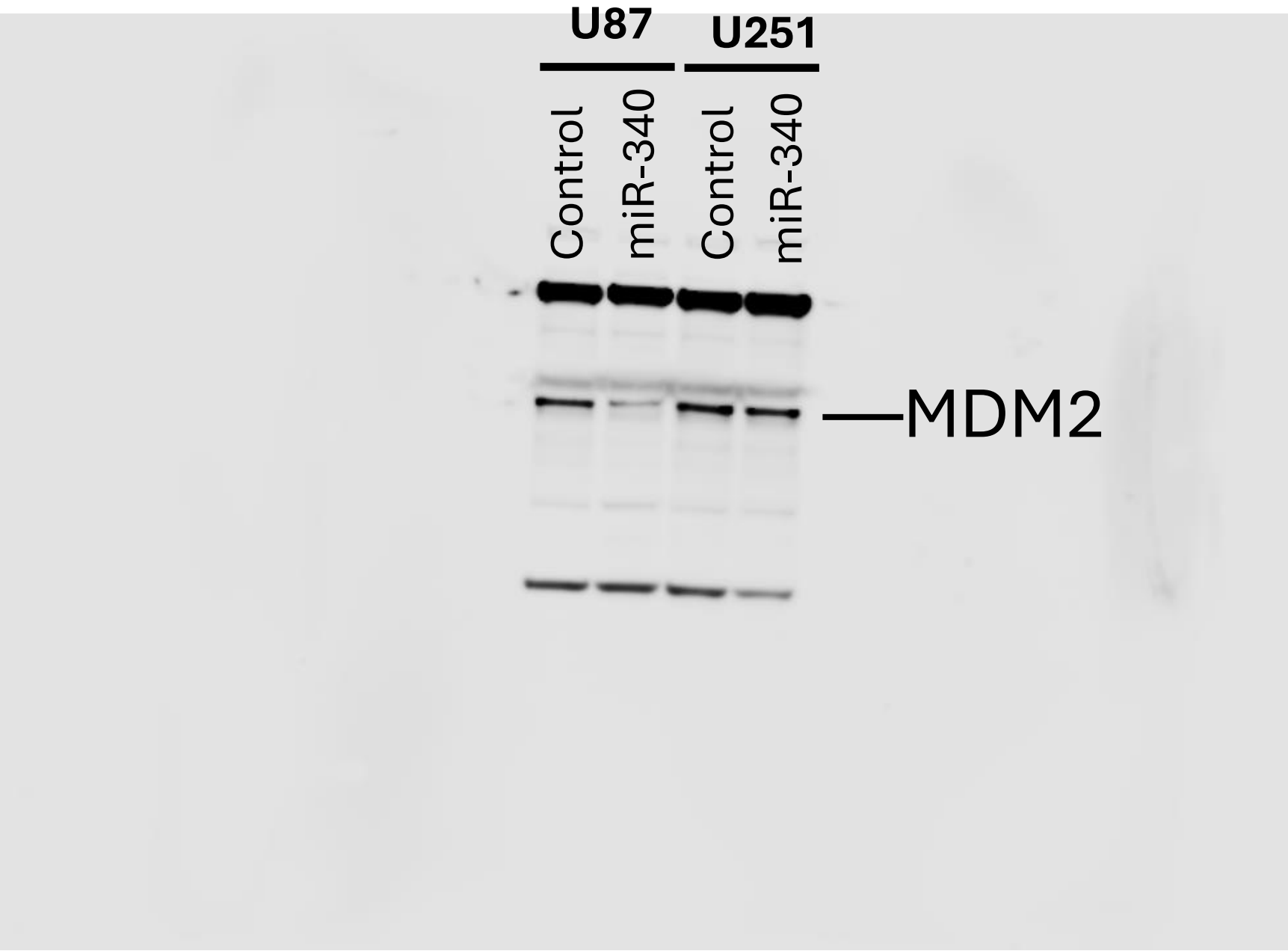

U87
U251
Control
miR-340
Control
miR-340
MDM2

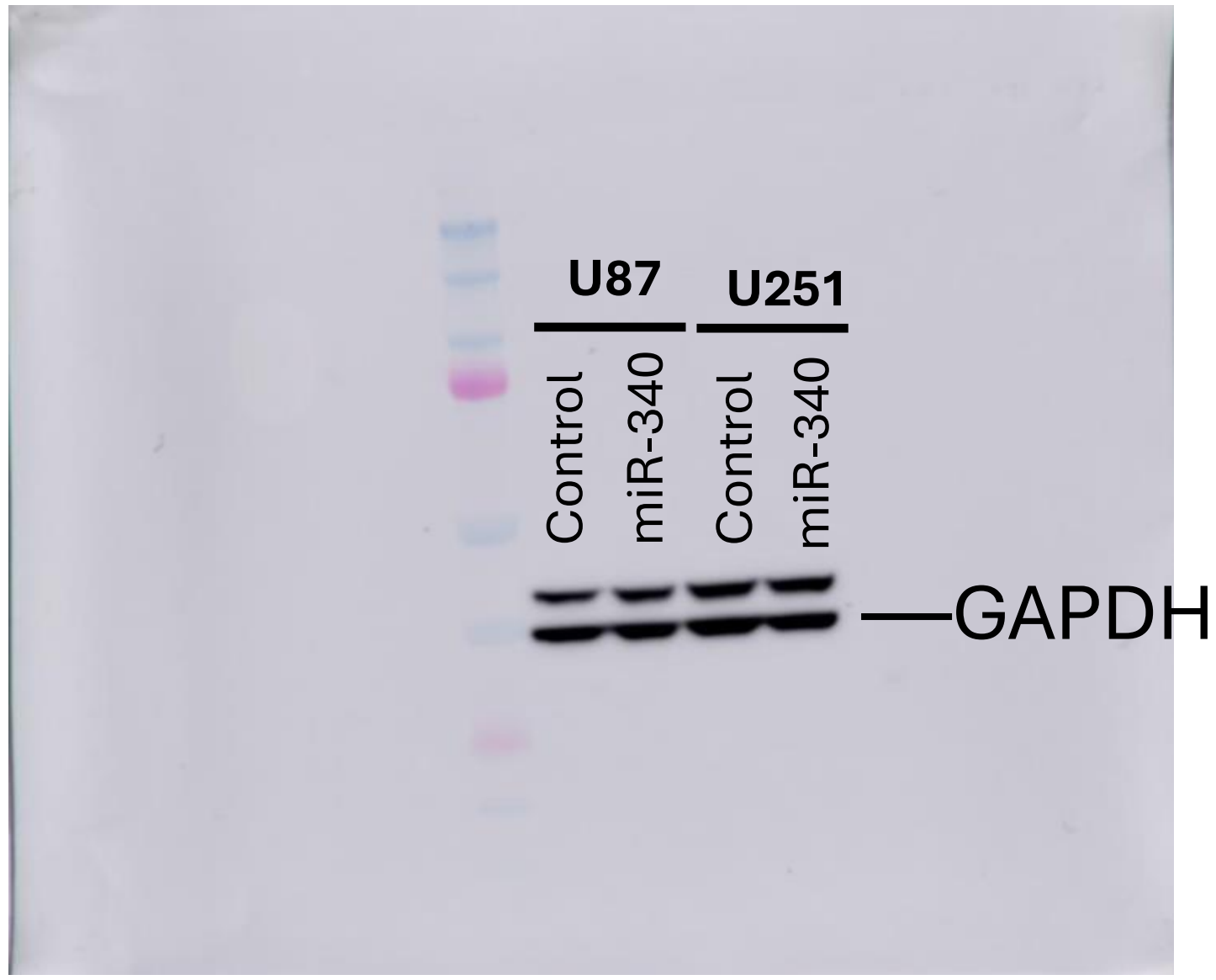

U87
U251
Control
miR-340
Control
miR-340
GAPDH

Figure 2E

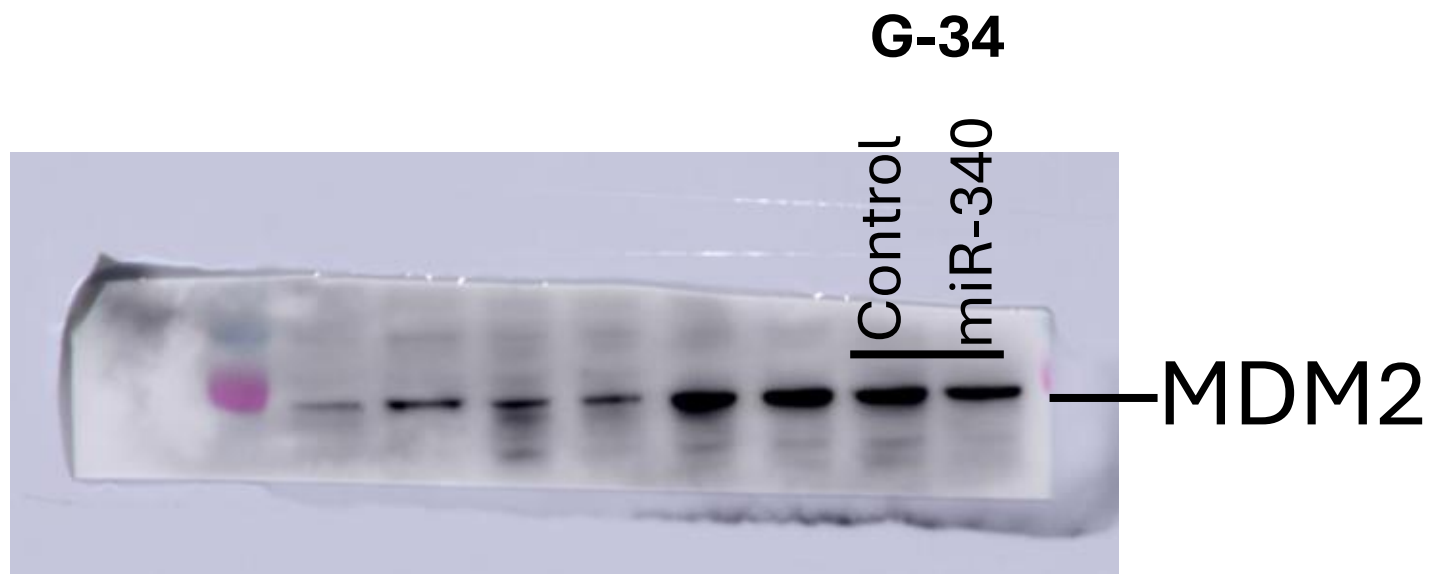

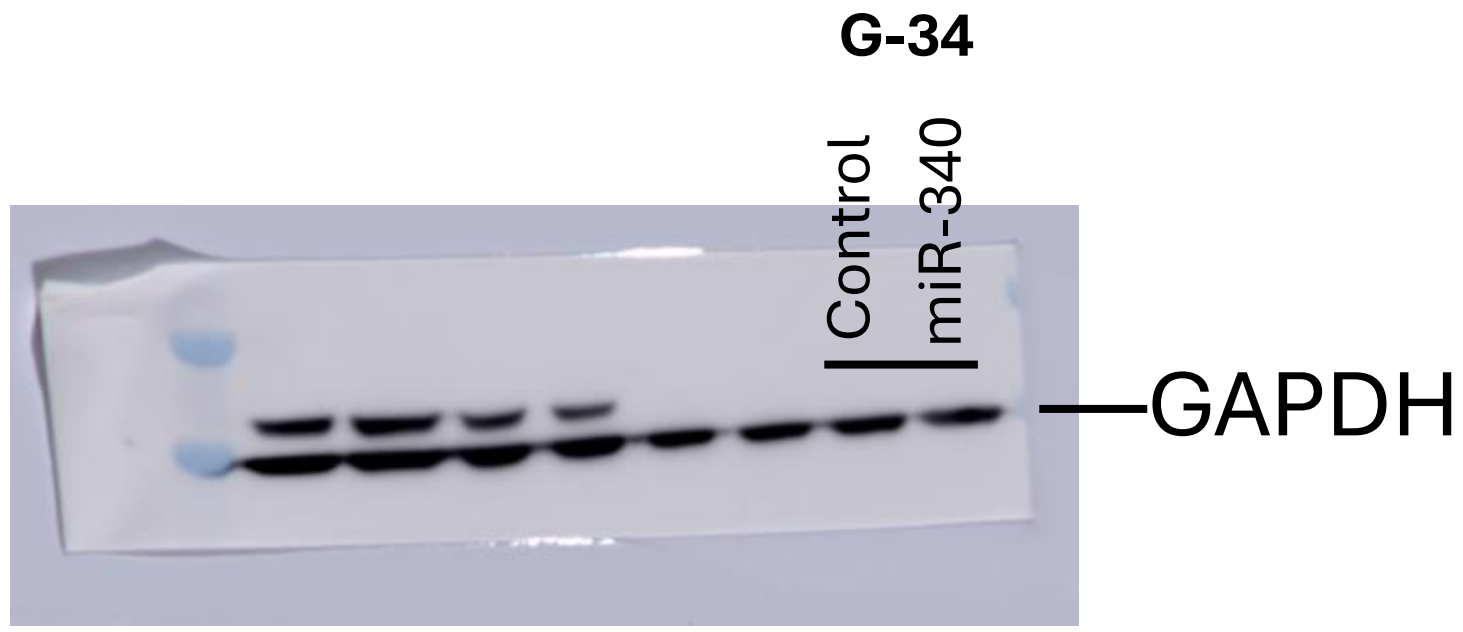

Figure 2F

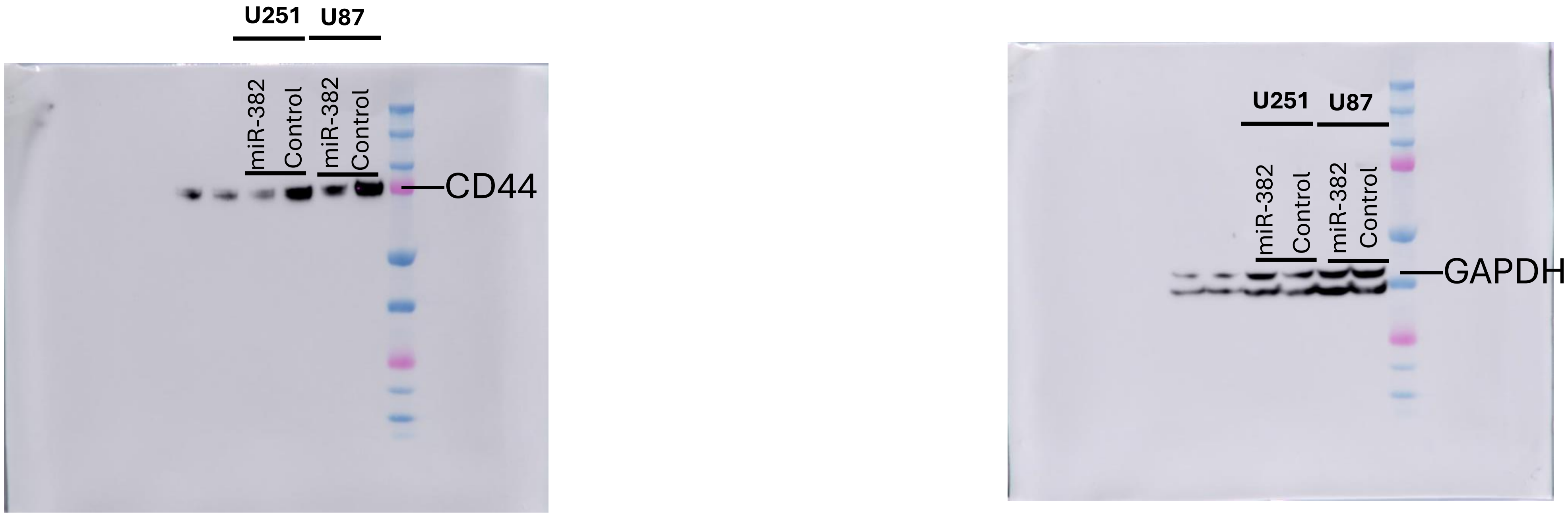
U251
U87
miR-382
Control
miR-382
Control
CD44
U251
U87
miR-382
Control
miR-382
Control
GAPDH

Figure 2G

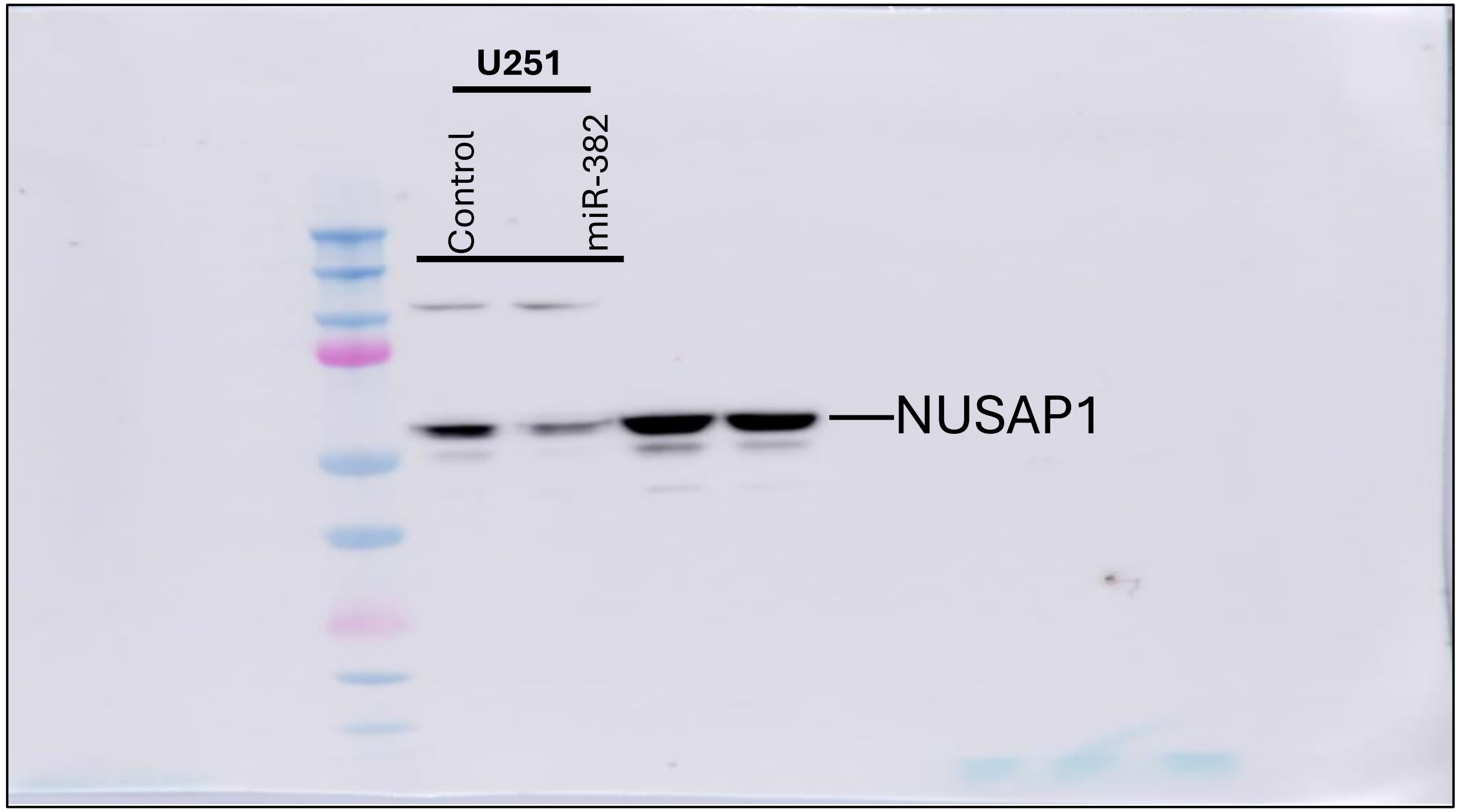

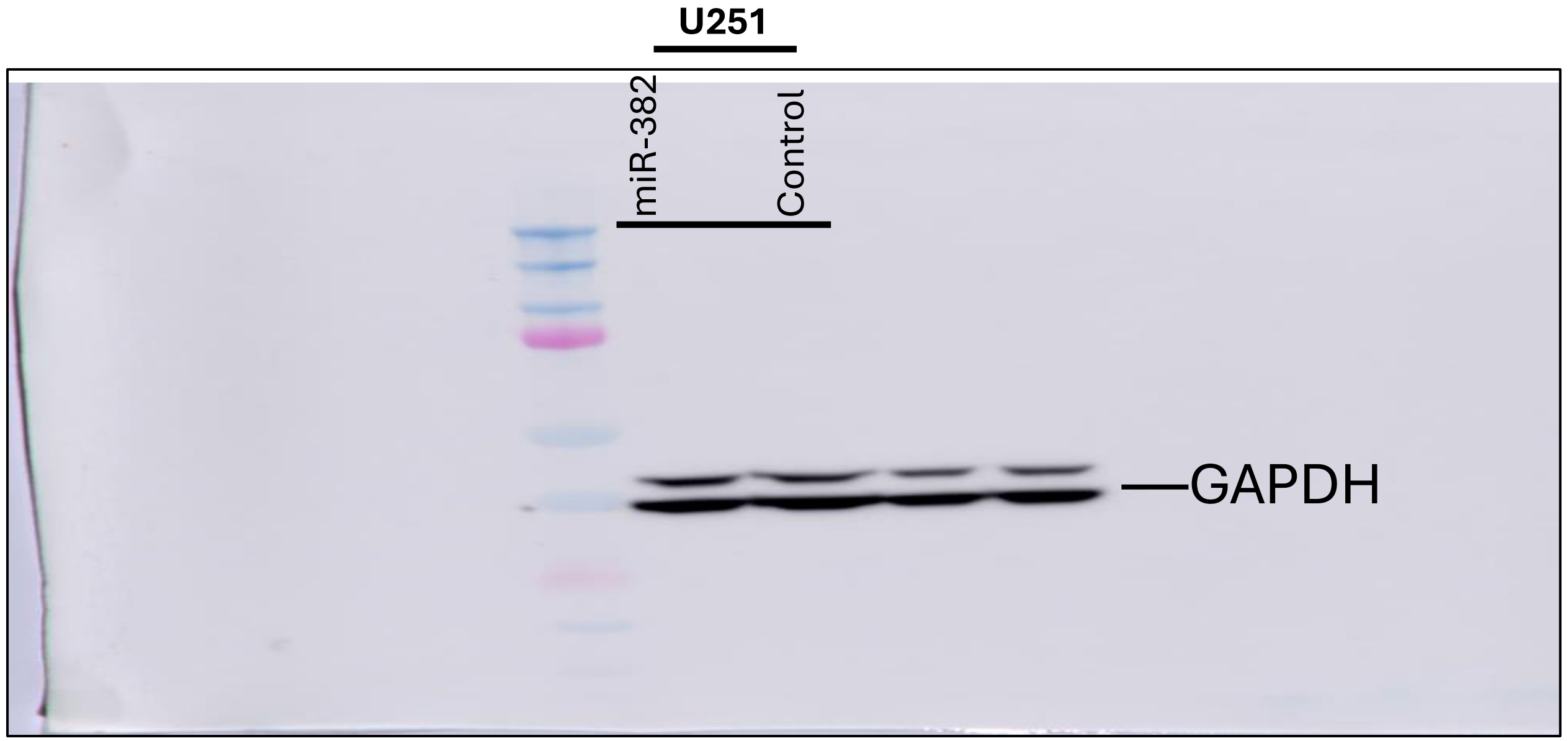

Figure 2G

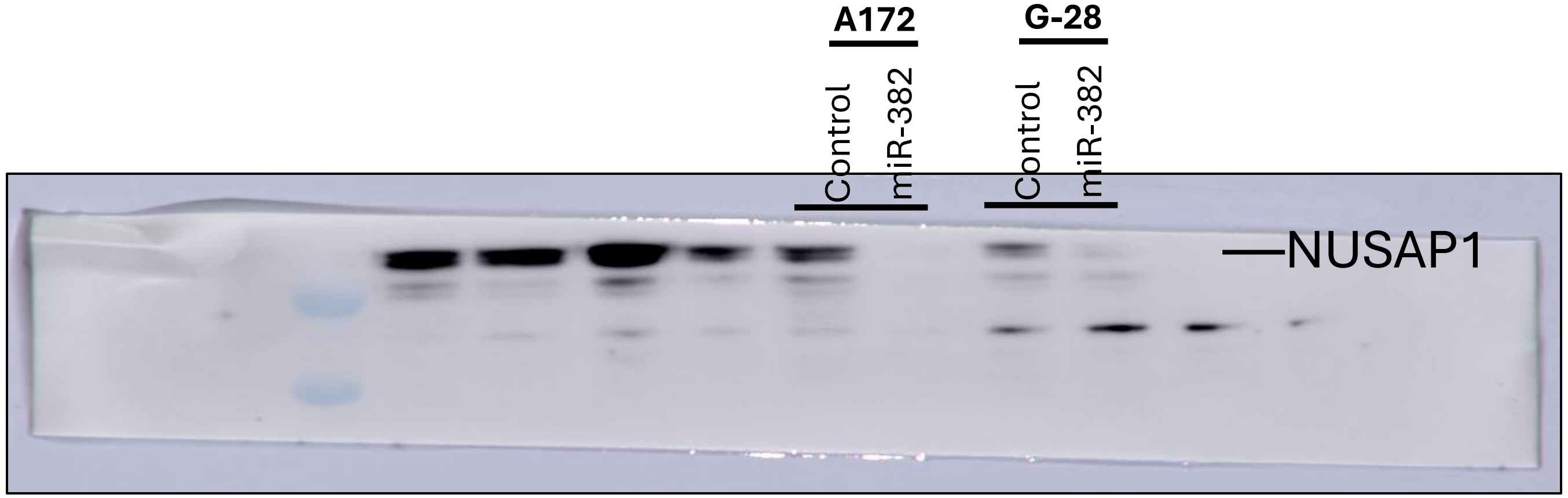
A172
G-28
Control
miR-382
Control
miR-382
NUSAP1

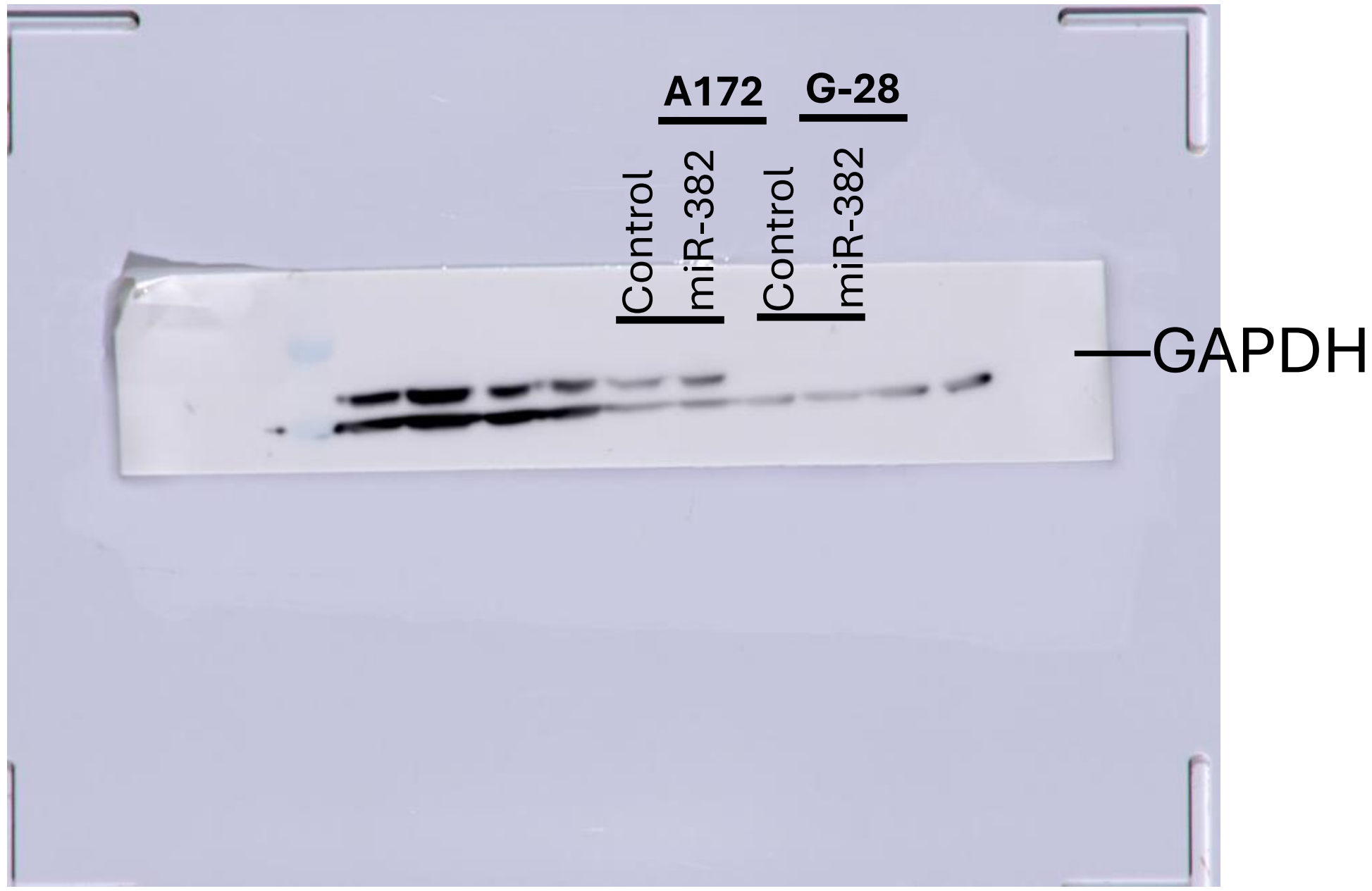
A172
G-28
Control
miR-382
Control
miR-382
GAPDH

Figure 2H

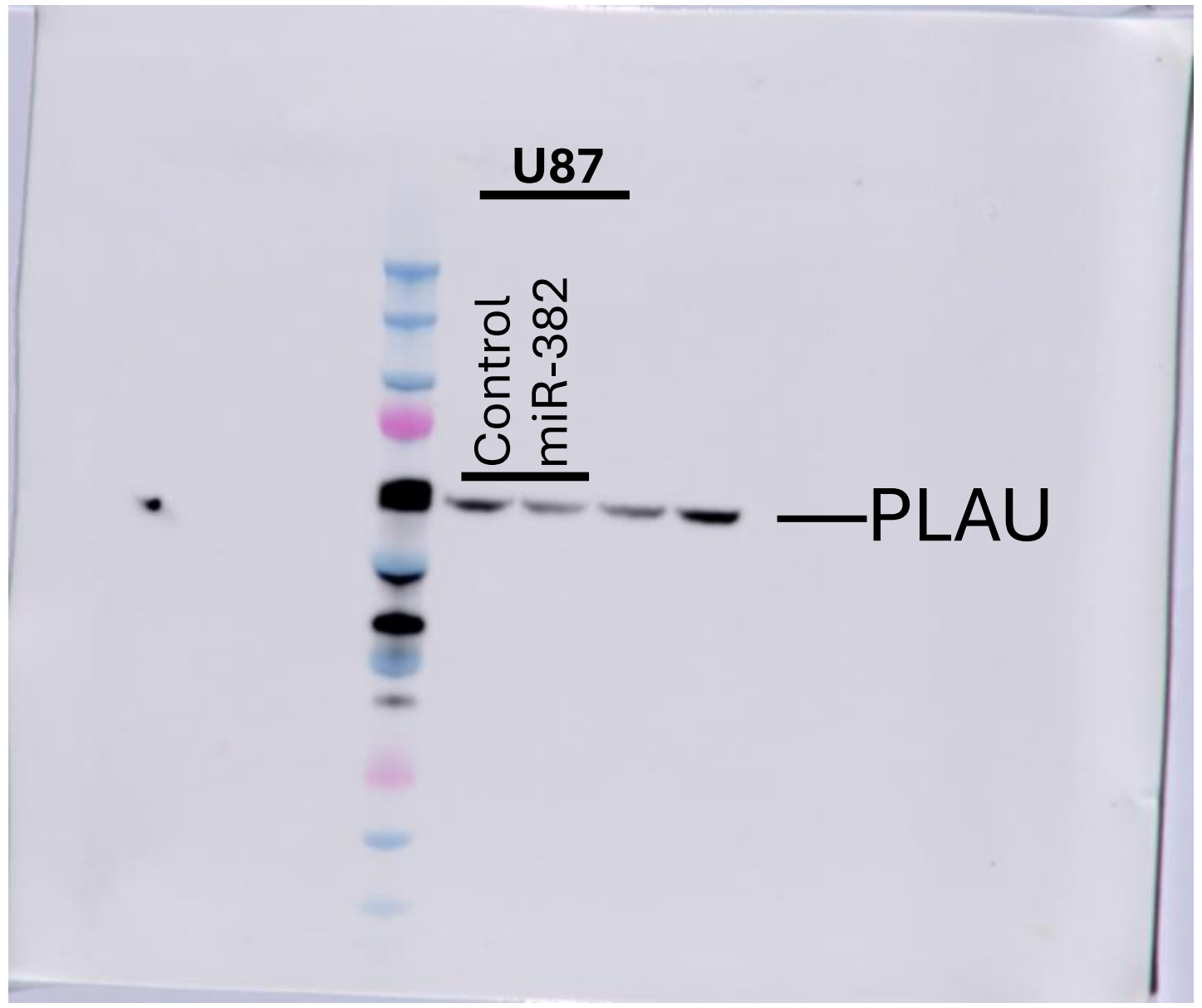

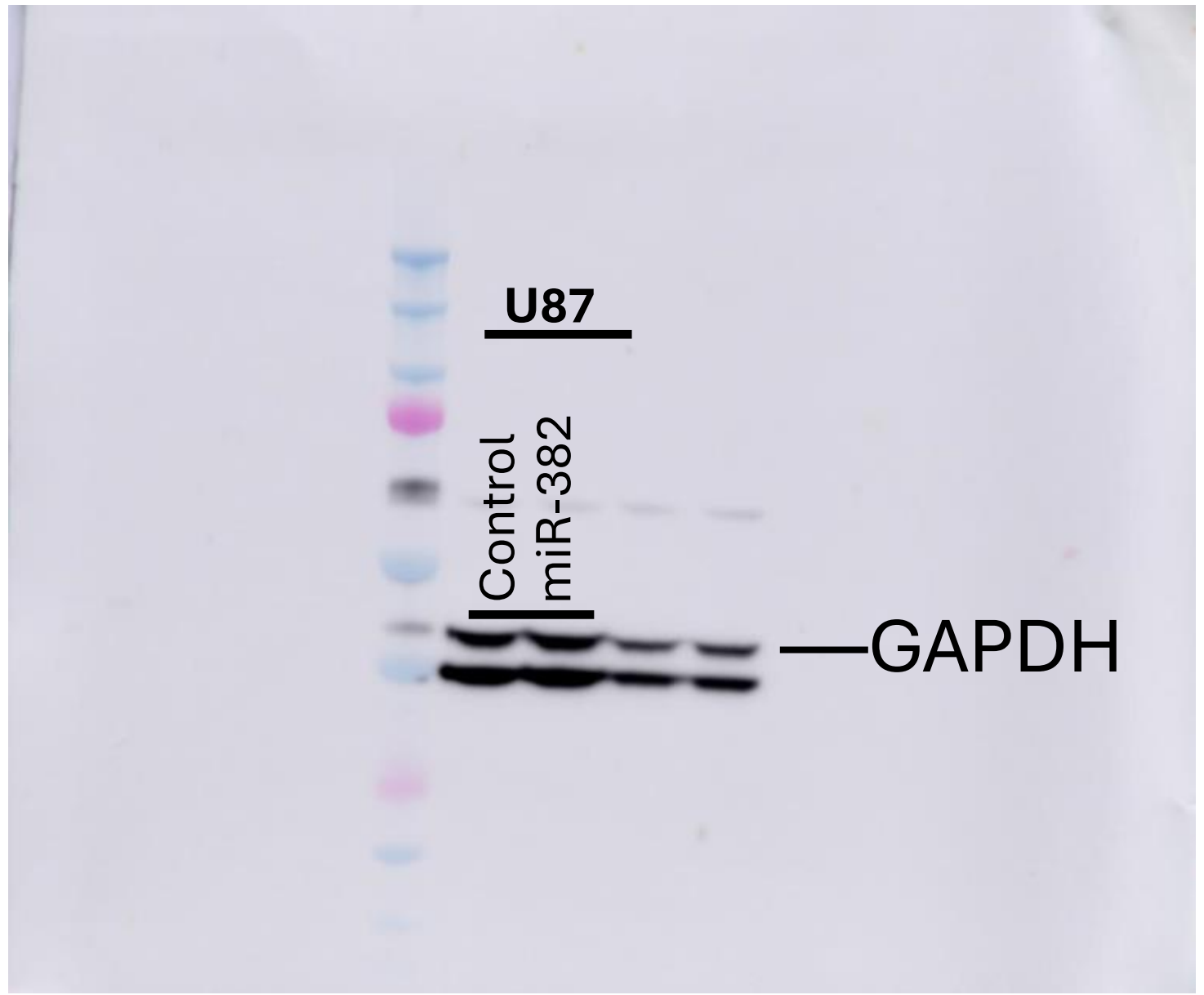

Figure 2I

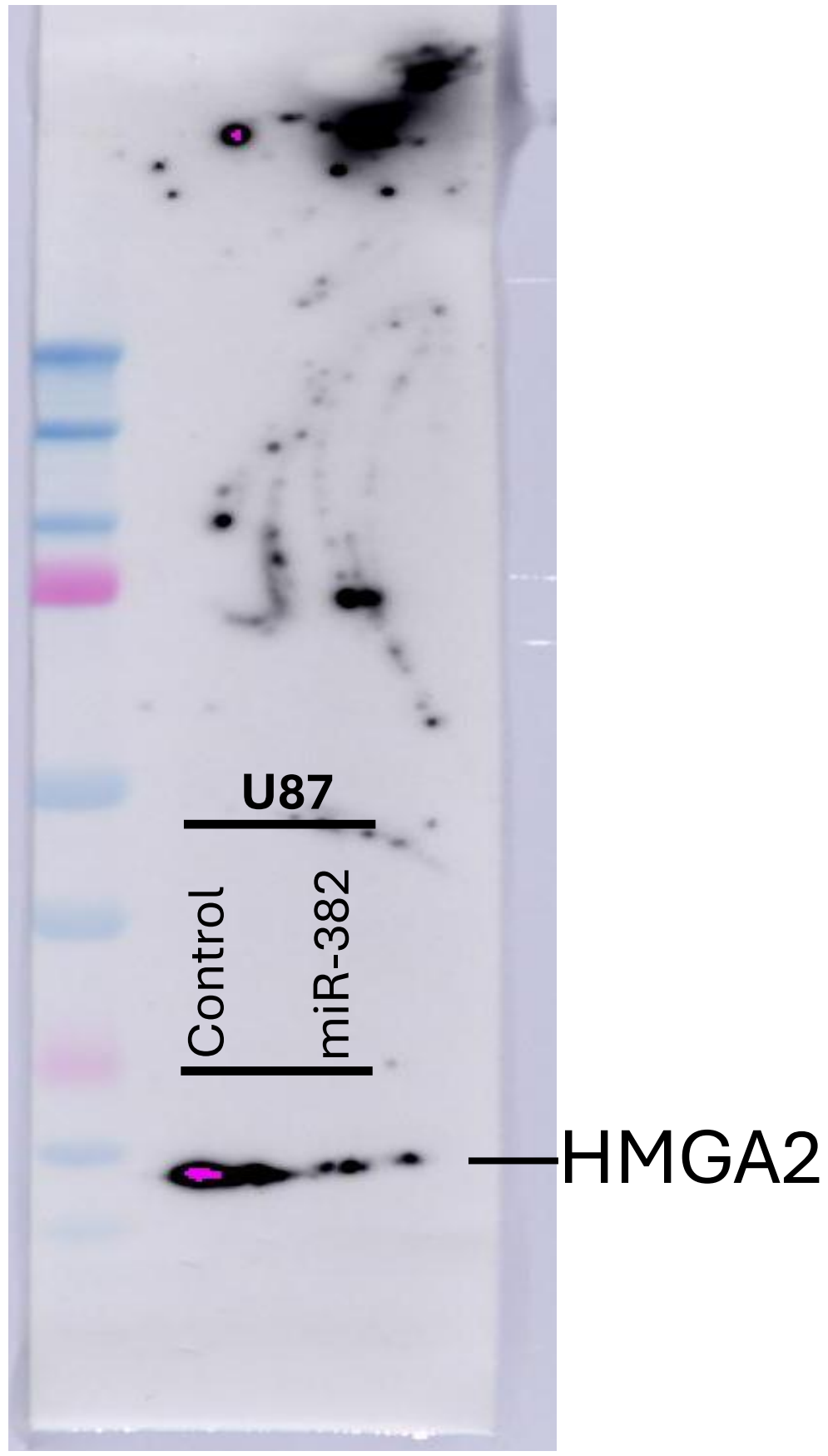

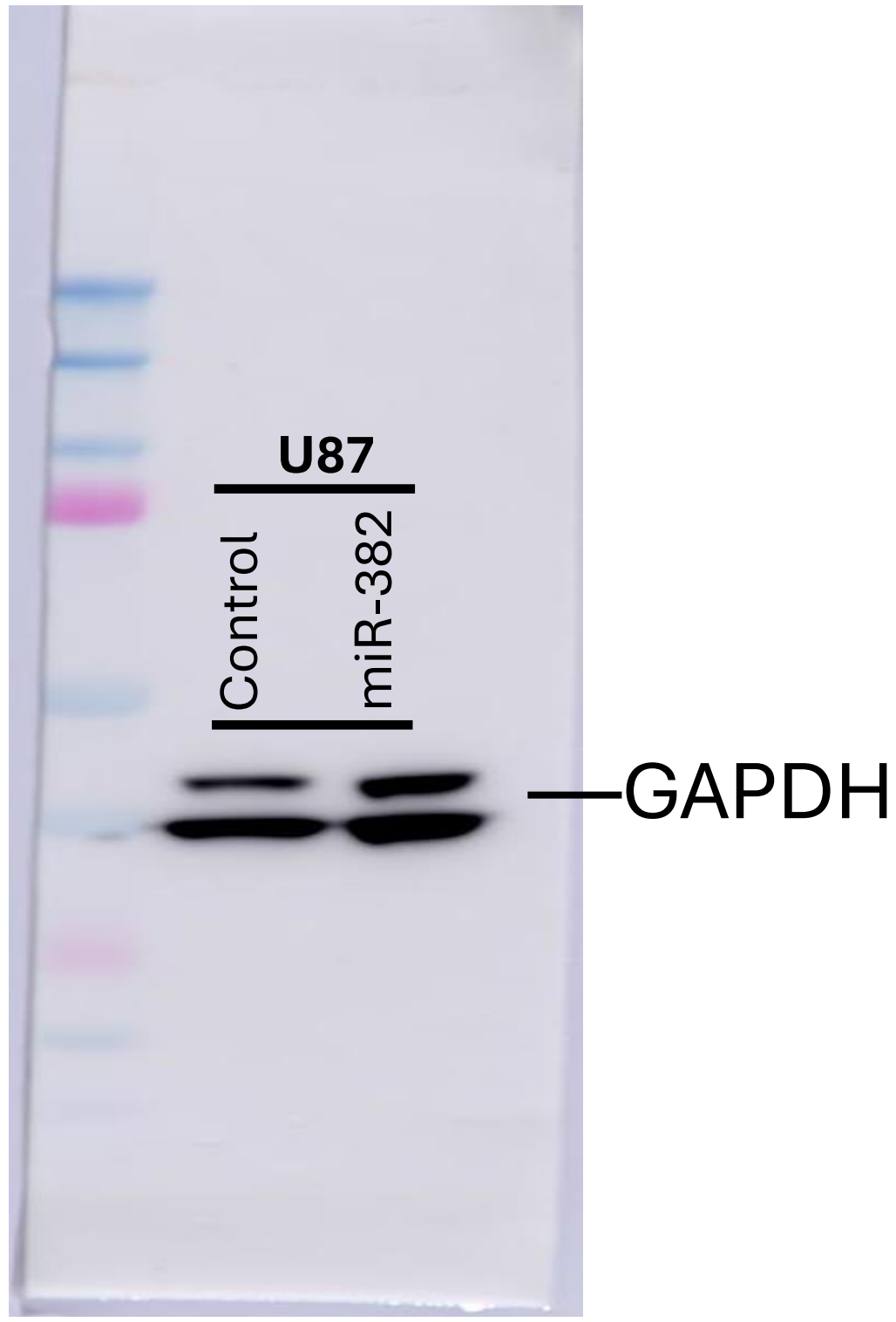

Figure 3A-B

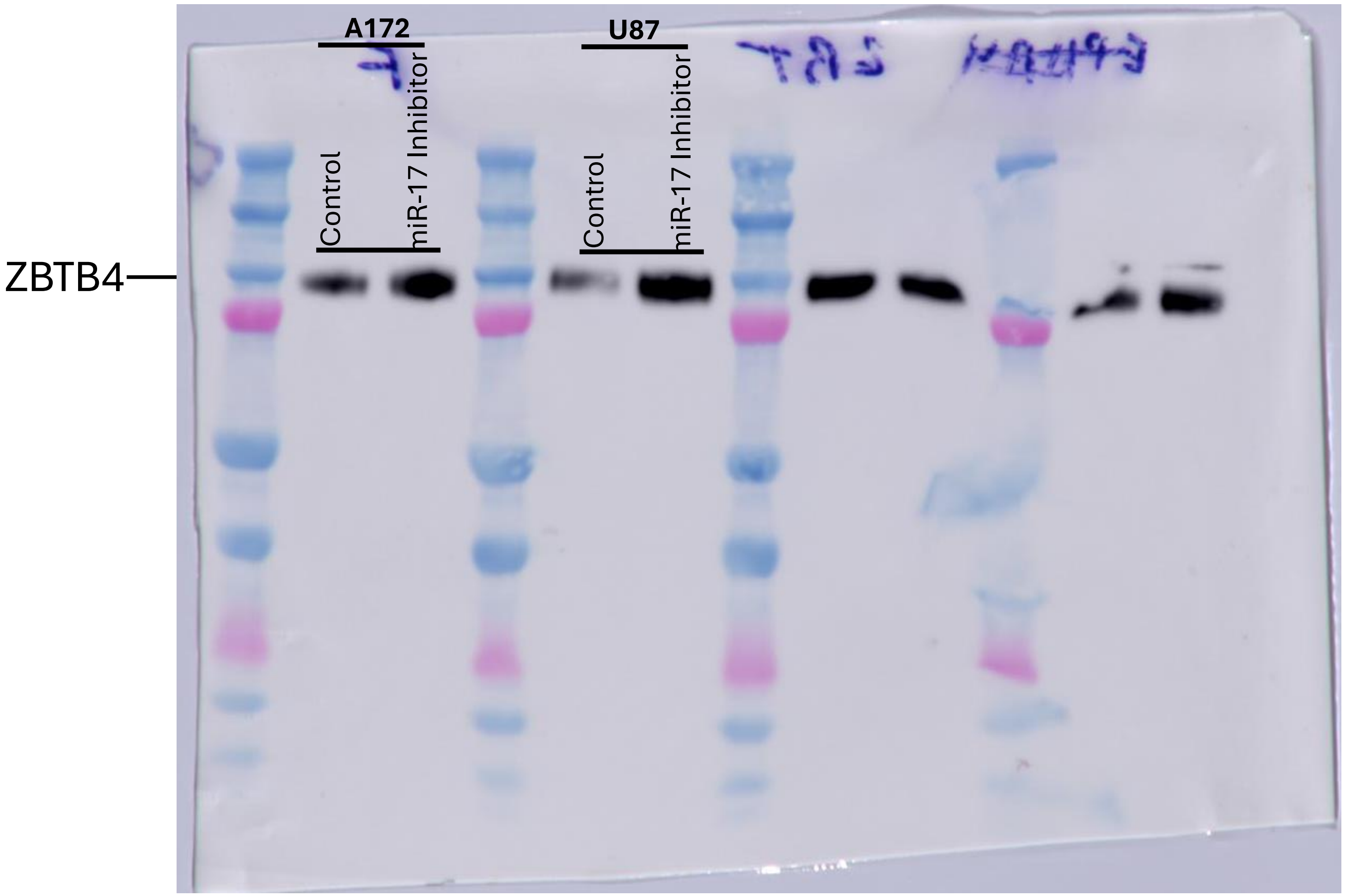
A172
U87
Control
miR-17 Inhibitor
Control
miR-17 Inhibitor
ZBTB4

Figure 3A-B

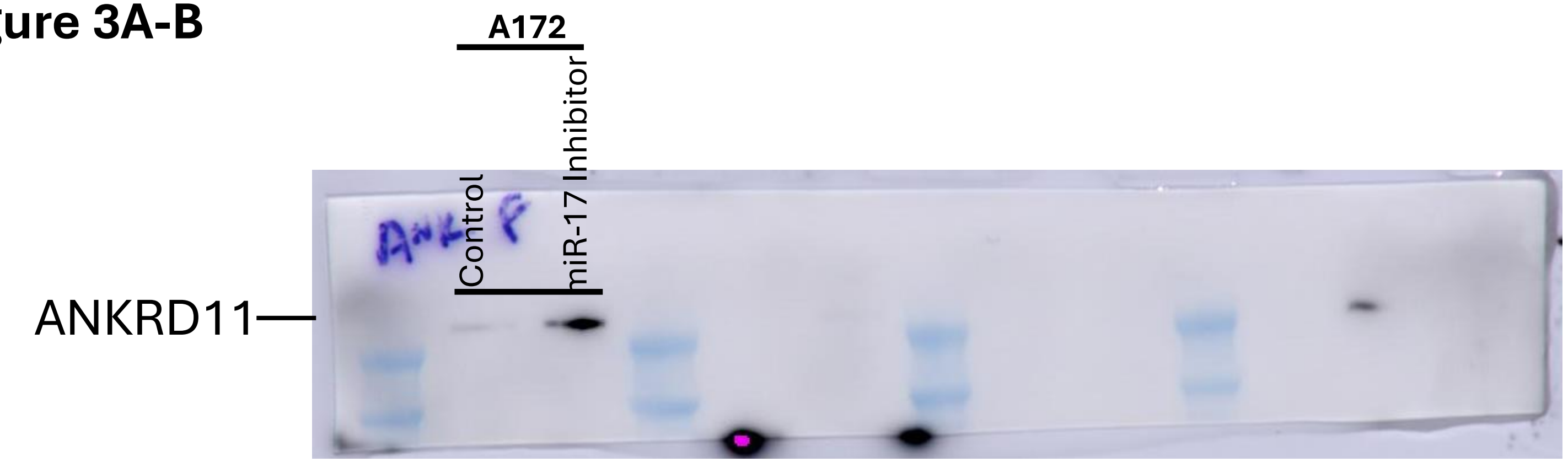
A172
Control
miR-17 Inhibitor
ANKRD11

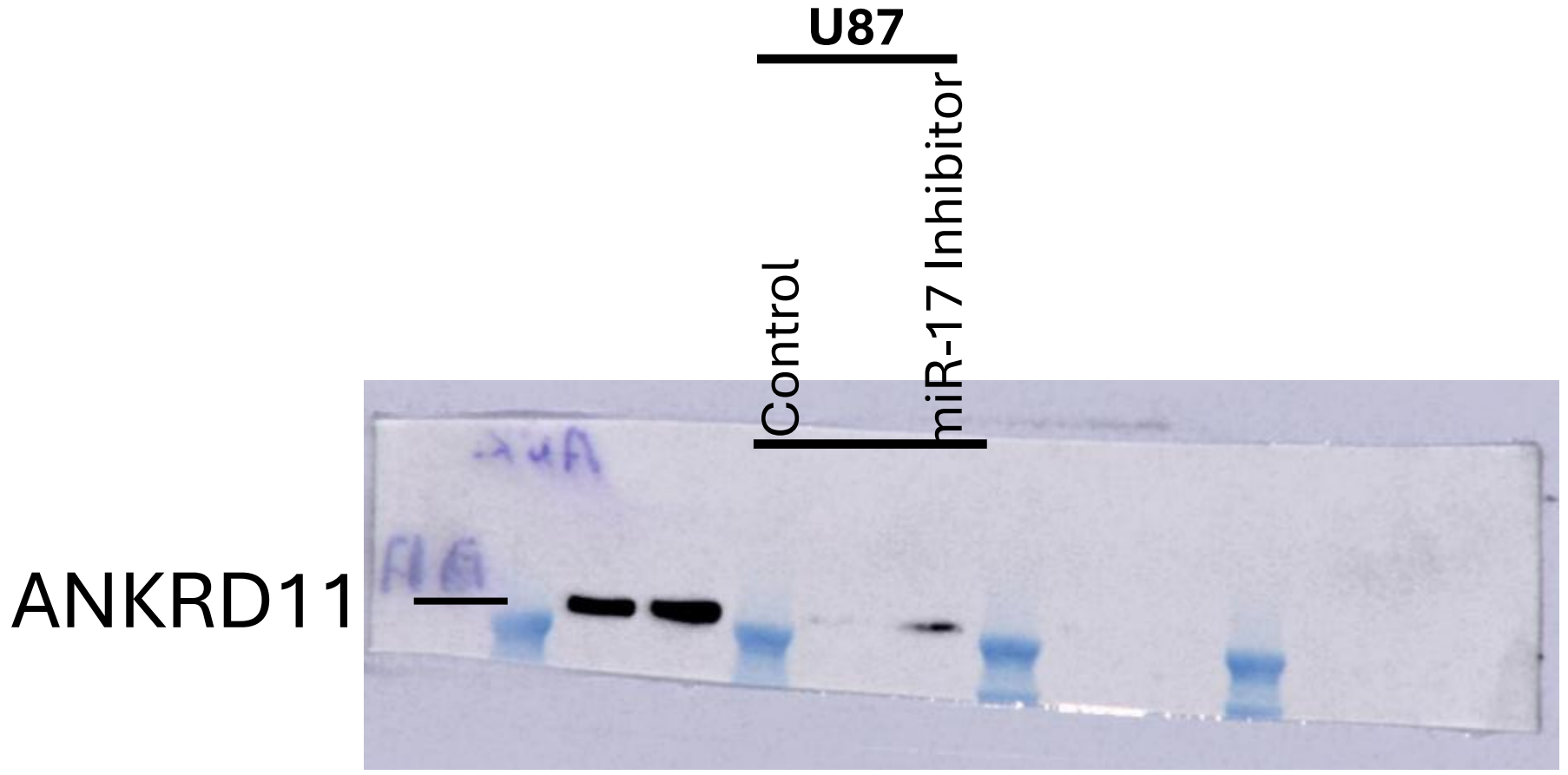
U87
Control
miR-17 Inhibitor
ANKRD11

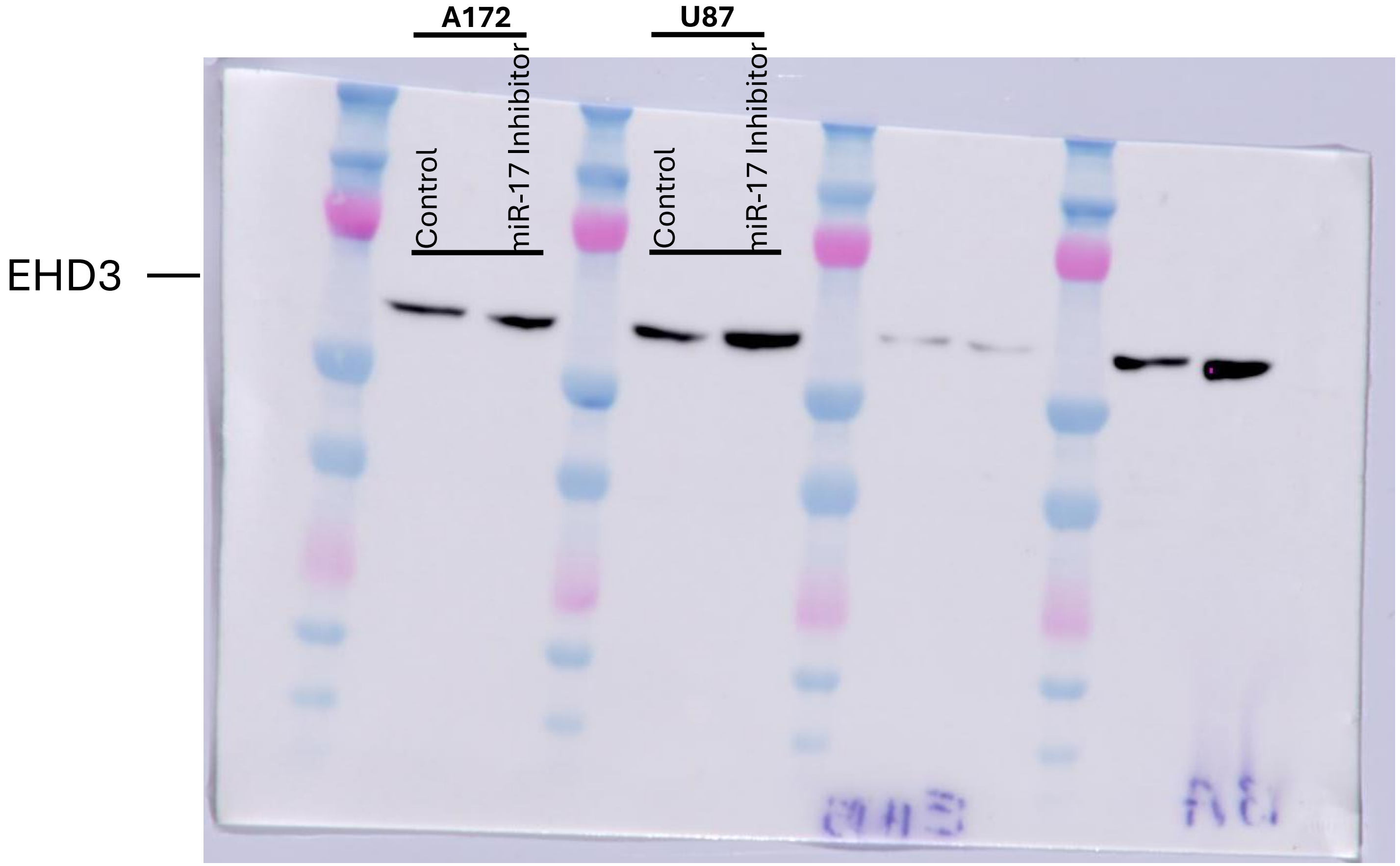
A172
U87
Control
miR-17 Inhibitor
Control
miR-17 Inhibitor
EHD3

Figure 3A-B

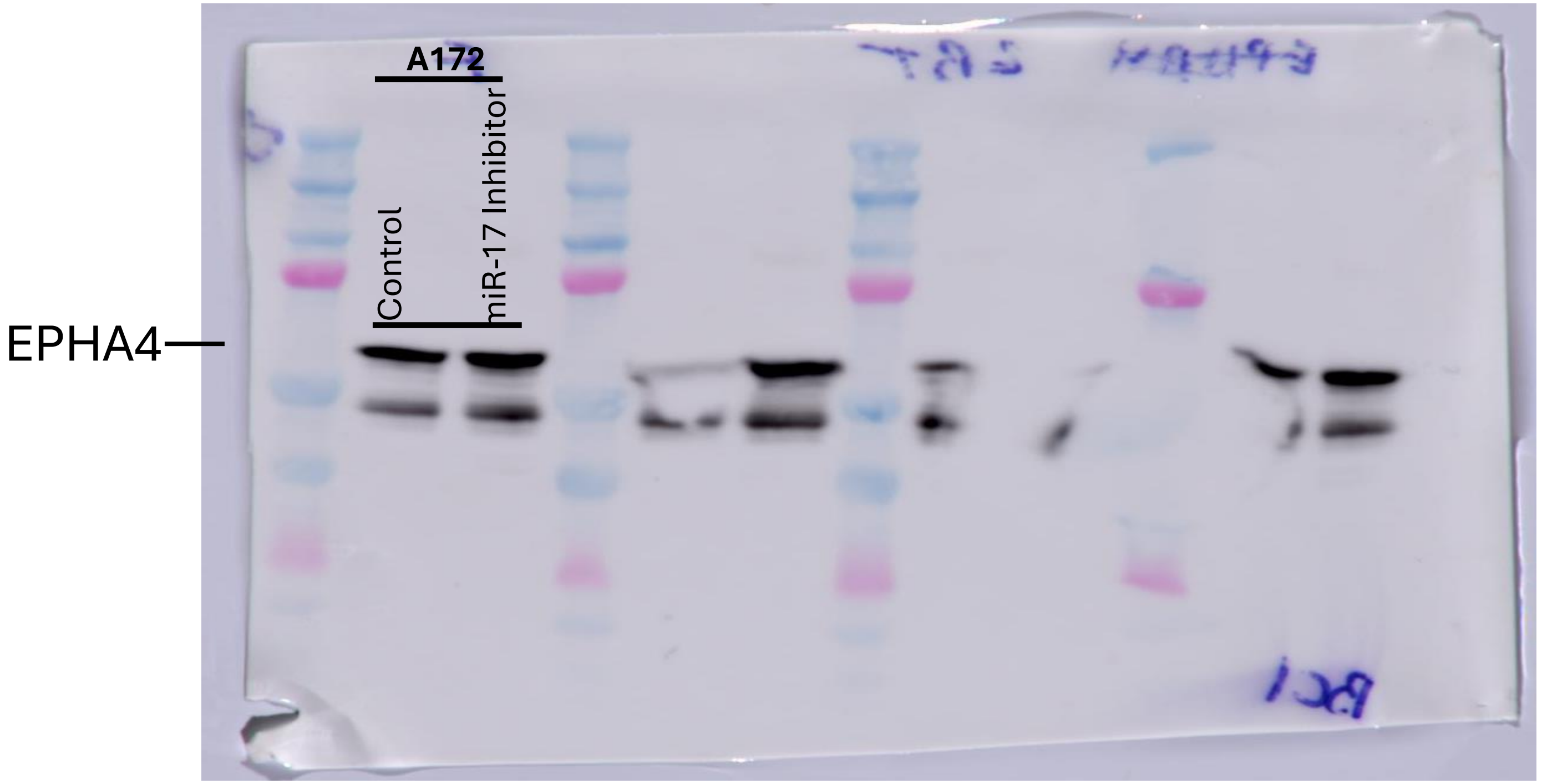

Figure 3A-B

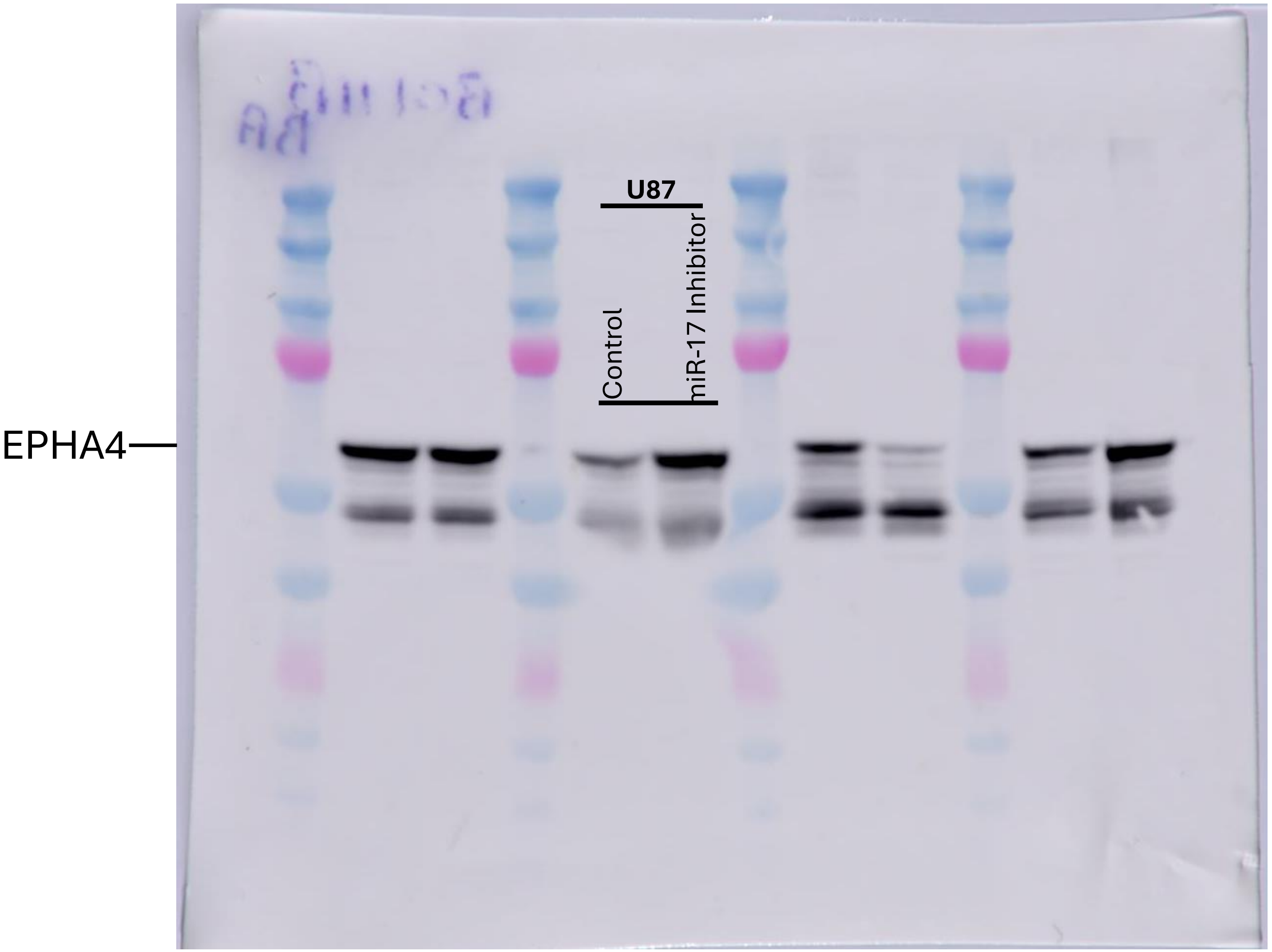

Figure 3A-B

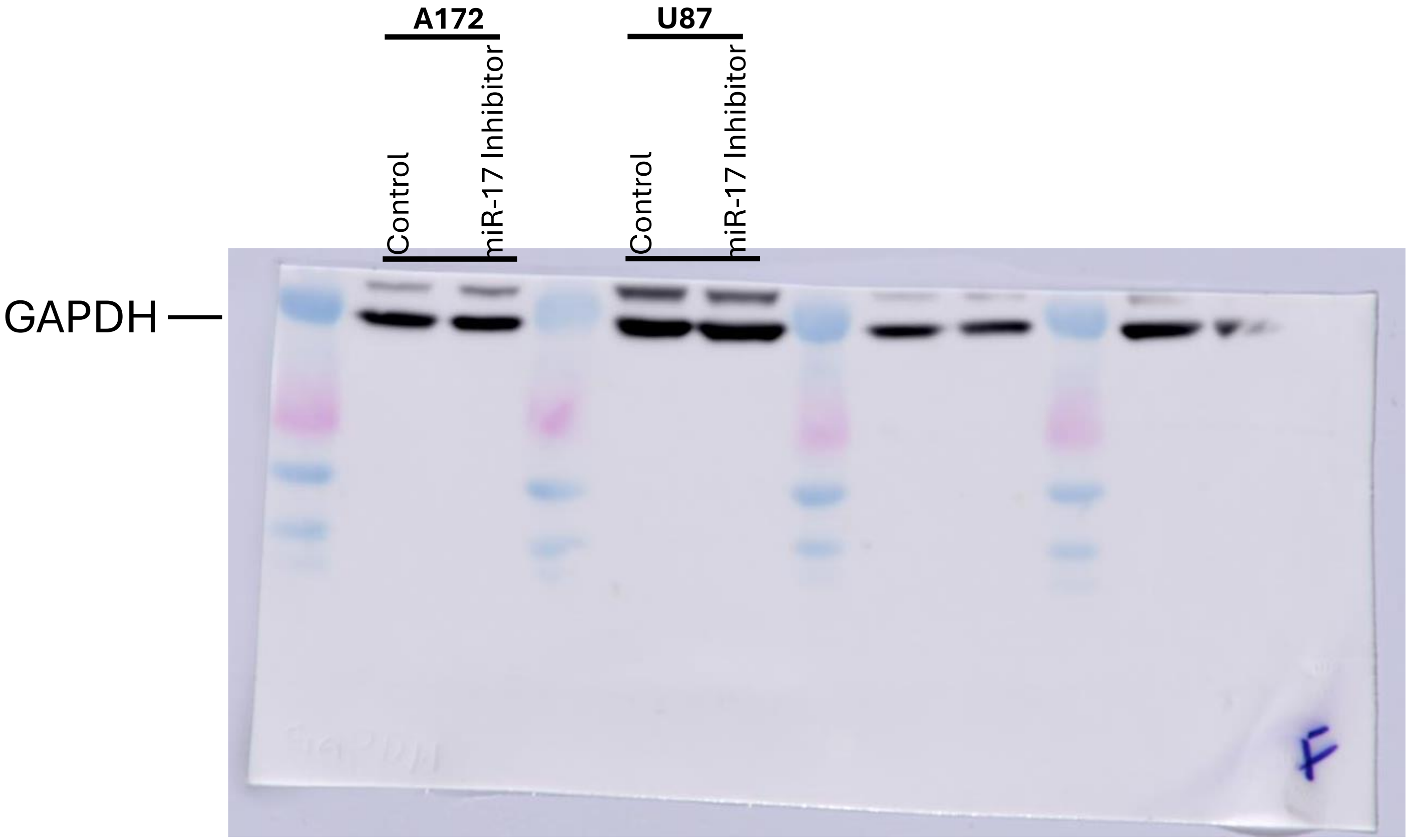
A172
U87
Control
miR-17 Inhibitor
Control
miR-17 Inhibitor
GAPDH
F

Figure 3C-D

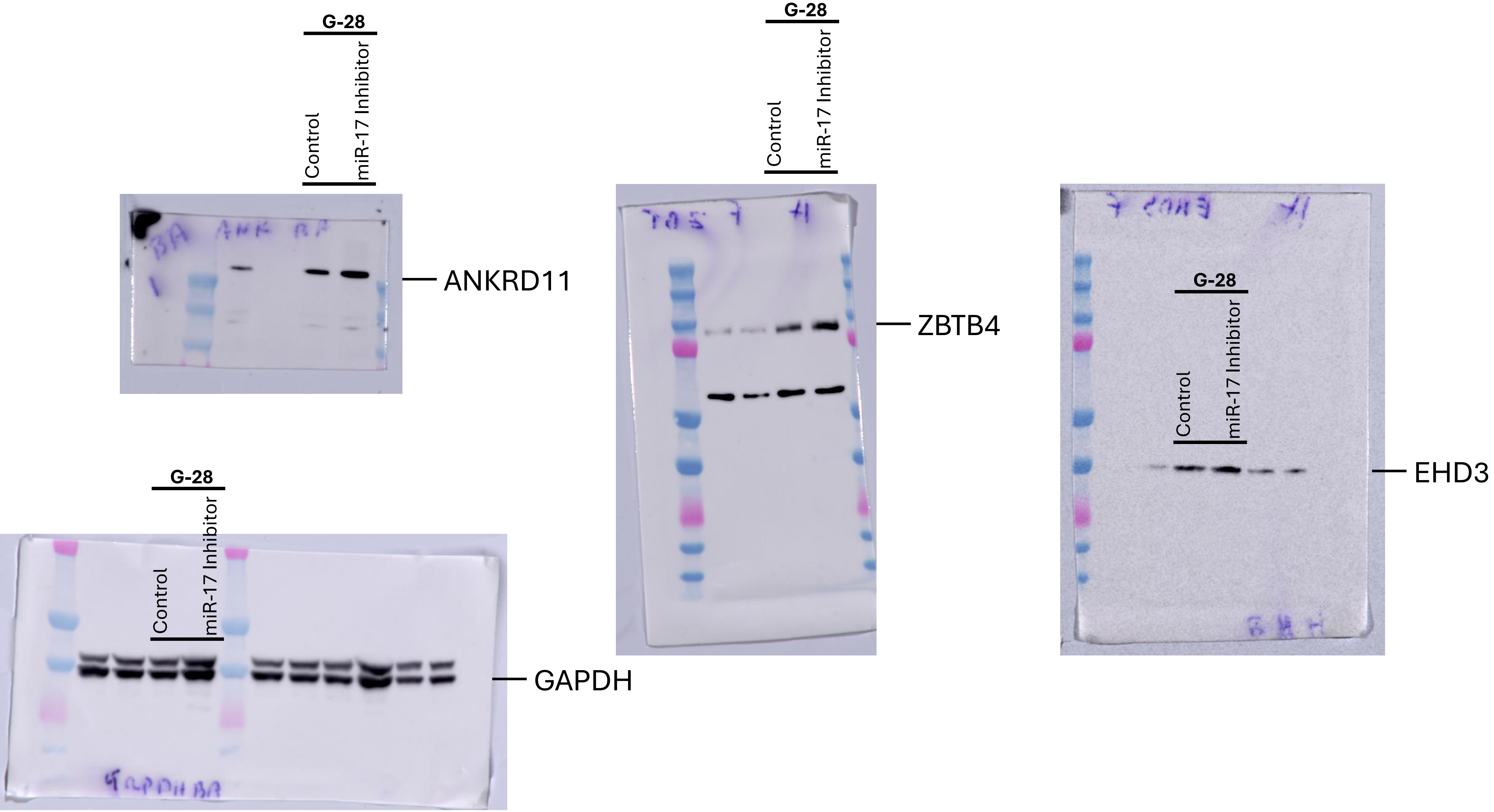
G-28
Control
miR-17 Inhibitor
ANKRD11
G-28
Control
miR-17 Inhibitor
ZBTB4
G-28
Control
miR-17 Inhibitor
EHD3
G-28
Control
miR-17 Inhibitor
GAPDH

Figure 3C-D

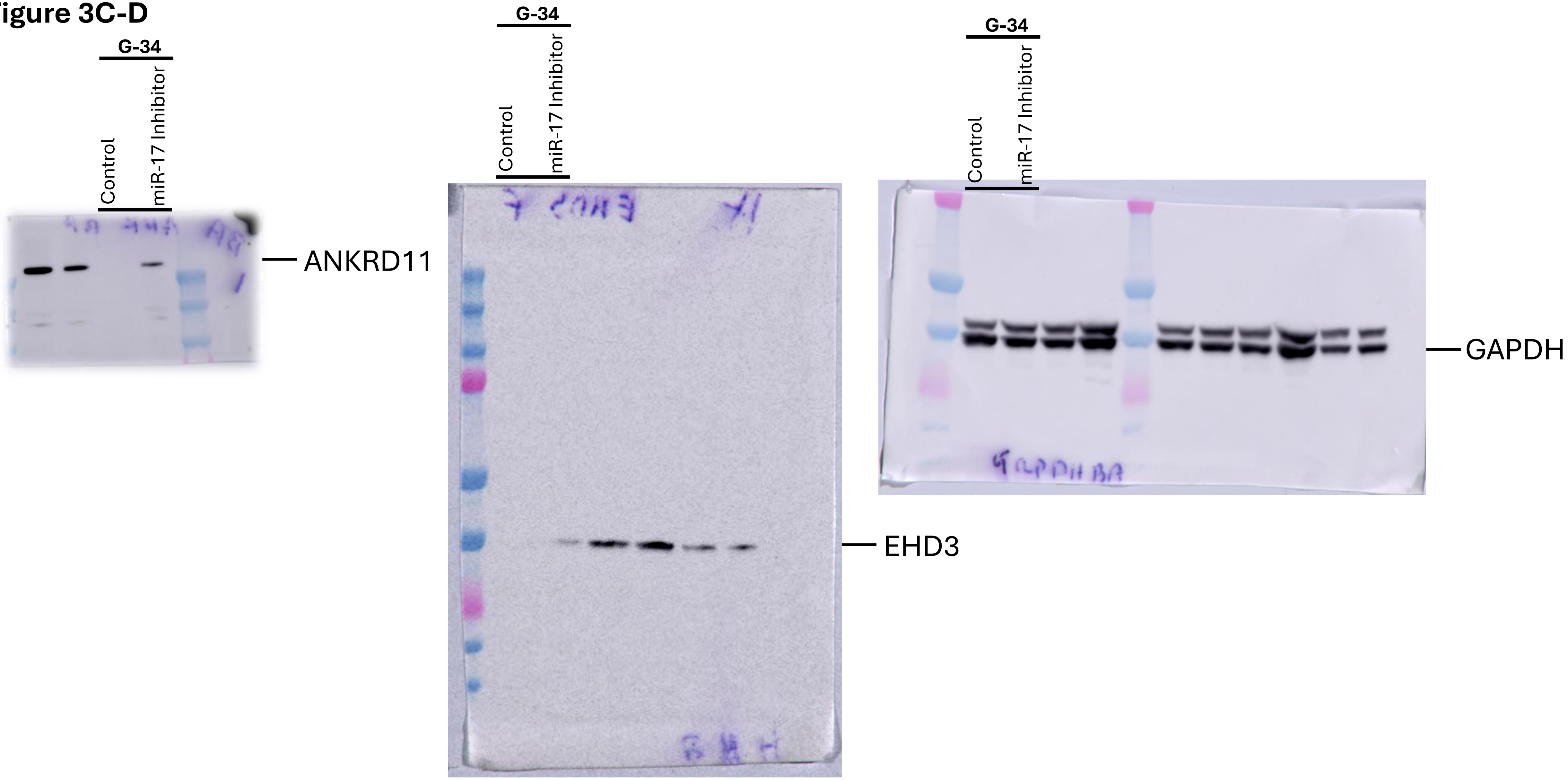

G-34
Control
miR-17 Inhibitor
ANKRD11
G-34
Control
miR-17 Inhibitor
EHD3
G-34
Control
miR-17 Inhibitor
GAPDH

Figure 8L

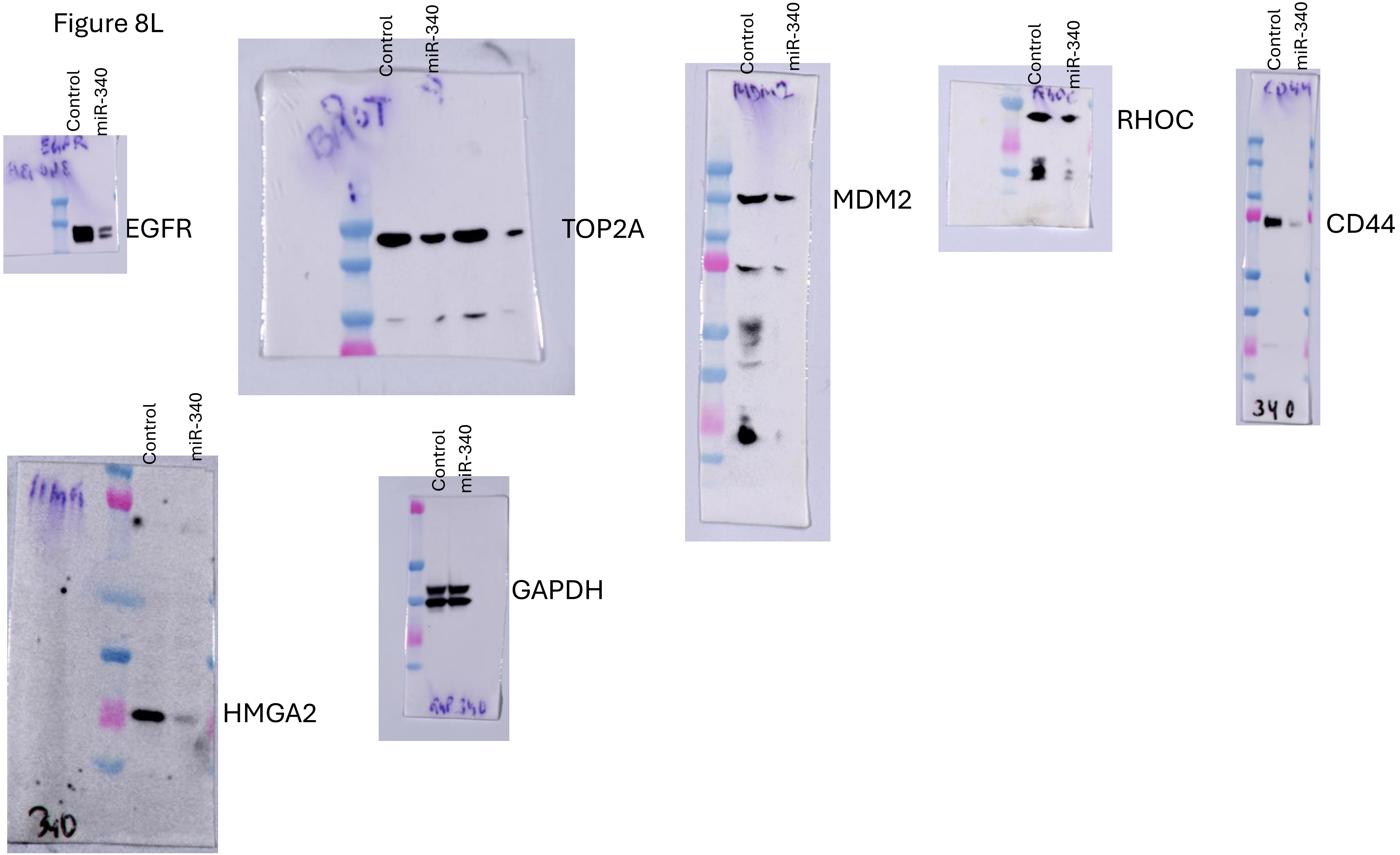

Figure 8M

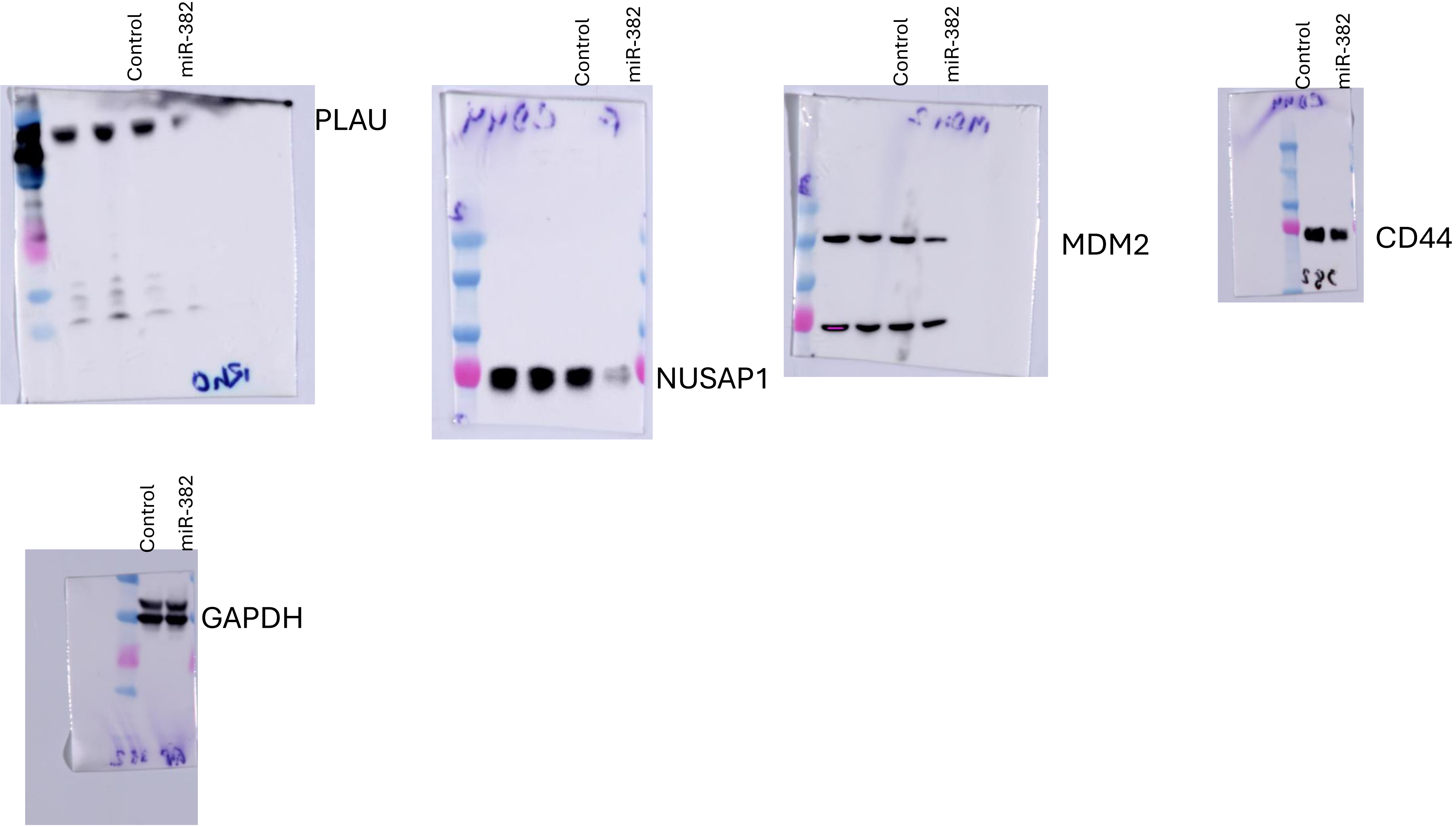
Control
miR-382
PLAU
Control
miR-382
NUSAP1
Control
miR-382
MDM2
Control
miR-382
CD44
Control
miR-382
GAPDH

Figure 8N

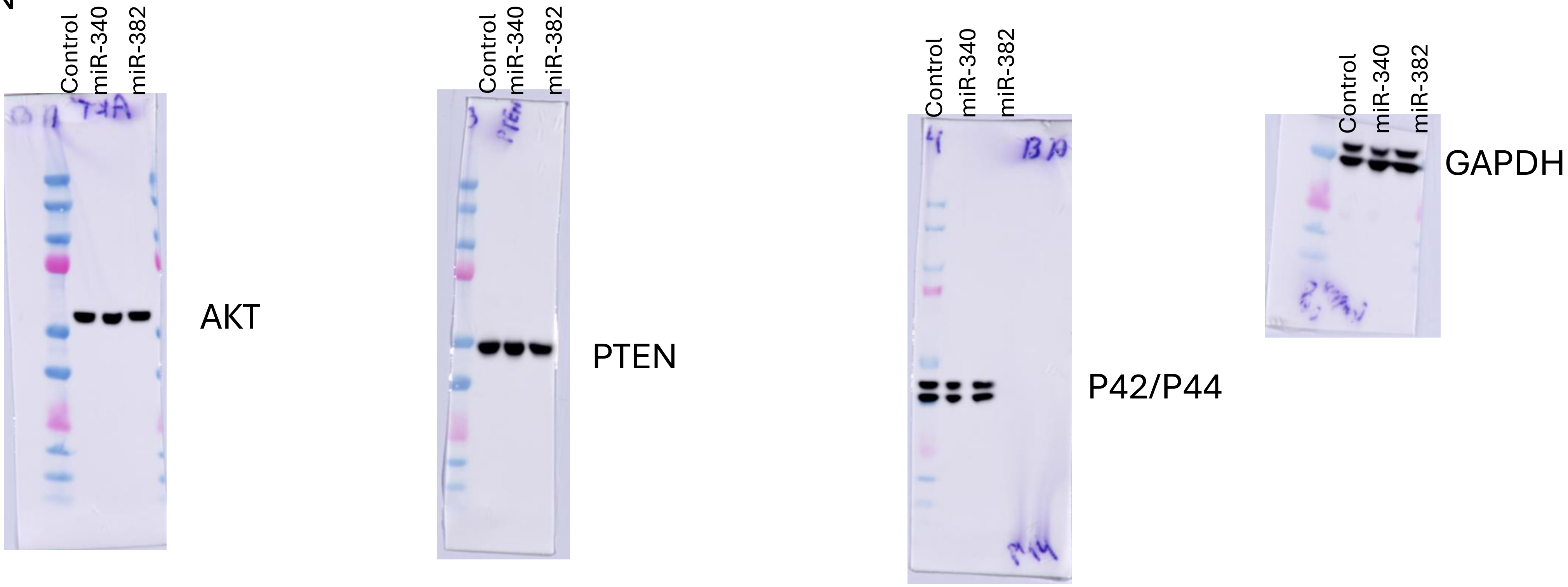

Control
miR-340
miR-382
AKT
Control
miR-340
miR-382
PTEN
Control
miR-340
miR-382
P42/P44
Control
miR-340
miR-382
GAPDH
